# Mindfulness vs Cognitive Behavioral Therapy for Chronic Low Back Pain Treated With Opioids: A Randomized Clinical Trial

**DOI:** 10.1001/jamanetworkopen.2025.3204

**Published:** 2025-04-07

**Authors:** Aleksandra E. Zgierska, Robert R. Edwards, Bruce Barrett, Cindy A. Burzinski, Robert N. Jamison, Yoshio Nakamura, Mary F. Henningfield, Wen-Jan Tuan, Chan Shen, Nalini Sehgal, Robert P. Lennon, Huamei Dong, Vernon M. Chinchilli, Yuxin Liu, Alyssa Turnquist, Anthony R. Schiefelbein, Elizabeth A. Jacobs, Christin Veasley, Penney Cowan, Eric L. Garland

**Affiliations:** 1 Department of Family and Community Medicine, Penn State College of Medicine, Hershey, Pennsylvania; 2 Department of Public Health Sciences, Penn State College of Medicine, Hershey, Pennsylvania; 3 Department of Anesthesiology and Perioperative Medicine, Penn State College of Medicine, Hershey, Pennsylvania; 4 Department of Anesthesiology, Perioperative and Pain Medicine, Harvard Medical School, Brigham and Women’s Hospital, Chestnut Hill, Massachusetts; 5 Department of Psychiatry, Harvard Medical School, Brigham and Women’s Hospital, Chestnut Hill, Massachusetts; 6 Department of Family Medicine and Community Health, School of Medicine and Public Health, University of Wisconsin–Madison, Madison; 7 Department of Anesthesiology, Division of Pain Medicine, Pain Research Center, University of Utah School of Medicine, Salt Lake City; 8 Department of Surgery, Penn State College of Medicine, Hershey, Pennsylvania; 9 Department of Orthopedics and Rehabilitation, School of Medicine and Public Health, University of Wisconsin–Madison, Madison; 10 School of Nursing, University of Wisconsin–Madison, Madison; 11 Department of Internal Medicine, University of California, Riverside School of Medicine, Riverside; 12 Chronic Pain Research Alliance, Milwaukee, Wisconsin; 13 American Chronic Pain Association, Overland Park, Kansas; 14 Sanford Institute for Empathy and Compassion, University of California, San Diego, La Jolla; 15 Department of Psychiatry, University of California, San Diego, School of Medicine, La Jolla

## Abstract

**Importance:**

Chronic low back pain (CLBP) can necessitate opioid therapy. Effective approaches to reduce CLBP’s symptoms and opioid-related harms are needed. Cognitive behavioral (CBT) and mindfulness-based (MBT) therapies may be useful but have not been well-studied for opioid-treated CLBP.

**Objective:**

To compare the effectiveness of MBT vs CBT in opioid-treated CLBP, hypothesizing MBT’s superiority.

**Design, Setting, and Participants:**

In this 12-month community partner–informed, partially masked, multisite, randomized clinical trial, participants at primary and specialty care clinics and community settings were randomly assigned (1:1) to MBT or CBT groups. Participants included English-fluent adults (21 years or older), without prior MBT or CBT training, and with moderate-to-severe CLBP (average score ≥3 on the Brief Pain Inventory [BPI]; functional limitation score ≥20 on the Oswestry Disability Index [ODI]), treated with an opioid dosage of at least 15 mg/d of morphine milligram equivalents (MME) for at least 3 months. Outcome data were collected from July 1, 2017, to November 23, 2022. Analysis used the intention-to-treat approach.

**Interventions:**

Manual-based MBT or CBT interventions consisting of 8 weekly therapist-led group sessions and at-home practice.

**Outcomes and Measures:**

Self-reported coprimary (average pain severity, 0-10 [BPI]; functional limitations, 0-100 [ODI]) and secondary (mental and physical health–related quality of life [QOL] on the Medical Outcomes Study 12-Item Short Form Health Survey and opioid dose in MME per day, Timeline Followback) outcomes compared at 6 and 12 months.

**Results:**

Among 6024 screened individuals, 2926 were ineligible, 2328 were eligible, and 770 were enrolled, including 385 in the MBT and 385 in the CBT groups. Of these, 434 participants (56.4%) were female, 647 (84.0%) identified as non-Hispanic ethnicity, and 630 (81.8%) identified as White race; mean (SD) age was 57.8 (11.3) years. The mean (SD) BPI average pain score was 6.1 (1.6) (moderate pain) and the mean (SD) ODI functional limitation score was 47.2 (14.0) (moderate functional limitations), with reduced physical (mean [SD], 28.5 [8.3]) and mental (mean [SD], 42.5 [11.8]) health–related QOL on the SF-12 and high opioid dosage (mean [SD], 177 [1041] MME/d). Over time, each group significantly improved their outcomes, without serious adverse effects. The intention-to-treat linear mixed-effects model analysis did not detect significant between-group differences at 6 and 12 months for pain (0.21 [95% CI, −0.05 to 0.48; *P* = .12] and 0.13 [95% CI, −0.13 to 0.40; *P* = .33], respectively) or function (0.07 [95% CI, −1.80 to 1.93; *P* = .94], and 0.27 [95% CI, −1.59 to 2.12; *P* = .78], respectively) and indicated MBT’s noninferiority relative to CBT on primary outcomes.

**Conclusions and Relevance:**

In this large trial, CLBP-related symptoms improved, while opioid dosage decreased in both MBT and CBT groups at 6 and 12 months. Increasing availability of these safe psychological therapies could help reduce individual and societal burdens of refractory, opioid-treated CLBP.

**Trial Registration:**

ClinicalTrials.gov Identifier: NCT03115359

## Key Points

**Question:**

What is the comparative effectiveness of mindfulness-based therapy (MBT) and standard-of-care cognitive behavioral therapy (CBT) among adults with opioid-treated chronic low back pain (CLBP)?

**Findings:**

In this randomized clinical trial of 770 adults with opioid-treated CLBP, although there were no differences between groups, both the MBT and CBT groups experienced improved pain, function, and health-related quality of life and reduced opioid dosage at 6 and 12 months.

**Meaning:**

Results of the study suggest that both treatments have potential utility in treating adults with refractory CLBP and improving pain, function, and quality-of-life outcomes.

## Teaser

This randomized clinical trial compares the long-term impact of mindfulness-based therapy (MBT) vs cognitive behavioral therapy (CBT) in patients with opioid-treated chronic lower back pain.

## Introduction

Chronic noncancer pain, a leading cause of disability and reduced quality of life (QOL), costs the US over $600 billion annually and impacts an estimated 50 million US adults, with 17 million experiencing function-limiting, high-impact chronic pain.^1,2,3^ Despite usual care, many patients experience refractory symptoms treated with long-term opioid therapy.^2,3,4^ Chronic low back pain (CLBP) is the most common opioid-treated chronic noncancer pain.^5^ Many patients receiving long-term opioid therapy experience inadequate relief, compromised QOL, and cooccurring mental health challenges, with substantial attendant risk for opioid-associated harms.^4,5,6^ Patients with opioid-treated high-impact chronic pain derive less benefit from existing treatments^7^ and urgently need new interventions. Psychologically oriented treatments are particularly well-suited but have been underused for CLBP due in part to limited access and insurance coverage.

Cognitive behavioral therapy (CBT) is the standard psychological treatment for chronic pain, with demonstrated modest short-term benefits for improving pain, affect, and function in CLBP; long-term benefits of CBT, however, have not been well-studied in populations receiving opioid treatment.^8,9,10^ CBT involves active self-management training to improve coping by adaptively changing behavior, cognition, and affect.^11,12,13^ Mindfulness-based therapy (MBT) is a complementary and integrative health modality successfully applied to chronic pain and its comorbidities, including CLBP and opioid-treated pain.^14,15,16^ Mindfulness, by cultivating nonjudgmental, nonreactive meta-awareness of present-moment experiences (bodily sensations, thoughts, emotions),^16^ offers skills for developing acceptance-based pain coping that differ from those taught by CBT.^17^ To date, 17 studies have evaluated MBT for CLBP, with 3 comparing MBT to CBT^18^; however, given likely small effect-size differences between MBT and CBT, these studies may have been underpowered. No fully powered trials have compared the long-term effectiveness of MBT vs CBT specifically for opioid-treated CLBP.

With usual care failing many patients and dose-dependent opioid-related harms,^2,3,4^ assessment of long-term benefits and comparative effectiveness of MBT and CBT in opioid-treated chronic pain is critical. We conducted a randomized clinical trial (RCT), Strategies to Assist With Management of Pain (STAMP), to compare the long-term impact of MBT vs CBT in opioid-treated CLBP.

## Methods

### Design

This study was a partially masked allocation-concealed 2-arm multisite RCT comparing the effectiveness of MBT and CBT, adjunctive to usual care, among adults with opioid-treated CLBP.^19^ Given the analgesic mechanisms of mindfulness,^16^ we hypothesized that, compared with CBT, the MBT group would report greater improvements in pain and function (aim 1) and QOL and reduced opioid dosage (aim 2) at 6 and 12 months post entry. The trial protocol was published elsewhere^19^; the protocol submitted for study approval is available in Supplement 1.

The first participant was enrolled July 1, 2017, and the last on August 4, 2021, when enrollment goals were reached. Outcome data collection was completed on November 23, 2022. The protocol was approved by the Institutional Review Boards (IRBs) of the involved institutions, with the University of Wisconsin–Madison’s IRB as the IRB of record. A certificate of confidentiality was provided by the National Institutes of Health. A data and safety monitoring committee reviewed study progress and participant safety twice yearly. Participants completed written informed consent procedures and were reimbursed up to $390 for their time and effort (prorated to the number of tasks completed).

The study followed the recommendations for clinical trials in chronic pain^20,21^ and for fidelity monitoring, enhancement, and reporting of behavioral interventions,^22^ and the Consolidated Standards of Reporting Trials (CONSORT) reporting guideline. Community-partner input informed the study design, implementation, and result interpretation.^19^

### Randomization

The study statistician, using computer-generated permuted blocks of random sizes and stratified by study site, randomized participants 1:1 to MBT and CBT and prepared sealed envelopes (concealed allocation). Envelopes were distributed consecutively by a study coordinator (including A.T. and C.A.B.) to participants after baseline assessments.

### Masking

Investigators, analysts, and main outcome assessors were masked to the randomization status. Therapists and participants were not masked.

### Protocol Changes

The main study design and outcomes did not change. Approved minor protocol changes, detailed elsewhere,^19^ included (1) in October 2018, eligibility criteria were changed to reduce the minimum opioid dosage from at least 30 to at least 15 morphine milligram equivalents (MME)/d, as justified by increased opioid therapy tapering in the US; (2) in-person activities were suspended in March 2020; all-virtual study conduct was approved in October 2020; (3) extension of the study’s end-date from December 2021 to April 2023 was based on recruitment challenges compounded by the pandemic; and (4) in March 2023, a statistical analysis plan was approved by the funding agency for post hoc noninferiority analysis, developed by investigators masked to study results, to assess for noninferiority of MBT vs standard-of-care CBT on primary outcomes, should the superiority analysis not find significant between-group differences.

### Participants

Research teams in Madison, Wisconsin; Boston, Massachusetts; and Salt Lake City, Utah; recruited participants from the community and outpatient settings, primarily by posting study flyers and partnering with clinics to identify prospective participants. Eligible individuals were English-fluent adults 21 years or older with opioid-treated CLBP (defined as daily pain in lumbosacral region or sciatica, treated with ≥15 MME/d for ≥3 months), average daily pain intensity of at least 3 on the Brief Pain Inventory (BPI),^23^ and CLBP-related functional limitations score of at least 21 on the Oswestry Disability Index (ODI).^24^ Exclusion criteria included formal training or MBT and/or CBT practice, current pregnancy, psychiatric disorders with past-year psychotic symptoms, or inability to safely and reliably participate in the study.

### Interventions

Intervention-related details are described elsewhere.^19^ Briefly, participants received the MBT or CBT interventions adjunctive to usual care for CLBP that was provided by each participant’s regular clinician. Intervention development and fidelity monitoring were guideline based.^22^ The manualized interventions were matched in format and therapist-participant contact time and consisted of 8 weekly 2-hour therapist-led group sessions (in person until March 2020, then remote), tailored to the needs of persons with opioid-treated CLBP, and recommended home practice of at least 30 min/d, 6 d/wk, during the study.^19^ MBT, patterned after existing curricula^25,26,27,28^ and pilot tested, included training in mindfulness skills and applied mindfulness to deconstruct pain into constituent cognitive, emotional, and sensorial components and adaptively cope with pain and negative emotions. MBT explicitly encouraged applying mindfulness before deciding whether to take an as-needed opioid medication so that a mindful pause could disrupt automaticity and help decrease opioid use.^28^ CBT, adapted from existing programs,^11,12,13^ included a variety of cognitive-behavioral strategies (eg, cognitive restructuring of maladaptive pain-related beliefs, coping skills, relaxation training, and behavioral activation, including pacing) meant to facilitate active self-management.

Therapists were experienced in MBT or CBT delivery and trained in the intervention protocol.^19^ Intervention adherence by the therapist, measured with a checklist assessing the presence of session’s key components, was developed by the study team based on the existing scale^29^ and completed by therapists and research staff present at the sessions. The therapists adhered to 98.1% of the checklist items (97.9% for MBT and 98.2% for CBT) across study interventions.

### Measures

Outcome measures were collected using validated guideline-recommended questionnaires^20,21^ and participants’ self-entry into the research database at baseline and 3, 6, 9, and 12 months after treatment initiation. Coprimary measures included past week’s average pain severity (BPI; ranging from 0-10 on a numerical rating scale, with 0 indicating no pain^23^) and back pain-related functional limitations “today” (ODI; ranging from 0-100 total score, with 0 indicating no limitations^24^). Secondary measures included mental and physical health–related QOL from the Medical Outcomes Study 12-Item Short Form Health Survey (SF-12; total score ranging from 0-100, with 0 indicating worst QOL)^30^ and daily opioid dose (Timeline Followback [TLFB]^31^; measured as average MME per day during the past 14 days). Daily opioid dose was calculated by converting the doses of participant-reported opioid medications to MME, using published conversion factors and approaches.^32^

Other measures included self-reported baseline demographic characteristics, including gender and race and ethnicity per NIH guidance, CLBP-related characteristics, use of health care services, professional and personal productivity,^19^ BPI-based 7-item pain interference,^23^ and opioid dose categories. Participant logs of home practice^19^ (minutes per day during the past 14 days) were collected at follow-ups. Adverse event information was systematically collected at each assessment and inquired about at each contact.

### Sample Size

A sample of 766 participants (383 per arm) was estimated to provide 80% power to detect an effect size Cohen *d* of 0.25 for MBT vs CBT for pain severity and function, with α = .025 for each coprimary outcome (for an overall α = .05). This sample size accounted for attrition of up to 20%.

### Statistical Analysis

The analyses were completed by masked study statisticians (C.S., H.D., V.M.C., and Y.L.) using R software, version 4.3.1 (R Program for Statistical Computing). Descriptive statistics characterized each group at each time point, with results presented as mean (SD) or percentages, unless otherwise indicated. Comparison of baseline data assessed for between-group differences; *P* values were computed under the false discovery rate to reduce the number of potential type I errors with multiple comparisons, with 2-sided *P* < .05 signifying statistical significance.

Intention-to-treat analysis used linear mixed-effects model (LMEM) with restricted maximum likelihood estimation to compare the impact of MBT relative to CBT (estimate of the difference in questionnaire score or opioid dose change with 95% CI and *P* value) at 6 and 12 months. A 2-tailed *P* < .025 was applied to each coprimary outcome and 2-tailed *P* < .05 to each secondary outcome. Per our a priori analysis plan^19^ (Supplement 2), each model included a dummy variable for treatment, effect variables for time and site, time × treatment interaction, and a random intercept for participant. If the primary outcome analysis did not detect significant between-group differences, a post hoc noninferiority analysis was applied, based on 95% CIs of the coprimary outcome change scores derived from the primary LMEM analysis, with the margin of acceptable difference (Δ) set at 0.8 for BPI average pain scores and 8.0 for the ODI-based function scores, guided by external experts and existing literature.^20,21^ We also explored the potential effects of in-person vs virtual intervention delivery by allowing interaction of delivery mode with time × treatment. Generalized linear mixed-effects model with logit link function was applied to binary outcomes (eg, opioid dosage ≥90 MME/d), with odds ratios, 95% CIs, and *P* values reported.

For individual partially completed primary and secondary outcome surveys, we applied a published approach to missing values or imputed missing values if at least 75% of responses to a given survey were provided.^33^ The restricted maximum likelihood estimates from LMEM yielded valid estimates in the presence of missing at random data; our inspection of the data did not identify evidence for missing not-at-random data. To assess LMEM’s normality assumption, normal q-q plots of the residuals were used; log transformation was applied to the nonnormally distributed daily MME variable.

## Results

### Study Sample

**Figure 1.**
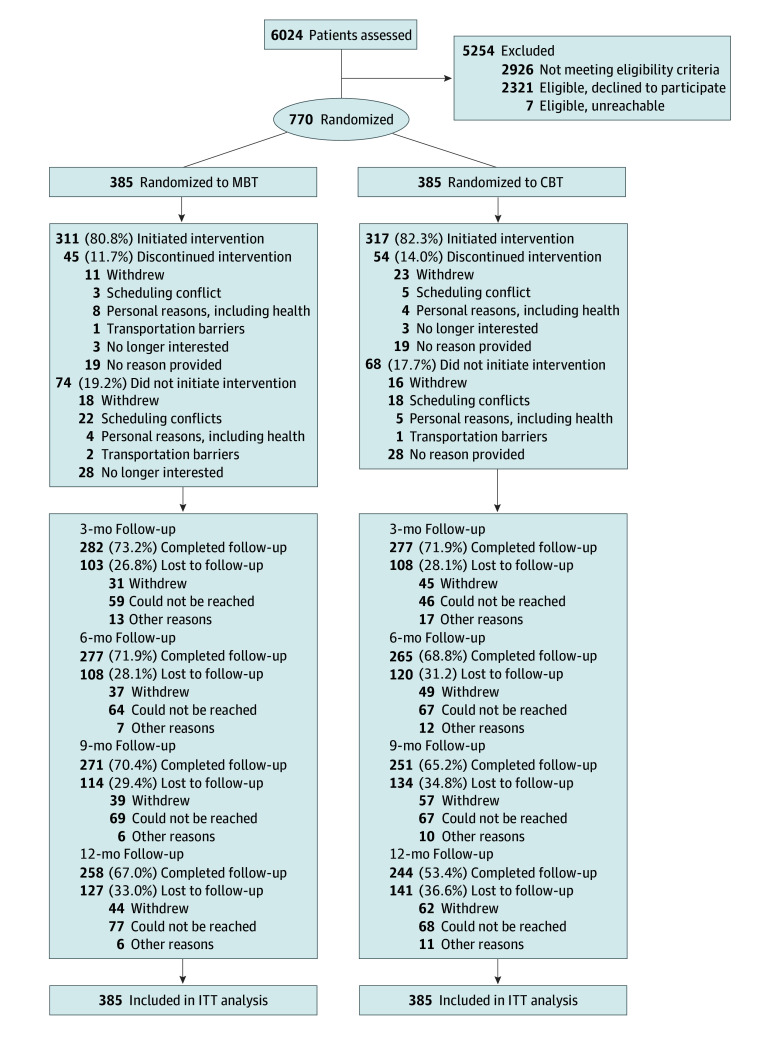
The Strategies to Assist With Management of Pain Study Participant Flow CBT indicates cognitive behavioral therapy; ITT, intention to treat; and MBT, mindfulness-based therapy.

After screening 6024 persons, 2926 were ineligible, 2328 were eligible, and 770 adults (385 in the MBT and 385 in the CBT groups) provided informed consent and were enrolled, randomized, and included in the analysis. Primary outcome data were provided by 542 participants (70.4%) at 6 months and 502 (65.2%) at 12 months (Figure 1). The rates of loss-to-follow-up, session attendance, and weekly minutes of home practice during the study were not significantly different between the MBT and CBT groups.

A total of 434 participants (56.4%) identified as female and 328 (42.6%) as male. Most participants identified as non-Hispanic or non-Latino ethnicity (647 [84.0%]) compared with 35 (4.5%) Hispanic; in terms of race, 71 (9.2%) identified as Black, 630 (81.8%) as White, and 56 (7.3%) as other (including American Indian or Alaska Native, Asian, Native Hawaiian or Other Pacific Islander, unknown, other, and declined). The mean (SD) sample age was 57.8 (11.3) years. Participants reported moderate average pain scores (mean [SD] BPI, 6.1 [1.6]) and functional limitations (mean [SD] ODI, 47.2 [14.0]), compromised physical (SF-12 component, 28.5 [8.3]) and mental (SF-12 component, 42.5 [11.8]) health–related QOL, and high mean opioid dosages (177 [1041] MME/d) (Table 1), without significant between-group differences. One-third of the sample was treated with a dosage of at least 90 MME/d, and, overall, they reported long-standing CLBP, numerous prior treatments for CLBP, with a high proportion reporting outpatient, emergency department, and inpatient care and inability to work at home or professionally in the prior 6 months (eTable 1 in Supplement 3).

**Table 1.** Baseline Characteristics of the Study Participants^a^

Characteristic	Participant group		
	All (N = 770)	MBT (n = 385)	CBT (n = 385)
Demographic			
Gender, No. (%)			
Female	434 (56.4)	233 (60.5)	201 (52.2)
Male	328 (42.6)	147 (38.2)	181 (47.0)
Age, mean (SD), y	57.8 (11.3)	57.6 (11.0)	58.0 (11.7)
Ethnicity, No. (%)			
Hispanic or Latino	35 (4.5)	17 (4.4)	18 (4.7)
Non-Hispanic	647 (84.0)	316 (82.1)	331 (86.0)
Unknown	20 (2.6)	13 (3.4)	7 (1.8)
Race, No. (%)			
Black	71 (9.2)	32 (8.3)	39 (10.1)
White	630 (81.8)	314 (81.6)	316 (82.1)
Other^b^	56 (7.3)	34 (8.8)	22 (5.7)
Employment status, No. (%)			
Disabled (any reason)	342 (44.4)	181 (47.0)	161 (41.8)
Retired	163 (21.2)	73 (19.0)	90 (23.4)
Unemployed	47 (6.1)	23 (6.0)	24 (6.2)
Working now	137 (17.8)	66 (17.1)	71 (18.4)
Other	70 (9.1)	35 (9.1)	35 (9.1)
Clinical			
Average pain severity BPI score, mean (SD)^c^	6.07 (1.60)	5.98 (1.53)	6.17 (1.66)
Back pain-related disability ODI score, mean (SD)^d^	47.15 (13.96)	47.30 (13.64)	47.00 (14.29)
Health-related SF-12 QOL score, mean (SD)^e^			
Physical health component score	28.48 (8.29)	28.75 (8.13)	28.21 (8.45)
Mental health component score	42.45 (11.81)	41.61 (11.15)	43.29 (12.40)
Daily opioid dose in past 14 d, mean (SD)^f^			
MME/d	177.19 (1040.64)	163.12 (601.07)	191.27 (1344.25)
Log-transformed MME/d	5.72 (1.78)	5.76 (1.79)	5.67 (1.77)

Abbreviations: BPI, Brief Pain Inventory; CBT, cognitive behavioral therapy; MBT, mindfulness-based therapy; MME, morphine milligram equivalent; ODI, Oswestry Disability Index; QOL, quality of life; SF-12, 12-Item Short Form Health Survey.

^a^ The frequencies and percentages in a given variable category may not total the sample size per group or 100% responses as some participants’ responses were unavailable (eg, missing or marked as declined, unknown or unspecified).

^b^ Includes American Indian or Alaska Native, Asian, Native Hawaiian or Other Pacific Islander, decline, unknown, or other.

^c^ Rated with a single BPI item, with scores ranging from 0 to 10, and higher scores indicating greater severity.

^d^ Scores range from 0 to 100, with higher scores indicating greater limitations.

^e^ Scores range from 0 to 100, with higher scores indicating better QOL.

^f^ Collected using the Timeline Followback method.

### Within-Group Longitudinal Change

Compared with baseline, both groups significantly improved in primary and secondary outcomes at 6 and 12 months (Table 2) and, in general, during the 12-month follow-up (eTable 2 in Supplement 3). For example, the change in BPI score for average pain from baseline in the MBT group was −0.35 (95% CI, −0.54 to −0.17) at 6 months and −0.45 (95% CI, −0.64 to −0.26) at 12 months (*P* < .001 for both); for the CBT group, change from baseline was −0.57 (95% CI, −0.76 to −0.38) at 6 months and −0.59 (95% CI, −0.78 to −0.40) at 12 months (*P* < .001 for both). The change in ODI score for functional limitations from baseline in the MBT group was −2.15 (95% CI, −3.41 to −0.89) at 6 months and −3.19 (95% CI, −4.45 to −1.93) at 12 months (*P* < .001 for both); for the CBT group, change from baseline was −2.24 (95% CI, −3.62 to −0.86; *P* = .002) at 6 months and −3.49 (95% CI, −4.86 to −2.12; *P* < .001) at 12 months.

**Table 2.** Change in Main Outcomes

Variable	Participant group			
	MBT		CBT	
	Change from baseline	*P* value	Change from baseline	*P* value
**Average pain severity, BPI score**				
6-mo Score, mean (SD)	5.55 (1.65)	<.001	5.54 (1.86)	<.001
∆ score, mean (95% CI)	−0.35 (−0.54 to −0.17)		−0.57 (−0.76 to −0.38)	
12-mo Score, mean (SD)	5.44 (1.85)	<.001	5.50 (1.98)	<.001
∆ score, mean (95% CI)	−0.45 (−0.64 to −0.26)		−0.59 (−0.78 to −0.40)	
**Functional limitations, ODI score**				
6-mo Score, mean (SD)	44.64 (16.05)	.001	45.12 (16.44)	.002
∆ score, mean (95% CI)	−2.15 (−3.41 to −0.89)		−2.24 (−3.62 to −0.86)	
12-mo Score, mean (SD)	43.41 (15.32)	<.001	43.17 (15.43)	<.001
∆ score, mean (95% CI)	−3.19 (−4.45 to −1.93)		−3.49 (−4.86 to −2.12)	
**Health-related quality of life, SF-12 score**				
Mental health component				
6-mo Score, mean (SD)	43.21 (11.42)	.02	43.45 (12.14)	.54
∆ score, mean (95% CI)	1.39 (0.22 to 2.55)		−0.41 (−1.71 to 0.90)	
12-mo Score, mean (SD)	43.48 (11.48)	.001	45.91 (11.04)	.002
∆ score, mean (95% CI)	1.91 (0.74 to 3.08)		2.07 (0.78 to 3.36)	
Physical health component				
6-mo Score, mean (SD)	30.66 (8.72)	<.001	29.94 (9.35)	<.001
∆ score, mean (95% CI)	1.87 (1.00 to 2.75)		2.04 (1.11 to 2.98)	
12-mo Score, mean (SD)	30.54 (8.83)	<.001	30.00 (9.21)	<.001
∆ score, mean (95% CI)	1.69 (0.82 to 2.57)		1.66 (0.73 to 2.58)	
**Daily opioid dose (TLFB), MME/d**				
6-mo, Mean (SD)	118.79 (520.99)	<.001	191.38 (1603.24)	<.001
Log-transformed, mean (SD)	5.11 (2.14)		4.92 (2.47)	
∆ log-transformed, mean (95% CI)	−0.54 (−0.69 to −0.38)		−0.67 (−0.84 to −0.49)	
12-mo, Mean (SD)	144.22 (556.19)	<.001	192.31 (1643.92)	<.001
Log-transformed, mean (SD)	4.95 (2.50)		4.84 (2.49)	
∆ log-transformed, mean (95% CI)	−0.79 (−0.95 to −0.63)		−0.81 (−0.99 to −0.64)	

Abbreviations: BPI, Brief Pain Inventory; CBT, cognitive behavioral therapy; ∆, change; MBT, Mindfulness-Based Therapy; MME, morphine milligram equivalents; ODI, Oswestry Disability Index; SF-12, Medical Outcomes Study 12-Item Short Form Health Survey; TLFB, Timeline Followback.

### Between-Group Differences in Longitudinal Change

**Figure 2.**
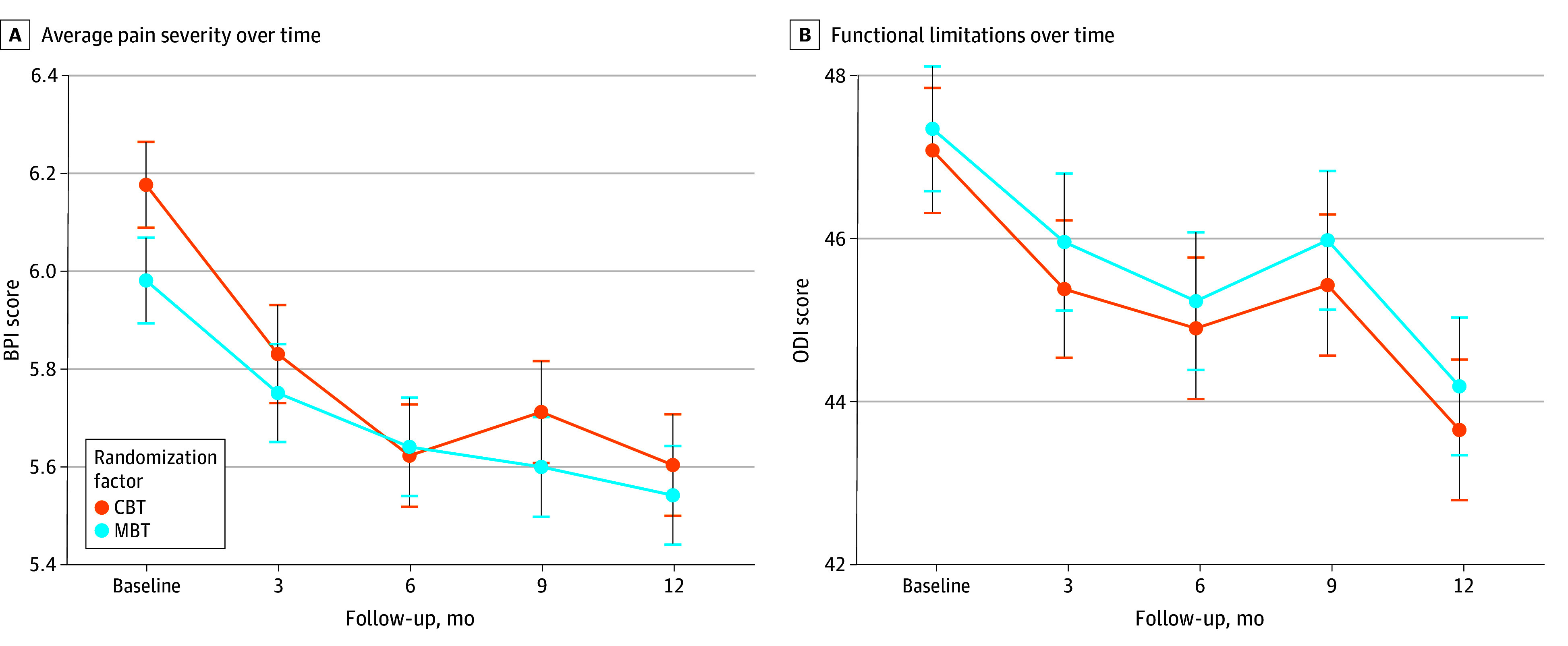
Primary Outcomes by Group Status Over Time A, Average pain score is measured by a single item on the Brief Pain Inventory (BPI; range, 0-10, with higher scores indicating greater severity of average pain). B, Functional limitations are measured using the Oswestry Disability Index (ODI; range, 0-100, with higher scores indicating greater limitations). Significant reductions in values were noted within each group, compared with baseline; however, no statistically significant differences in score change were found between the groups over time. Scores are presented as estimated marginal means and SEs. CBT indicates cognitive behavioral therapy group; MBT, mindfulness-based therapy group. Details on the estimated marginal means and SEs at each follow-up point are included in eTable 4 in Supplement 2.

The LMEM analysis did not find significant between-group differences in change in primary and secondary outcomes at 6 and 12 months (Table 3). Estimates for average pain severity (6 months, 0.21 [95% CI, −0.05 to 0.48; *P* = .12]; 12 months, 0.13 [95% CI, −0.13 to 0.40; *P* = .33) (Figure 2A), functional limitations (6 months, 0.07 [95% CI, −1.80 to 1.93; *P* = .94]; 12 months, 0.27 [95% CI, −1.59 to 2.12; *P* = .78]) (Figure 2B), physical health–related QOL (6 months, −0.16 [95% CI, −1.44 to 1.12; *P* = .81]; 12 months, 0.04 [95% CI, −1.23 to 1.32; *P* = .95]); and log-transformed daily MME (6 months, 0.13 [95% CI, −0.11 to 0.36; *P* = .29]; 12 months, 0.02 [95% CI, 0.02 [95% CI, −0.22 to 0.26; *P* = .88]) were not significantly different. Compared with CBT, improvements in mental health–related QOL favored the MBT group at 6 months (estimate, 1.79 [95% CI, 0.05-3.54]; *P* = .04), but not 12 months (Table 3). Details on the change in the main and additional outcomes at all follow-up assessments are presented in eTable 3 in Supplement 3.

**Table 3.** Linear Mixed Effects Model: Change in Primary Outcomes From Baseline in the MBT vs the CBT Groups^a^

Randomization factor × time factor	Evaluation	
	6 mo	12 mo
**Average pain severity (BPI)**		
Estimate (95% CI)	0.21 (−0.05 to 0.48)	0.13 (−0.13 to 0.40)
*P* value	.12	.33
**Functional limitations (ODI)**		
Estimate (95% CI)	0.07 (−1.80 to 1.93)	0.27 (−1.59 to 2.12)
*P* value	.94	.78
**Health-related quality of life (SF-12)**		
Mental health component		
Estimate (95% CI)	1.79 (0.05 to 3.54)	−0.15 (−1.89 to 1.59)
*P* value	.04	.86
Physical health component		
Estimate (95% CI)	−0.16 (−1.44 to 1.12)	0.04 (−1.23 to 1.32)
*P* value	.81	.95
**Daily opioid dose in past 14 d (TLFB)**		
Log-transformed MME/d, estimate (95% CI)	0.13 (−0.11 to 0.36)	0.02 (−0.22 to 0.26)
*P* value	.29	.88

Abbreviations: BPI, Brief Pain Inventory; MME, morphine milligram equivalents; ODI, Oswestry Disability Index; SF-12, Medical Outcomes Study 12-Item Short Form Health Survey; TLFB, Timeline Followback.

^a^ The model included a dummy variable for treatment, an effect for each time and site, time × treatment interaction, and a random intercept for participant. The cognitive behavioral therapy group was the reference group.

Post hoc noninferiority analysis indicated noninferiority of improvements in primary outcomes in the MBT compared with the CBT group at 6 and 12 months (eFigure in Supplement 3). We also did not find between-group differences over time in pain interference or percentage of individuals treated with high MME dosages (eTable 3 in Supplement 3) or when accounting for the prespecified subgroup status based on gender or intervention delivery mode (in person vs virtual). No serious adverse events were reported in either group.

## Discussion

Participants with opioid-treated CLBP in the MBT and CBT groups reported significant reductions in pain and opioid dose and increased function and health-related QOL through 12 months, without between-group differences. Post hoc noninferiority analysis found comparable pain and functional improvements in MBT, relative to CBT, the current gold standard psychotherapy for chronic pain.^9,10^

To our knowledge, this RCT was the largest trial to date comparing MBT with CBT, with a longer follow-up than previous trials of MBTs for opioid-treated pain.^16,18^ Study results indicated that both MBT and CBT were associated with lasting benefits in refractory, complex CLBP. These improvements are noteworthy in the context of the moderate-to-severe, high-impact, opioid-treated CLBP, which usually worsens over time rather than improves.^2,3,4^ Although the lack of a usual care control group may raise questions about efficacy and risks the possibility that the results arose from regression to the mean in both groups, existing research, clinical practice-based evidence, experiences of our community partners, and qualitative reports from our participants themselves^34^ corroborate the interpretation that participants’ improvements, across both groups, were associated with the study interventions. Longitudinal studies report high-impact chronic pain, including opioid-treated CLBP, is progressive and unlikely to improve spontaneously, with usual care.^2,3,4^ A recent study of remotely delivered MBTs found reductions in pain severity and interference at 1 year, compared with usual care, among veterans with chronic pain.^35^ We conclude that MBT and CBT should be considered the first-line nonpharmacological treatment options for opioid-treated CLBP; ideally, both would be available so that individuals could select their preferred treatment.

These findings contribute to the growing evidence on MBT and CBT effectiveness for chronic pain, including CLBP, with results extending to opioid-treated populations.^9,10,14,15,16,18,36,37^ Prior to this trial, Mindfulness Oriented Recovery Enhancement (MORE)^28^ was the only MBT to demonstrate efficacy across several studies in concurrently reducing chronic pain symptoms and opioid dose. In 2 efficacy trials, MORE significantly outperformed an active control condition,^38,39^ evidence of superiority that we did not detect in our study. This discrepancy could be explained by differences in specific intervention components between MORE and the STAMP’s MBT, or the design of the STAMP study, which compared MBT with CBT, a comparator that was likely more therapeutically active than the supportive psychotherapy control used in the MORE’s RCTs.^38,39^ Cherkin et al^36^ compared CBT and MBT against usual care for CLBP and observed that both groups outperformed usual care without significant differences between CBT and MBT at 26 weeks; however, opioid use was reported by only 11% of participants.

Our quantitative findings were corroborated by participant qualitative reports^34^ and existing reviews and meta-analyses,^16,18^ laying a foundation for establishing MBT and CBT as part of standard chronic pain care. Participant-reported benefits did not differ based on intervention delivery modality (in person vs virtual), and remote delivery of MBTs has been shown effective in chronic pain,^35^ highlighting the potential for telehealth to enhance intervention accessibility and scalability. Our findings support expanding insurance coverage to include MBT and CBT as first-line treatments and as means for increasing patients’ engagement in their own personalized pain care.

### Generalizability

The study design with broad eligibility criteria should generalize to clinical populations; however, factors inherent to research (eg, close follow-up, financial reimbursements for participation, transportation assistance) are not typically implemented in clinical care settings. Our sample, comprising mostly White adults, may limit generalizability to other racial and ethnic groups.

### Future Directions

MBT and CBT were associated with safe improvements in pain and functional outcomes among adults affected with refractory, complex, opioid-treated CLBP. We need to better understand how to support dissemination and implementation of evidence-based psychological treatments for chronic pain and their coverage by health plans. The similar magnitude of improvements in the MBT and CBT groups may reflect common mechanisms of action between these 2 interventions, as both focus on training in pain-relevant self-management skills to regulate cognition, emotions, and behavior, despite differences in their primary therapeutic focus and content. Future studies might identify the most therapeutically active components of each intervention to enable the design of briefer interventions^40^ or allow tailoring different treatments to individuals. In addition, little is known about whether the benefits of these interventions can be maintained past 1 year and related to adherence. Finally, treatment response moderator studies could help identify patients who benefit most from MBT or CBT. Although subpar treatment fidelity is a common threat to validity, especially in studies of complex behavioral interventions, we applied a guideline-recommended, multifaceted approach,^22^ reaching high therapist adherence to the intervention protocol.

### Limitations

Despite a large sample size and long-term follow-up, the study has limitations. This patient-centered trial did not include a usual care control group, raising the possibility of regression to the mean. Enrollment from diverse ethnic and racial groups and study retention were lower than anticipated. Although the COVID-19 pandemic likely contributed to reduced adherence, better support for participant engagement may be needed, especially for those from underrepresented groups or with complex health problems. The trial retention rate was lower than we had estimated and used for sample size calculations, raising the possibility that our sample was not fully powered to detect between-group differences in primary outcomes, even if they existed. While a centralized process with real-time randomization would have been optimal, our approach allowed for effective masking.

## Conclusions

In this largest, to our knowledge, RCT of MBT vs CBT for opioid-treated chronic pain to date, both MBT and CBT groups experienced significant, comparable improvements in CLBP and reductions in opioid dose, sustained through 12 months, without serious adverse effects. MBT, like CBT, should be considered a treatment option to integrate into standard-of-care for complex, refractory, opioid-treated CLBP. Both CBT and MBT should be accessible to reduce the burdens of CLBP and opioid-related harm.

## Notes

Supplement 1.
Trial Protocol

Supplement 2.
Original Statistical Analysis Plan, Modification, and Final Statistical Analysis Plan Summary

Supplement 3. **eTable 1.** Additional Baseline Characteristics of the Study Participants: Total Sample and by Group Status **eTable 2.** Change in Main Outcomes (Raw Values and Difference Scores) Over Time by Group Status **eTable 3.** Linear Mixed Effects Model (Primary Outcome Analysis): Change in Main Outcomes From Baseline in the MBT vs the CBT Group **eTable 4.** Primary Outcomes Over Time (Estimated Marginal Means and SEs): Variables for Figure 2 **eFigure.** Between-Group Differences in Primary Outcomes Over Time: Noninferiority Analysis

Supplement 4.
Data Sharing Statement
