# Supplementary material for: Mindfulness vs Cognitive Behavioral Therapy for Chronic Low Back Pain Treated With Opioids: A Randomized Clinical Trial
Source: JAMA Netw Open. 2025 Apr 7;8(4):e253204. doi: 10.1001/jamanetworkopen.2025.3204 (PMC11976494; doi:10.1001/jamanetworkopen.2025.3204)
Supplement: Supplement 1. — Trial Protocol [file jamanetwopen-e253204-s001.pdf]

RESEARCH STRATEGY

A. Background (Criterion 1): Opioid-treated chronic low back pain has a substantial impact on US society

Chronic non-cancer pain is a leading cause of disability and reduced quality of life in the US, affecting over 100 million Americans and costing nearly $600 billion annually.[1] Existing therapies for chronic pain are suboptimal.[1,2] As a result, 5-8 million patients with chronic pain are treated with opioids and chronic low back pain (CLBP) is the top chronic non-cancer condition for which opioids are prescribed.[3] There are very limited data on the long-term efficacy of opioids, and much concern about their harms, which are *dose-dependent* and include worsened mental health, addiction and overdose death.[4,5] Many opioid-treated patients continue to have inadequate pain relief, and impaired function and quality of life; comorbidities include depression, anxiety and opioid misuse, the severity of which has been linked to worse treatment response in chronic pain.[3,4,6,7] With this complex interplay between CLBP, opioids, co-existing mental health problems, and their effects on outcomes, treatment strategies must address each of these factors. However, there is little research on how to effectively improve outcomes and reduce opioid use; the Institute of Medicine (IOM),[1] the National Institutes of Health (NIH),[8] and the current PCORI's PFA[9] call for studies to identify effective safe strategies for chronic pain care and opioid use reduction (**RQ-1**).

Patients often turn to complementary and integrative health approaches such as mindfulness meditation (MM) to treat chronic pain, including CLBP.[10] MM, a popular mind-body modality, is widely used for improving health and well-being,[11] and may be effective for CLBP.[12,13] Some studies suggest that MM-induced gains can be sustained over time.[14,15] MM training encourages *enhanced awareness* of present-moment experiences (bodily sensations, thoughts, emotions).[16] An intentional non-judgmental awareness and acceptance of one's state of body and mind, without becoming preoccupied by it, promotes *change in the relationship to this experience,*[16] without trying to change the experience itself. Thus, MM fosters an ability to disentangle a given experience (e.g., pain) from associated bodily sensations, emotions and thoughts;[17] this, in turn, is thought to improve emotion regulation, adaptive response to stressors, and a decrease in suffering.[18] MM practice can be a foundation for engagement in life from a place of *"being with," rather than changing*, one's experiences.[19] **MM offers unique skills for acceptance-based pain coping that are different from those taught by cognitive behavioral therapy (CBT; *Fig.1*)**, the "gold standard" of traditional psychological approaches for chronic pain. As opposed to MM, CBT attempts to teach patients to *change* unhealthy illness-related thoughts, emotions and behaviors so that they can develop more adaptive skills for coping with pain and related issues.[20-25] CBT draws upon a range of strategies and is typically tailored to the specific condition; as such, it is usually delivered in separate specialty settings, e.g., pain medicine, mental health or substance abuse programs, in the group or individual therapy format.[20-25] This condition-specific approach also differs that of MM, as "generic" MM curricula, such as Mindfulness-Based Stress Reduction (MBSR),[16] have been successfully applied to a variety of health conditions,[11] suggesting that MM skills are versatile and transferable.

Fig.1. MM and CBT offer different skills for the management of chronic pain.

Mindfulness Meditation (MM)
**Non-judgmental, accepting awareness of present-moment experiences to *change one's relationship* to these experiences**

Cognitive Behavior Therapy (CBT)
**Focus on modifying unhealthy thoughts, emotions and behaviors to *change the experience* and control symptoms**

Gap analysis (RQ-1): **Evidence on MM's efficacy in CLBP, especially in opioid-treated patients, is promising yet limited and inconclusive, presenting a critical knowledge gap**, as noted by the Agency for Healthcare Research and Quality (AHRQ),[11] NIH,[26] PCORI's Stakeholders[27] and systematic reviews.[12,13,28] Assessment of MM for pain has been prioritized by the IOM in the 2nd quartile of its "top 100" comparative effectiveness research topics.[29] The AHRQ's meta-analysis[11] supports MM's efficacy for pain in general (*medium effect* size, Cohen's d=0.33 [95% Confidence Interval, CI: 0.03, 0.62]), while noting the limitations of existing studies, such as inadequate longer-term follow-up and an absence of studies in opioid-treated pain (**RQ-1**). Two more recent trials by our team assessed MM for *opioid-treated* chronic pain.[30,31] Garland et al. (N=115)[31] found that MM, compared to an educational intervention, reduced pain severity (*medium effect size*, d=0.5; p=0.038) by 1 point on a 0-10 scale, and decreased pain interference (*medium effect size*, d=0.78; p=0.003) and the desire for opioids (*medium effect size*, d= 0.5; p=0.027) at 3 months; however, opioid dose was not assessed. **The PI's pilot RCT (N=35)[30] suggested efficacy of MM in opioid-treated CLBP** for reducing pain ratings (*large effect size*, d=0.86; p=0.045) and hyperalgesia (p=0.008) at 6 months, as compared to a wait-list control. Although function appeared to improve as well, this change was not statistically significant (*medium effect size*, d=0.68; p=0.21) in this small trial. The change in opioid dose was also non-significant yet encouraging, with subjects in the MM group reducing dose by an average of 300 morphine-equivalent mg/month relative to controls (p=0.84).[30] The pilot study subjects were satisfied with MM and its effects, viewing it as useful for pain care and opioid use reduction (see B2; *Table 1*).

**Table 1. Examples of the comments about MM's effects for opioid-treated CLBP from subjects in the PI's pilot RCT.**
• *"It helped me learn […] and accept that the pain comes and it will pass," and that "[…] I am not my pain."*<br>• *"It has helped my pain a lot to meditate." "[…] it didn't take away the pain, it just made it easier to deal with it." "It helped me leave somehow into a non-painful state."*<br>• *"[…] learning mindfulness meditation was the best thing I ever did for myself. [It] allowed me to experience reduced pain and, as a result, I was able to reduce my break-through narcotic pain medications in half […]. With reduced pain, I feel I can get around better and feel less depressed and less anxious. Meditation has helped me through anxiety and pain during medical procedures […]. I was also able to avoid increasing my pain medications after I broke my ankle […].I gained energy, peace of mind, contentment; things I had not felt in quite some time. I feel like I have been returning back to myself."*<br>• *"Above all I learned that mindful meditation can help me be more positive about how I deal with my pain."*<br>• *"It helped me with not only my pain […]. I have less pain, […] feel better emotionally. […] my use of opioids has decreased considerably. By me not being in as much pain, my function in my job has increased. I am able to do so much more."*

Although CBT is considered standard-of-care for chronic pain, evidence supports only modest benefit of CBT for CLBP and its long-term effects have not been well studied, especially in opioid-treated groups.[20-22,32,33] A systematic review of behavioral therapies for CLBP[20] noted low quality evidence favoring CBT over no-treatment for short-term pain relief (*small effect size*; pooled weighted mean difference, MD from -7.0 [95%CI: -12.3,-1.7] to -12.7 [95%CI: -20.3,-5.1]) and, to an even lesser extent, for function (MD -2.9 [95%CI:-7.2, 1.4]). The Cochrane Collaboration's meta-analysis[21] found CBT to be better than usual care for short-term pain relief (*small effect size*; MD -5.2 [95%CI: -9.8,-0.6] on a 0-100 visual analog scale), but **not** for longer-term pain relief or function, which was consistent with a second systematic review.[22] The impact of CBT on opioid use in chronic pain is unclear due to a lack of high-quality research on this topic.[20-22,32]

MM and CBT may produce different gains depending on patient's individual characteristics. An RCT[34] of 143 patients with rheumatoid arthritis showed that both MM and CBT improved pain, function and negative affect outcomes. However, those with a history of depression benefitted more from MM than CBT in terms of improved pain coping, and

reduced pain catastrophizing and negative affect ($p<0.05$).[35] The authors hypothesized that MM training, focused on observing and accepting the experience, helped meditators become more adept at detecting subtle early cues, allowing them to start regulating responses to pain or stress *before* emotions become too intense.[34,36] CBT on the other hand involves a cognitive reappraisal, which is usually deployed *after* the emotions have intensified; the usefulness of this cognitive strategy is often compromised when the intensity of negative emotions is high.[37,38] It is possible that individuals with chronic pain and co-occurring anxiety, depression and/or opioid misuse, all conditions common in chronic pain and characterized by high-level of negative affect and emotion dysregulation, may respond better to MM than CBT (**RQ-4**). Evaluation of treatment response in relation to these factors is essential because chronic pain patients with comorbid anxiety, depression or opioid misuse are the very patients who have been found to be less responsive to existing therapies.[39-41] Determining the individual patient phenotype most responsive to particular therapy would help guide clinical decision-making about optimal treatment choice.

As supported by the existing evidence, including data from our pilot RCT, MM has the potential to address both chronic pain and its comorbidities, depression, anxiety, and opioid misuse, and to help patients reduce their reliance on opioids.[11,30,31,42] Even modest dose reduction would be beneficial to patients, decreasing the harms associated with opioid therapy.[5] Research is needed to evaluate the effectiveness of MM for pain severity, function and quality of life, and opioid use, as compared to a suitable alternative treatment such as CBT (**RQ-1, RQ-5**). We have chosen these outcomes because they are endorsed by our stakeholders and are very patient-centered (**RQ-6**). We have designed this study to help inform decision-making by patients with opioid-treated CLBP and their clinicians as they consider choosing between MM and CBT (**RQ-3**); the proposed work will also provide data on the effectiveness of each approach for those with comorbid anxiety, depression and opioid misuse (**RQ-4**). The proposed mixed methods, pragmatic RCT will compare the effectiveness of MM and CBT over 12 months in 766 adults with opioid-treated CLBP. We hypothesize that MM, relative to CBT, will improve patient-centered outcomes: pain and function (Aim 1), quality of life and opioid use (Aim 2), especially in those with anxiety, depression or opioid misuse, factors that can impact treatment response (Aim 3; *Fig.2)*.

Fig. 2. The proposed 12-month RCT: MM, relative to CBT, will improve outcomes in adults with opioid-treated CLBP.

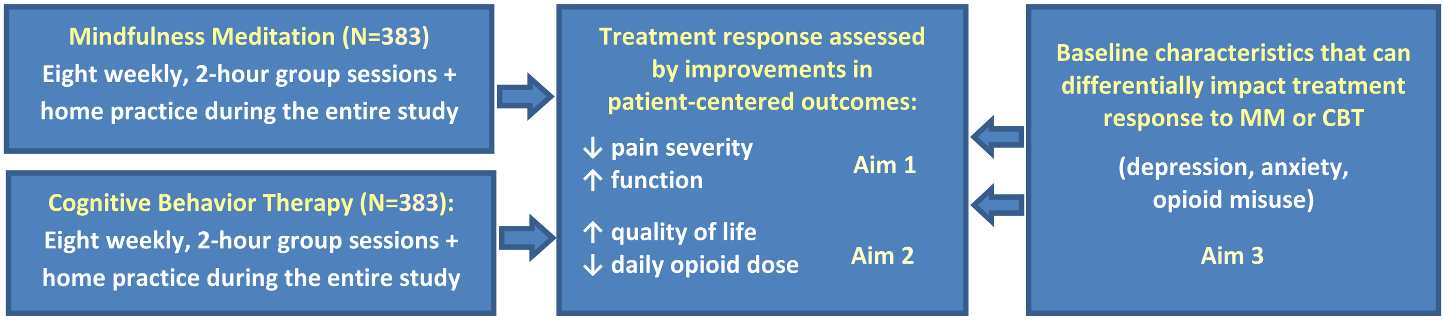

B. Significance: New evidence-based therapies are urgently needed for opioid-treated CLBP

B.1. Our application is patient-centered and addresses the expressed needs of patients with opioid-treated CLBP for safe and effective therapies (*Criterion 4*) The questions addressed by this study are urgent and highly relevant to patients with opioid-treated CLBP, their families, clinicians and the broader US society. In preparation for this study, we solicited input from the vital stakeholders – patients with opioid-treated CLBP. Seventeen patient-participants in our pilot study[30,42] expressed strong dissatisfaction with existing therapy options and were very interested in MM as a therapy for managing their pain, improving function and quality of life, and helping reduce opioid needs. The high patient interest in MM was also reflected in rapid recruitment into our pilot.[30,42] Other important local stakeholders,

including clinicians and health system leaders, have also seen the need and import of this treatment option. Since our pilot RCT, clinicians have continued to inquire about referring patients for MM, and leaders of local health systems have shown enthusiasm for MM as a therapy for opioid-treated patients (see *Letters of Support).* We consulted with patient and clinician advisors about the choice and priority of outcome measures that matter to them and included those into the proposed study (**RQ-6**); these measures include both quantitative and qualitative evaluation, with a patient-initiated videorecording tool. With the existing, documented patient and clinician dissatisfaction with existing therapies and clinical uncertainty about how to improve outcomes in opioid-treated CLBP,[42-47] findings from the proposed study can help guide patients, caregivers, and clinicians about best therapy choices in this patient population (**RQ-3)**. Our Patient Advisors have voiced desire for MM to become available to others with opioid-treated CLBP (see *Letters of Support*):

"[…] as a chronic low back pain sufferer, I am so grateful to work with you […] to find alternative ways to treat chronic pain. I think it is great to use meditation […] to aid in the treatment of chronic pain." "The mindfulness meditation class was a great help to me and I believe it can help others living with this problem."

B.2. This research has the potential to improve healthcare and outcomes in patients with opioid-treated CLBP (*Criterion 2*) The proposed RCT will be the first to directly address the effectiveness of MM compared to CBT in opioid-treated CLBP (**RQ-1, RQ-3**). The study goals and the chosen interventions (**RQ-5**) are very relevant to the affected patients, their clinicians and health systems (see B.1). Outcomes favoring MM would help establish it as standard-of-care and provide the rationale to increase its availability to patients, thus, improving outcomes in opioid-treated CLBP. Offering clinicians effective, safe options for CLBP, such as MM, would help reduce clinician burden related to the management of opioid therapy, as described by **Co-Investigator Jamison**[46] and noted by clinician-partners advising on this project. The mission of health systems is to deliver evidence-based care and improve health of their patients; this mission could be advanced by offering MM, if proven effective, as is noted in the health systems' *Letters of Support*.

The results from our pilot RCT (N=35) suggest that MM is acceptable, feasible and can improve outcomes in patients with high-impact opioid-treated CLBP,[30] documenting the need for a large pragmatic study of MM in this population. At baseline, participants (80% female; 51.8 ± 9.7 years old) reported substantial daily pain (5.8±1.4 points on a 0-10 scale), disability (66.7±11.4 on a 0-100 scale) and high daily opioid dose (148.3±129.2 morphine-equivalent mg/day), confirming subpar results of usual care. By 26 weeks, using *intention-to-treat* repeated measures analysis, the MM group (N=21) lowered pain severity ratings by 1.0 point ([95%CI: 0.2, 1.9]; p=0.045; large effect size, d=0.86; *Fig.3*) and decreased pain sensitivity to thermal stimuli (p=0.008), compared to a wait-list control (N=14). The largest gains (p<0.05) were in MM participants who engaged in "higher-dose" practice, suggesting a dose-response relationship. The MM group subjects, relative to controls, also improved function by 6.5 points ([95%CI: -1.0, 14.0]; p=0.21, medium effect size, d=0.68), achieving the minimal important change (MIC) for both pain[48,49] and function.[48-50] The MM group reported reduction in opioid dose by an average of 10 morphine-equivalent mg/day relative to controls (p=0.82). Patients and clinicians enthusiastically received this study, completing enrollment

Fig.3. At 26 weeks, MM group reduced pain severity ratings compared to controls.

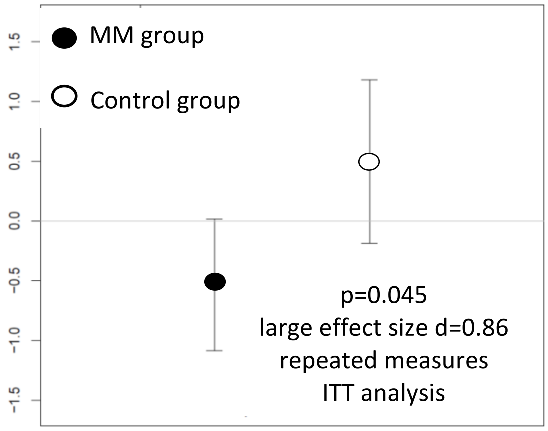

within 10 weeks. During the study, the MM group reported, on average, 164±122 min/week of formal and 104±112 min/week of brief informal MM practice.[30,42] Seventeen MM subjects evaluated the intervention, indicating satisfaction, and rating it, using a 0-10 scale, as "important" (8.0±1.8) and "useful" for pain coping (7.2±2.4), and stating they were likely to continue formal (8.1±2.8) and informal (9.4±1.0) practices; the themes identified by qualitative evaluations (*Table 1*) noted MM as useful for improving pain care and opioid use reduction.[42]

B.3. Findings from this research could be readily disseminated (Criterion 2; PC-4) The MM intervention we are studying can be readily disseminated because it has been manualized by the PI, can be implemented through existing infrastructure, and meets the need of patients, clinicians and health care systems who all expressed strong interest in MM as a therapy for opioid-treated CLBP (see B.2 and Dissemination and Implementation Potential). The fact that the involved institutions (University of Wisconsin, Harvard University, University of Utah) have agreed to pay for the costs of intervention delivery further speaks to the interest and commitment of health systems (see *Letters of Institutional Support*). In addition, the results from the PI's pilot study in opioid-treated CLBP indicate feasibility and acceptability of MM (see B.2).[30,42] **Together, the pilot study provides support for MM as an effective, feasible and acceptable modality**, all essential predictors of successful dissemination. Conclusive supportive evidence on the effectiveness of MM, such as the proposed study can provide, high interest in this modality, and strong assembled coalition of partners, will help add MM to standard-of-care therapy, making it accessible to patients with opioid-treated CLBP.

C. Patient Population

C.1. Study Population (PC-2) Through the proposed recruitment plan (see D.6.a) we will enroll English-speaking adults with opioid-treated CLBP who are diverse in terms of gender, race, ethnicity, and mental and physical health conditions. Our sample will enroll participants through three sites: Madison, Wisconsin, Boston, Massachusetts, and Salt Lake City, Utah. The Madison metropolitan area includes nearly 0.5 million people. The Madison site will have access to patients from rural, suburban and urban communities care for in the collaborating 5 health systems (see C.2.a). The Boston metropolitan area includes 4.5 million people and is an urban region with good representation of racial minorities (greater Boston is approximately 45% non-white). The Boston site will be able to draw patients from two hospitals and affiliated clinics of the Partners Healthcare System: Massachusetts General Hospital and Brigham and Women's Hospital. The Salt Lake City metropolitan area has a population of 1.2 million, of which, in 2010, 75% was white, 22% Hispanic/Latino and 3% African American. The Utah site will draw patients from all 11 University of Utah Health Care primary care clinics across the greater Salt Lake City metropolitan area and the clinics associated with the University of Utah Hospital. Based on the demographic information about our sites and their prior enrollment numbers, we estimate that at least 51% of our study population will be female, and 13% will identify as African American, 11% as Hispanic, with the remainder primarily non-Hispanic white. Because our study participants as a group will be predominantly urban and English-speaking (necessitated by the nature of the intervention), our results may not be generalizable to those in rural areas or among patients who are not fluent in English.

Opioid-treated CLBP patients meet the definition of hard-to-reach population. Typically, they suffer from multiple chronic conditions (especially anxiety, depression, opioid misuse); have special healthcare needs due to disability; and are of low-income. In our pilot study (see B.2), 66% of our 35 subjects were near or below the poverty line, and all suffered from severe CLBP-related disability.[30,51] We will implement the strategies[52] we successfully employed to recruit and retain under-represented and hard-to-reach groups in our pilot studies, including tailoring our study procedures to

account for participants' physical disability. Unlike most prior chronic pain trials, which precluded recruitment of opioid-treated patients with "multi-morbidity," we will apply pragmatic eligibility criteria to enhance generalizability to a real-life population of patients with opioid-treated CLBP. We will not exclude based on anxiety, depression or substance use disorders, as these conditions are common and can impact treatment success in opioid-treated CLBP,[4] or based on a socio-economic status, gender, race or ethnicity. **Our preliminary work[53] corroborates high co-occurrence of mental health problems and opioid-treated CLBP** (*Fig. 4)*. Analysis of electronic health record (EHR) data from approximately 250,000 adults evaluated in the UW Health clinics in 2015 showed that 4,977 met criteria for CLBP and were treated with opioids. In this group, the prevalence of depression, anxiety and alcohol/drug use disorders, and the number of clinic visits were high, and increased in relation to opioid dose. We will assess the severity of these expected comorbidities and their impact on the effect of our interventions. Overall, diversity in demographics and comorbidities will facilitate an exploration of the heterogeneity of treatment effects (**HT-1**).

C.2. Recruitment Settings We will recruit participants from primary care and specialty clinics; the community in Madison, WI; Boston, MA; and Salt Lake City, UT; and, for the all-virtual study option, from across the US (see D.6.a. for recruitment strategies). Based on our prior experience and stakeholder feedback, we anticipate great interest in the study that will facilitate efficient recruitment (see B.1 and *Letters of Support* from patients, clinicians, patient and community advocates, and leaders of the collaborating health systems).

Fig.4. Adults with opioid-treated CLBP (N=4,977): worsened clinical profile (% depression, anxiety, alcohol/drug use disorders; number of clinic visits) across the spectrum of daily morphine-equivalent opioid dose.

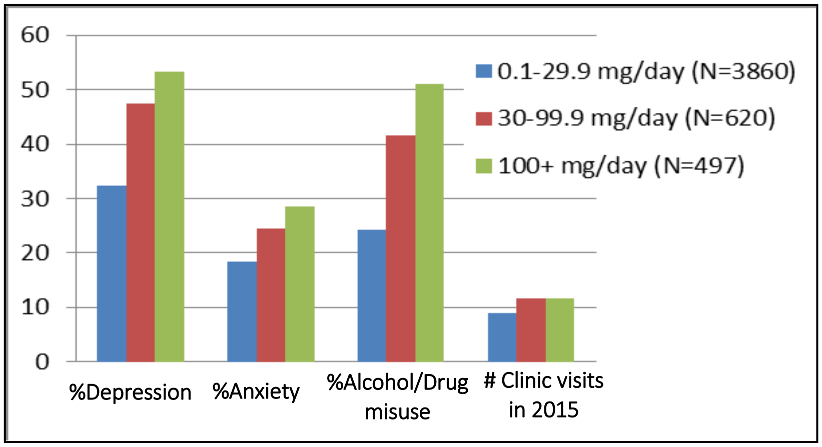

C.2.a. Recruitment Infrastructure The collaboration between multiple health systems in Madison, Boston, and Salt Lake City will offer a diverse and robust recruitment base (*Table 2*) and increase generalizability of our findings. **UW Health** is a large academic health system with a strong primary care base of 360,000 patients, from uninsured to those privately insured. **Access Community Health Centers** is a Federally Qualified Health Center with over 13,000 adult patients, majority of whom are uninsured or have public insurance. **Group Health Cooperative**, a non-profit HMO, serves 79,000 members, **UnityPoint Health-Meriter** serves nearly 90,000 patients, and **SSM Health** serves close to 300,000 adults with private or public insurance. The **Pain Management Center of the Brigham and Women's Hospital**, our primary recruitment base in Boston, serves 20,000 pain patients, and its "parent" organization, **Partners Healthcare System**, sees 1.65 million patients annually. The **University of Utah primary care clinics** consist of 11 community health clinics, which serve over 120,000 patients across the greater Salt Lake City metropolitan area, and its "parent" organization, **University of Utah Health Care,** is comprised of 9 hospitals and over 80 clinics, with over 1 million outpatient visits per year. **We estimate that approximately 19,000 patients with CLBP are treated with opioids across these sites**. All sites use the same EHR software (EPIC), enabling data extraction for recruitment and on prescribed opioids. UW Health **data analyst** will enable data extraction from the Clinical Data

Warehouse, which is a repository of EHR data for UW Health and Access systems. The Research Patient Data Registry provides a centralized EHR data warehouse available to the research team in Boston; the Electronic Data Warehouse at University of Utah Health Care provides EHR data access to the research team in Salt Lake City.

**Table 2. The collaborating health systems will provide a robust infrastructure and support for effective recruitment.**
**Madison**-based major health systems: Estimated annual number of adult patients who are potential study participants<br>● University of Wisconsin (UW) Health: 4,977 adults with opioid-treated CLBP (estimated from the EHR data)<br>● Group Health Cooperative: 660 adults with opioid-treated CLBP* (1,200 with opioid-treated chronic pain)<br>● SSM Health Plan: 4,100 adults with opioid-treated CLBP (estimates are extrapolated from the UW Health EHR data)<br>● UnityPoint Health - Meriter: 1,200 adults with opioid-treated CLBP (estimates are extrapolated from the UW Health EHR data)<br>● Access Community Health Centers: 181 adults with opioid-treated CLBP* (329 with opioid-treated chronic pain)
**Boston**-based health system: estimated annual number of adult patients who are potential study participants<br>● Pain Management Center, Brigham & Women's Hospital: 3,400 adults with opioid-treated CLBP (estimated from the EHR data)
**Salt Lake City**-based health system: estimated annual number of adult patients who are potential study participants<br>● University of Utah Health Care primary care clinics: 4,500 adults with opioid-treated CLBP (estimated from EHR data)

*Conservative estimates, derived from literature, indicate that at least 55% of patients with opioid-treated chronic pain have CLBP.[3,31]

C.2.b. Recruitment Targets Given our recruitment experience in RCTs of MM and other behavioral interventions, and with patients with opioid-treated CLBP, chronic pain, and substance use disorders, **we conservatively estimate the need to screen 3,830 individuals to enroll 766 participants** (*Table 3*) and **that the attrition rate will not exceed 20%**. In our pilot RCT of MM for opioid-treated CLBP, 304 potential subjects were identified through the EHR data and clinician and self-referrals; 87 were screened, 48 were eligible and 35 were enrolled.[30] During the 26-week pilot study, no participant withdrew, and two (5.7%) missed the final assessment. In the PI's other trial of MM, 92% of 123 alcohol dependent adults completed a 1-year follow-up.[54] Our other studies have also had excellent recruitment and retention. Two RCTs of MM led by **Bruce Barrett** had an overall 95% (540/567) retention rate over a 9 month follow-up. For these studies, 2,080 adults were screened, 183 were found eligible but declined and 567 (27.3%) were enrolled.[55,56] In **Co-Investigator Jamison**'s study of a behavioral therapy for opioid-treated chronic pain, 58/62 (94%) completed the 6-month study.[57] We will strive to achieve our goals for women and minority participation (*Table 4*), using strategies described in D.6.a.

**Table 3. Recruitment Plan**	***Madison, WI***	***Boston, MA***	***Salt Lake, UT***	***Total (N)***
Total number of participants expected to be screened	1,275	1,280	1,275	**3,830**
Of those screened, total number of participants expected to be eligible	459	461	459	**1,379**
Target sample size	255	256	255	**766**

**Table 4. Estimated Racial/Ethnic & Gender Enrollment**	**Madison, WI**		**Boston, MA**		**Salt Lake, UT**		**Total N = 766**
**Race**	**Male (N)**	**Female (N)**	**Male (N)**	**Female (N)**	**Male (N)**	**Female (N)**	
American Indian/Alaska Native	0	2	1	4	2	3	**12**

Asian	6	7	12	8	5	7	**45**
Black/African American	6	7	27	23	18	19	**100**
Hawaiian/Pacific Islander	0	1	0	0	2	3	**6**
White	107	112	84	78	93	98	**572**
Multirace	3	4	9	10	2	3	**31**
**Ethnicity**	**Male (N)**	**Female (N)**	**Male (N)**	**Female (N)**	**Male (N)**	**Female (N)**	**Total (N)**
Hispanic (Latino/Latina)	6	8	24	22	8	15	**83**
Non-Hispanic	116	125	110	100	114	118	**683**

C.2.c. Barriers to recruitment, ways to overcome them We have experienced teams in all study sites whose members have a track record of working well together, and of successful recruitment and retention in RCTs of psychotherapies for chronic conditions, including pain. **These strong teams, along with the forged collaborations with partners who have a vital interest in this research, will help us overcome potential barriers** to recruitment and effective study conduct. The health systems are engaged and supportive (see *Letters of Support*). Both the leaders and the clinicians within these systems note the need for effective treatments that are acceptable to patients and view MM as having a strong potential to serve this role. All involved health systems have implemented some initiatives to optimize opioid prescribing. Our study is timely and well-aligned with their institutional goals, yet it will not interfere with the clinical work and existing projects. The study interventions will be provided free-of-charge. Should we have difficulty recruiting at one site, we will have the PI at that site observe and review recruitment procedures to identify problems, and bring them back to the stakeholders and investigative team to devise a solution. We have carefully designed the advisory roles for our Patient/Family Advisors and a Study Advisory Committee (SAC) to help problem-solve around potential recruitment and retention challenges (see E). The Wisconsin Network for Research Support (WINRS), led by **Betty Kaiser** PhD RN and **Gay Thomas** MA (see *Letter of Support*) will provide services to optimize strategies for recruitment, engagement and retention (see E). The **Community Advisors on Research Design and Strategies** (**CARDS®**), a group of diverse community members, will review recruitment plans and materials. WINRS staff will develop and deliver a tailored training for our Patient/Family Advisors and provide ongoing support for successful patient stakeholder engagement throughout this grant. **Penney Cowan**, leader of a national organization of persons with chronic pain, and **Cheryl Wittke**, director of the local community coalition and advocacy program for improving community health (see E), will also help promote recruitment, problem-solve around issues of retention and assist in dissemination of results through their member networks and respective websites (see *Letters of Support*). **Elizabeth Jacobs** and **Linda Oakley** have expertise in engaging hard-to-reach populations and will assist with recruitment, retention and engagement. The recruitment across the US for the all-virtual option of the study will be completed in collaboration with the investigator and stakeholder networks, and national advertising, e.g., through ClinicalTrials.gov or Facebook sites.

D. Study Design or Approach

D.1. Specific Aims (Criterion 3) Our objective is to compare the effectiveness of MM to standard-of-care CBT for improving outcomes in patients with opioid-treated CLBP. To achieve this goal, we will conduct a multi-site, mixed-methods, pragmatic RCT in adults with opioid-treated CLBP. We will follow participants over 12 months and compare quantitative (survey, EHR) and qualitative outcomes that matter to patients and their families in 383 adults randomized to the MM group to 383 adults randomized to the CBT group, controlling for relevant factors. **Our specific aims are:**

AIM 1. To compare the effectiveness of MM to CBT for reducing pain and increasing function (co-primary outcomes) in adults with opioid-treated CLBP. H1: Participants in the MM group will report a greater reduction in pain severity and a greater increase in function at 6 and 12 months compared to participants in the CBT group.

AIM 2. To compare the effectiveness of MM to CBT for improving quality of life (QoL) and reducing daily opioid dose (secondary outcomes) in adults with opioid-treated CLBP. H2: Participants in the MM group will report a greater improvement in QoL and a greater decrease in daily opioid dose at 6 and 12 months compared to participants in the CBT group.

AIM 3. To examine if participant baseline characteristics impact treatment response to MM or CBT. H3: Among those with increased baseline symptom severity of negative affect (depression, anxiety) and opioid misuse behaviors, MM will be more beneficial than CBT for improving primary and secondary outcomes.

D.2. The proposed research has been stakeholder driven from its inception (RQ-6) As primary care providers, pain medicine and psychiatry physicians, psychologists, physical therapist and clinical social workers, the investigators have experienced the challenges and frustration associated with the lack of safe, effective treatments for opioid-treated CLBP, and limited ability to help patients who use opioids for their CLBP. Both clinician and patient advisors for this project noted that traditional behavioral therapy such as CBT, while helpful, may be unfeasible for many patients due to the shortage of providers and lack of health insurance coverage, or may be unacceptable to them due to its association with stigmatized mental health problems. MM may have higher acceptability and is popular among patients with chronic pain.[10] The study design, methods, and outcome measures were informed by patient stakeholders, their family members, patient advocates, clinicians, leaders of the health systems, and the **Community Advisors on Research Design and Strategies.®** In fact a suggestion from a stakeholder led to the inclusion of the **video-recording tool**, an innovative, patient-centered qualitative component of the study (see D.7). The **patient-advisors also recommended a two-arm study design** (MM vs CBT), without including a wait-list “inactive” control group (see E).

D.3. Choice of Study Design Following the guidelines on the design of pragmatic clinical trials in chronic pain[58-62] and our stakeholder input, we propose a 5-year multi-site RCT (N=766), comparing the effectiveness of MM and CBT for improving patient-centered outcomes over 12 months in adults with opioid-treated CLBP. Existing evidence and pilot data guided the sample size estimates. Patient-reported outcomes (PROs) of pain severity and function will serve as primary, and quality of life and opioid dose as secondary measures of effectiveness; these PROs are recommended by guidelines,[60-62] and have been endorsed by our stakeholders (see D.7.a). Collection of PROs on the factors hypothesized to impact treatment response will help discern for which patients MM vs. CBT may work best (Aim 3). Qualitative ancillary data will complement our main measures. To minimize participant burden, we will collect only the essential PROs, primarily via web (see D.6.c), as suggested by our patient advisors. The proposed goals are feasible within the project duration (see *Milestones*), enabled by use of multiple sites and robust, engaged recruitment base (see C.2). We have chosen a 12-month follow-up because it is recommended for effectiveness testing[9,61] and is feasible.

D.3.a. The strengths of our design include a multi-community study with pragmatic eligibility criteria (see D.4) facilitating generalizability; testing of an established MM modality that has already shown feasibility, acceptability and reach in the target population (see B.2) and in many communities across the US; a well-defined standard-of-care CBT comparator intervention; PROs selected according to the recommendations (IMMPACT, NIH and National Patient-Centered Clinical Research Network, PCORnet) for clinical trials in chronic pain and CLBP,[8,31,60-63] and endorsed by our

stakeholders; and an experienced team of researchers and engaged stakeholders to successfully carry out the study. We have carefully designed our study to minimize potential sources of bias (**CI 1,2,3,4**) that may affect the internal and external validity of clinical trials (**IR-6**),[64] as described in *Table 5.* In addition to strategies outlined in Table 5, we will also use a written **study protocol** (**RQ-2**) to standardize the procedures and a plan for a rigorous dissemination of results (*Table 5*), following the CONSORT reporting guidelines (**IR-6**) to avoid reporting bias.[65]

Table 5. Potential sources of bias and methods and approaches that we will implement to minimize their impact.

Bias Type	**Approach to Minimizing Bias**
Selection bias	Broad eligibility criteria (see D.4) and standardized definition of the target population
Allocation bias	RCT design with allocation concealment
Ascertainment bias	Blinding of outcome assessors, statisticians and investigators to the group status until after data collection, and primary outcome analyses and reporting are completed
Information/recall bias	Vetting of the study materials by patient/family/community advisors; validated patient-reported measures for effectiveness testing, corroborated by qualitative assessment of pain, function and QoL (videorecording tool) and EHR data on opioid dose; structured, systematic data collection
Intervention/comparator bias	Framing the interventions in an expectancy-neutral manner; similar structure, therapist/group contact time in both interventions; strategies to ensure treatment fidelity; same frequency of contacting MM and CBT participants by the study team; blinding of outcome assessors to the group status
Drop out & dissemination biases	*A priori* protocol for handling of the missing data, outcome analysis (i.e., intention to treat; sensitivity analysis); prospective registration of our trial; adherence to guidelines for outcome reporting in RCTs

D.4. Study Population (RQ-3) We will use a well-defined, pragmatic approach to include participants while minimizing the exclusionary criteria to ensure we have a diverse sample from which our findings can be generalized. **Eligible participants** will be English-speaking adults ≥ 21 years old, with CLBP (defined as a pain in lumbosacral region or sciatica for ≥3 months) as the main pain source; who report an average daily pain score ≥3 on a 0-10 numerical rating scale and are treated with ≥15 mg/day of morphine-equivalent dose for ≥3 months, and who report at least moderate CLBP-related disability (≥21 score on the Oswestry Disability Index).[66] **We will exclude** individuals who report: prior formal MM or CBT training; current pregnancy, borderline personality, delusional, bipolar (mania) disorders ("active" in the prior 12 months); and inability to safely or reliably participate in the study. **Rationale:** CLBP is the leading chronic non-cancer pain for which opioids are prescribed. Age 21 years old defines an adult per the NIH guidelines, and treatment with long-term opioids for CLBP is uncommon in those younger than 21 years.[53] Because pain severity and function are the main outcomes, we will enroll those reporting at least moderate pain/disability. We will enroll patients treated with ≥15 mg/day of morphine-equivalent dose. We chose this daily dose threshold for several reasons. a) This approach will enable evaluation of dose change (including any potential increases) across the range of daily opioid dose categories. This is important, as the risk associated with opioid therapy is dose dependent. Even those in a lower daily dose category of 20-49 mg/day have a 44% increase in the risk of overdose compared to those treated with a daily opioid dose of less than 20 mg/day (Hazard Ratio 1.44; 95% CI: 0.57–3.62).[67] In addition, the group of patients treated with lower daily opioid doses may have the best chance for tapering off opioids completely, should other treatments (e.g., MM) prove effective. b) The enrollment of patients treated with a broad spectrum of daily opioid doses will facilitate evaluation of the study intervention effects on opioid use/dose over time, especially in the proposed subgroups of participants as those treated with a lower daily opioid dose are more likely to have lower negative affect and opioid misuse scores

compared to those treated with higher daily opioid doses. We will exclude those with specific active mental health disorders, described in the exclusion criteria, and other conditions or circumstances that may pose concerns for safety or reliable participation. We will not exclude on the basis of common mental health (anxiety, depression) or substance use disorders, because they are prevalent in this population and can affect outcomes (Aim 3 hypothesis). Trained personnel, following a written protocol (**RQ-2**), will conduct eligibility screening virtually or in person. We will work with stakeholders to optimize recruitment and retention methods especially for hard-to-reach groups (see C.2.c).[52] Our patient-advisor who is an African American and a former participant in the pilot study[36] will provide advice on how to recruit and retain minority participants in the study.

D.5. MM and CBT Interventions In this RCT, adults with opioid-treated CLBP will be randomly assigned to either receive the MM intervention (MM arm; N=383) or to receive the CBT intervention (CBT arm; N=383). The interventions will be provided in addition to the care participants are already receiving through their regular providers. The choice of CBT as a comparator intervention is appropriate (**RQ-5**) because it is standard-of-care for CLBP,[1,20-22,32] and, similarly to MM, is a psychological therapy that can be delivered in group format.[23,24]

D.5.a. Similar Structure of the Interventions Both interventions have been patterned after existing programs[16,23,24,68,69] and adapted to meet the needs of patients with opioid-treated CLBP, with input from content experts and patient advisors who were participants in the PI's pilot RCT.[36] The structure of these interventions will be matched in terms of duration, setting and contact time to limit *intervention bias* (*Table 5*). Each intervention will follow a written manual and be delivered by a trained therapist over 8 weeks (weekly two-hour group sessions). In addition, subjects will be asked to practice MM or CBT strategies at home for at least 180 min/week during the study and log their practice minutes. The session format will be comparable between the interventions and delivery formats (see the outline of MM and CBT sessions in Appendix A). Each session will start with the review of home practice, experiences, concerns and questions, followed by a MM or CBT exercise, then introduction to the session-specific core concepts and 2-3 MM or CBT exercises (concept application), each followed by discussion of subject experiences and MM skills for coping with challenges related to opioid-treated CLBP. This interactive format will enable monitoring and enhancement of subject treatment receipt and enactment, essential elements of *treatment fidelity* (see D.5.e). Each session will end with a review of the home practice for the following week. These methods and targets are feasible.[30,42,54,70] Taking into account a set-up of the MBSR and group CBT courses in clinical settings, each MM/CBT group will typically consist of 7-20 participants per group.[23,24,71,72] The intervention sessions for a given intervention cycle will be delivered in person or virtually.

***D.5.c. The "Mindfulness Meditation for Chronic Pain"* MM intervention was patterned after established curricula and tailored to meet the psychophysical needs of patients with opioid-treated CLBP. The PI** and Janice Singles and Shilagh Mirgain, pain psychologists who will deliver the proposed study interventions in Madison, were responsible for tailoring. Drs. Singles and Mirgain each has 20+ years of personal practice and 10+ years of applying MM as a therapy for chronic pain. The MM intervention has evolved from the well-accepted MBSR, developed nearly four decades ago by Jon Kabat-Zinn as an integrated treatment package for stress and chronic pain,[16,73-75] and the programs that grew from the MBSR: the Mindfulness-Based Cognitive Therapy (MBCT), which targets mood disorders, and the Mindfulness Based Relapse Prevention (MBRP), which targets addictive disorders.[11,68,69] The MM intervention was tested by the PI in a 26-week pilot RCT,[30,42] and found to be effective and well-received (see B.2). The pilot study experience has underscored the importance of tailoring the intervention to the needs of this clinical group; the "mindful movement" components required extensive adaptation to accommodate the participants' severe physical impairment.[30,42] Based on the pilot

experience, the **MM intervention will be further refined by the PI and Dr. Garland, with input from content expert Dr. Richard Davidson**, and from stakeholders prior to finalizing the manual. The key components of the MM intervention[30,42] include formal, longer meditation practices and brief techniques for practicing MM skills throughout the day and as-needed for pain /stress coping (see *Appendix A*), as to infuse mindful awareness into daily life. The MM training will encourage subjects to apply MM skills prior to deciding whether to take an opioid medication for break-through pain, with the idea that a mindful pause will serve as an alternative to opioids, therefore, decreasing reliance on opioids. This approach is supported by a recent RCT of Dr. Garland-developed MM-based intervention, a "Mindfulness-Oriented Recovery Enhancement," which targeted opioid misuse behaviors in patients with opioid-treated chronic pain; in this study, the MORE intervention, compared to an educational comparator intervention, resulted in decreased pain severity and reduced ratings of the desire for opioids.[31]

D.5.d. The CBT Intervention **will be adapted from two existing programs**: "Managing Chronic Pain: A Cognitive-Behavioral Therapy Approach"[23,24] and "Mastering Chronic Pain: A Professional's Guide to Behavioral Treatment"[25] (**authored by Co-Investigator Jamison**), under the guidance of **Robert Edwards and Robert Jamison**, pain psychologists with extensive expertise in CBT for chronic pain. The CBT intervention format (see *Appendix A)* will match that of MM. CBT training includes a variety of cognitive and behavioral strategies, such as cognitive restructuring of maladaptive pain-related beliefs, coping skills and problem-solving training, psychoeducation about the scope and impact of the pain syndrome, progressive muscle relaxation and strategies for behavioral activation, including pacing, activity scheduling or motivating physical activity.[23,24] These strategies lay a foundation for learning to modify, diminish or re-frame negative thoughts and beliefs about pain. **None of the CBT techniques teaches acceptance and non-judgmental approach to the pain experience as MM does.** The ABC model[76] exemplifies the CBT approach that can be applied to every-day challenges. In this model, ***A*** stands for the "activating event" (e.g., worsened pain); ***B*** stands for the "belief system" (i.e., the unhealthy pattern of thoughts, attitudes and beliefs about pain); and ***C*** is for the "consequences" of the event (e.g., negative emotions or avoidance behaviors arising from the maladaptive belief system about pain). Following the ABC Model steps, individuals are asked to work through examples from their life experiences, especially in relation to pain, to generate insight into the manner in which their beliefs can shape responses to a given situation and serve as basis to change the unhealthy ABC patterns.

D.5.e. Treatment Fidelity of the Study Interventions Monitoring and enhancing fidelity of behavioral interventions is crucial for preserving the validity of conclusions and for future dissemination.[77,78] We will draw upon our previously tested methods[30,42,54] and the NIH Behavior Change Consortium-issued guidelines[77,78] to ensure integrity of intervention delivery. We will use written protocols for therapist selection and training and for intervention delivery; such protocols, including the intervention manual, were used in our prior RCTs[30,42,54] and will be refined for the proposed study. The training of therapists will include an intensive in-person workshop; therapists will demonstrate competence and adherence before guiding an intervention. Competence and adherence will be measured by the existing *Adherence & Competence Scale* (ACS),[79] adapted to this study[30] by the Investigators. To maximize consistency and minimize "contamination" of intervention delivery, one therapist per site will deliver MM and another will deliver CBT. Trained back-up therapists will be available. A trained researcher will be available at each intervention session to assist the therapist, track attendance, collect participant home practice diaries, and score the therapist adherence and competence after each session, using the above ACS scale. We will audiotape the intervention sessions and audit randomly-selected sessions, using the ACS scoring system, as we did in our prior studies.[30,42,54] Variations in intervention

delivery ("therapist drift"), detected by the ACS scores, will be discussed by the study team, with feedback offered to the therapist to ensure treatment fidelity and limit bias (*Table 5*). We will monitor subject treatment receipt and enactment ("skill acquisition;" see D.5.a). We will minimize the impact of non-protocol treatments by excluding potential subjects with prior MM/CBT training and regularly inquiring as to whether or not participants have received non-protocol MM or CBT during the study, tracking its receipt and assessing it as a covariate. **Drs. Zgierska, Garland** and **Nakamura** will be responsible for ensuring treatment fidelity of the MM intervention, and **Drs. Edwards and Jamison** will be responsible for ensuring treatment fidelity of the CBT intervention. The PI was trained in the delivery of MBRP,[68] a similar intervention to that proposed, at the University of Washington in Seattle by Drs. Sarah Bowen and late Alan G. Marlatt, then led per-protocol therapist trainings for her prior RCTs and co-facilitated MM sessions in these trials.[30,42,54] Drs. Edwards and Jamison are experienced psychologists and clinical trialists.[39-41,57] We will ensure treatment fidelity by implementing the strategies described above: a protocol-driven approach to therapist selection and training; audit, evaluation and feedback on the therapist's performance; and assessment of skill acquisition by participants.

D.6. Study Procedures Will Be Standardized A written study protocol (**RQ-2**) will be developed to standardize the approach to study tasks, procedures, handling of protocol violations, data management and analysis, including the approach to missing data, and result reporting. Our prior NIH-funded RCTs followed detailed, written protocols,[30,55,56] which will be adapted to the proposed study. Although participants and therapists cannot be blinded to the study intervention, investigators, outcome assessors and analysts will be blinded to the group status until the first stages of analysis and reporting are completed. Subjects will be asked not to reveal their group status. Procedures for data management (**IR-2**) will be standardized to promote high-quality data and data sharing (see *Data Sharing* section).

D.6.a. Recruitment Strategies Although we will strive to standardize all procedures, this will be balanced against the preferences of each health system so that the recruitment plan is feasible and acceptable to each system. We will use previously-tested methods:[30,54-56] **1)** *EHR-based recruitment*: The UW Health data analyst **and the PI** will develop, using published criteria,[80] the protocol to identify appropriate patients with opioid-treated CLBP. They will work with each health system on a data extraction protocol while also adapting it to meet the needs of each system (see *Letters of Support*). **Because all collaborating health systems use the same software (EPIC Systems Corporation), the recruitment based on EHR data is feasible**. Identified potential participants will be mailed an invitation letter along with a response card. Those who do not opt-out will be called by the study coordinator. This method worked well for the UW Health and Access systems in our pilot RCT in patients with opioid-treated CLBP.[30] **2)** *Direct referral by clinicians:* We will also encourage clinicians to refer appropriate patients, as was successful at SSM Health, GHC and UnityPoint Health in the pilot study[30] and in the PI's RCT of MM for alcohol dependence.[54] The clinician-investigators have worked with all health systems in clinical settings and will facilitate recruitment. **3)** *Patient self-referral*: We will place the study information on a website developed for the study, brochures in clinics, community centers and other locations advised by stakeholders, and will consider placing ads (e.g., in local newspapers), which were successful in **Bruce Barrett**'s two RCTs of MM.[55,56] Our patient and community advisors, staff from the Wisconsin Network for Research Support and Investigators **Jacobs and Oakley** who have expertise in engaging patients, especially hard-to-reach populations, will advise on the recruitment strategies and materials. To screen for eligibility, the study coordinator will call potential participants; patients may also call the study directly. During the initial contact (*Table 6*), the coordinator will explain the study, answer questions, then, after their verbal consent, will screen potential participants for eligibility. To ensure consistency, the materials describing the study and used for eligibility screening will be standardized, similar to our prior trials.[30,54-56]

D.6.b. Enrollment, Baseline assessment Eligible, interested adults will be scheduled for a virtual or in-person meeting with the study coordinator to complete enrollment, baseline assessment and randomization (*Table 6*). All baseline data will be collected prior to randomization.

D.6.c. Randomization Subjects will be randomized equally between MM and CBT arms, using a permuted blocks of random size strategy, stratified by site and prepared by the study statistician. Sealed envelopes with group assignment will be distributed consecutively; the seal will be broken after baseline assessments are complete (see D.6.b).

Table 6: Timeline of Study Activities, Contact and Data Collected for Individual Participants During the 12-month Study

Recruitment & screening	Enrollment, Baseline Assessment, Randomization	Study Intervention	Follow-up Assessments at 3, 6 and 9 months	Exit Assessment at 12 months post-entry
▪Recruitment, eligibility screen (virtually or in person)	▪Informed consent;<br>▪All survey-based data (virtually or in person)<br>▪Brief videorecording (virtually or in person)<br>▪Randomization<br>▪EHR: prescribed opioids (researcher collected)	▪MM/CBT course (virtually or in person; 8 weeks, 2 hours/week);<br>▪MM/CBT practice (at home; entire study, 30 min/day, 6 days/week)	▪Survey-based data (virtually):<br>outcome, adherence, safety data at 3, 6, and 9 months<br>other survey data at 6 months only	▪All survey-based data (virtually or in person);<br>▪Brief videorecording (virtually or in person);<br>▪EHR: prescribed opioids (researcher collected)<br>▪**Subgroup**: in-depth interview (by phone)

D.6.c. Follow-up Assessments Following the advice from our stakeholders, former participants in the PI's pilot study,[30] the majority of follow-up data will be collected virtually. When available, the exit follow-up will be conducted in person. Follow-up data on main outcomes will be collected at 3, 6 and 9 months; other survey-based data will be collected at 6 and 12 months post-entry. To minimize recall bias, subjects will be asked to keep track of MM and CBT practice and any adverse effects, and submit it by internet, phone or mail. We will design the study's RedCap database to send participants several email reminders, and they will receive reminders by phone/text/email, regarding completion of their follow-up surveys if needed. Those unable or unwilling to use the online reporting / reminders will be contacted through other means. During the exit assessment, we will collect all survey-based data and brief video-recorded interviews. Among those who completed 12 months of data collection and reduced their opioid dose, we will identify a **subgroup** of up to 30 participants/arm (60 in total) to complete in-depth qualitative interviews by phone.

D.6.d. Retention, adherence Every effort will be made to retain participants through the 12-month study period, encourage completion of all study activities and measures, and **minimize missing data**. Each subject will be assigned a study representative to develop a personal rapport, and enhance completion of study measures and retention.[55,56] To meet these goals, we will use brief assessment tools (seeD.7), limit the number of assessments and in-person meetings, allow breaks during study activities, and reimburse participants for their time/effort. They will be compensated for study participation: $30 for each baseline and exit survey completion, and $20 for each follow-up survey assessment; $20 for each videorecording; $50 for an in-depth interview (subgroup of participants); and $10 for each attended intervention session. We will also incentivize adherence by implementing a $100 bonus for participants adherent to the intervention sessions during a given MM or CBT intervention cycle. The proposed methods are feasible and have been successfully used in our prior RCTs.[30,54-56] The medical or research facilities for the in-person (when available) intervention delivery and assessment meetings will be easily accessible by bus and/or have convenient parking. We will collect information on

different ways for contacting participants (home / email addresses, phone numbers, emergency contact) and update it at each follow-up contact.

D.7. Patient-Reported Outcomes That Matter To Patients and Families (RQ-6; PC-3; IR-1,2,4) To meet our first two aims we will compare pain severity and function (Aim 1), quality of life and opioid dose (Aim 2) between the MM and CBT participants over the 12 month study period (D.7.a). To address Aim 3, we will collect data on factors that might impact or explain our outcomes (D.7.b). Outcome data will be collected using validated patient-reported outcome measures endorsed by guidelines[60-62] and by our stakeholders (**IR-1,4; PC-3**). To expand understanding our results, we will collect qualitative data (D.7.c). To minimize burden, only baseline and exit data will be collected in person (*Table 6;* D.6.c), and we will use the NIH-endorsed "minimum data set"[61] to maximize the efficiency of data collection (*Tables 7*). The **proposed survey measures, including the time needed to complete them (*Table 7*), were feasible and acceptable** in our prior studies.[30,39-41,57] Main outcomes measures (D.7.a), collected at all time points, are presented in *Appendix B.*

D.7.a. Main Outcome Measures To prepare for this study, we discussed with our stakeholders outcomes that matter to them (**PC-1**). The PI held a focus group of patients with opioid-treated CLBP (N=19)[30,42] and queried individual family members (N=9) and clinicians (N=24). All groups identified reducing pain and disability as the top priority, and improving quality of life and reducing opioid dose as important secondary goals. This is consistent with existing broader literature, which indicates that opioid-treated patients do not favor strategies that solely focus on opioid dose reduction.[47]

Pain intensity will be assessed using the 0-10 point *Numerical Rating Scale* (NRS) from the Brief Pain Inventory (BPI).[81,82] The NRS is reliable and valid in CLBP (α=0.85).[83] A 1-point between-group difference is considered a minimal important change (MIC);[48,49] this MIC was reached at 6 months (p=0.045) in our pilot RCT.[30] Physical function will be measured using the validated *Oswestry Disability Index* (ODI),[66,84] which has good internal consistency (0.76-0.90), is reliable (0.83-0.99) and responsive to change.[60,85] Its total score (0-100) reflects the percent of disability. A reduction of 4-10 points constitutes the MIC.[48-50] The between-group difference reached 6.5 points (p=0.21) in our pilot study.[30] In addition, functional status will also be assessed with 7 pain interference items from the BPI.[81-83] Because it is not well-established what level of improvement (or worsening) constitutes a clinically meaningful change in individuals with chronic pain, we will ask the study participants to both rate their pain and function as well as the importance of change at each assessment by asking them if they are "better," "about the same," or "worse," compared with the beginning of the trial.[49] This approach will enable us to classify each trial participant as "improved," "stable" or "worse" and conduct a secondary responder analysis.

Quality of life (QoL) will be assessed using the validated *Medical Outcomes Study Short Form Health Survey* (SF-12 v.2),[86,87] a common measure of health and QoL in CLBP.[60] The SF-12 has good reliability in CLBP (α=0.76-0.89), and yields one composite and two component (mental and physical) scores measuring general health and QoL.[86,87] Daily dose of prescription opioids for "the past 14 days" will be collected with the *Timeline Followback* (TLFB) method, a validated tool for daily substance use evaluation, with good test-retest reliability (0.79-0.97),[88-90] and verified against a participant's EHR data on prescribed opioids. This will enable longitudinal evaluation of opioid dose change during the proposed 52-week follow-up. We will work with the health systems to **leverage the EHR data and collect information on the prescribed opioids** at baseline and exit ("past 3 months"). The UW Health data analyst experienced in this methodology will ensure process integrity across the health systems and appropriate data linkage (**IR-2**). If needed, we will obtain prescription data through medical records with subject's written permission. **Because a health system's prescription record may not accurately reflect the actual use of opioids, we will rely on participant self-report as the**

primary measure of opioid use. To calculate a "daily opioid dose," doses of all reported opioids will be converted to a morphine-equivalent dose by multiplying daily dose of a given opioid by the published conversion factors, as we pilot-tested.[51]

D.7.b. Hypothesized Main Factors That May Impact Treatment Response (**RQ-4, HT-1**) Numerous factors can impact treatment response.[61] As outlined in *Background*, baseline negative affect and opioid misuse are the hypothesized main prognosticators of differential treatment response to MM and CBT in patients with opioid-treated CLBP.[34-38] Negative affect (anxiety, depression) will be assessed by the *Hospital Anxiety and Depression Scale*,[91,92] the increased score of which has been associated with worse treatment outcomes in opioid-treated patients.[1,4,6] Opioid misuse behaviors will be assessed with the validated *Current Opioid Misuse Measure*[93] and *Opioid Compliance Checklist*,[94,95] both **developed by Co-Investigator Jamison**. We will also evaluate the potential impact of other factors that might influence treatment effects. Pain catastrophizing, pain acceptance and mindfulness level can impact outcomes in chronic pain[96,97] and will be assessed with the *Pain Catastrophizing Scale,*[98] the *Chronic Pain Acceptance Questionnaire*,[99] and the *Mindful Attention Awareness Scale*,[100] respectively. *Neuropathic pain* and *sleep problems* have been linked to worse outcomes as well[101,102] and will be evaluated with the painDETECT[103] and the "minimum data set" sleep questions,[61] respectively.

D.7.c. Other measures

D.7.c.A) Survey-based measures We will assess the impact of the interventions on health care utilization (number of primary care, specialty, urgent care, emergency department visits; number of hospital days) and loss of productivity (work/leisure), using a survey developed for prior studies.[30,54] We will collect data on potential covariates: treatment expectation/satisfaction (baseline/follow-up) with the Global Impression of Change survey;[104] non-protocol treatments and adherence to the intervention protocol (session attendance; self-reported home MM/CBT practice) with surveys developed by our team with input from patient-advisors;[36,58] and any adverse / side effects, evaluated at each assessment and documented using a standardized UW Institute of Clinical and Translational Research's form. We will also collect data on potential adverse effects in relation to opioid dose reduction through use of the 10-item brief validated instrument, the Short Opiate Withdrawal Scale, which assesses the presence and severity of opioid withdrawal symptoms.[105,106] Demographics will be collected using the NIH "minimum data set" form.[61]

Table 7. Outcome measures, their collection timeline (months) and time-to-complete (minutes) during the 12-month study.

Outcome		Measure		0	3	6	9	12
**Data provided by ALL study participants**				In-person	Virtually			In-person
**Primary/secondary outcomes**								
Pain intensity	Aim 1	4-item BPI pain severity scale		X	X	X	X	X
Physical function		10-item ODI; 7-item BPI pain interference scale		X	X	X	X	X
Quality of life	Aim 2	12-item SF-12	10-15 min	X	X	X	X	X
Opioid dose		TLFB (past 14 days)		X	X	X	X	X
		EHR		X				X
**Hypothesized main prognosticators**		14-item HADS	10-15 min	X	X	X	X	X
Depression		14-item HADS		X	X	X	X	X
Anxiety	Aim 3	17-item COMM; 8-item OCC		X	X	X	X	X

Opioid misuse						
**Other potential prognosticators**						
Pain acceptance	24-item CPAQ	X		X		X
Pain catastrophizing	13-item PCS 10-15 min	X		X		X
Mindfulness level	15-item MAAS	X		X		X
Sleep problems	4-items from "minimum data set"	X		X		X
Neuropathic pain	8-item painDETECT	X		X		X
**Other survey-based outcomes**	5-10 min	X	X	X	X	X
Health care utilization	5-items, team-developed survey	X	X	X	X	X
Loss of productivity	2-items, team-developed survey					
**Potential covariates**	5-10 min					
Tx expectation/satisfaction	1-item GIC	X		X		X
Non-protocol Tx	2-items, team-developed survey			X		X
MM/CBT home practice	diary	X	X	X	X	X
Adverse effects	diary; ICTR form; SOWS	X	X	X	X	X
Demographics and updates	demographics from "minimum data set"	X	X	X	X	X
**Brief videorecording interview**	Qualitative analysis of comments					
Pain, function, well-being	study developed scoring scales	X				X
**Data provided by a SUBGROUP of participants (up to 60 subjects in total or 30 per study arm)**						
In-depth qualitative interview	interview by phone 30-45 min					X

COMM: Current Opioid Misuse Measure; CPAQ: Chronic Pain Assessment Questionnaire; GIC: Global Impression of Change; HADS: Hospital Anxiety and Depression Scale; ICTR: University of Wisconsin Institute of Clinical and Translational Research; OCC: Opioid Compliance Checklist; PCS: Pain Catastrophizing Scale; MAAS: Mindful Attention Awareness Scale; ODI: Oswestry Disability Index; SF-12: Medical Outcomes Study 12-item Short Form; TLFB: Timeline Follow Back; Tx: Treatment; SOWS: Short Opioid Withdrawal Scale

D.7.c.B) Qualitative measures The integration of quantitative and qualitative measures will offset the weaknesses inherent in one method with the strengths of the other method, and help expand our understanding of treatment effects. We will include two types of qualitative assessments designed to answer two different questions.

a) Brief (3-5 min) videorecorded interview of **all participants** at baseline and exit will complement quantitative measures of pain, function and QoL, and provide context to our quantitative measurement of outcomes.[107,108] Selected recordings will bring patient voices and experiences into view and help promote dissemination.[109] **The use of brief video-recorded interviews for outcome evaluation is novel and patient-driven;** it was recommended by **Penney Cowan, a patient advocate** (see E) and endorsed by our Patient Advisors. The premise is to enable a comparison of participant's qualitative presentation of pain, physical function and well-being (as a proxy for QoL) over time and across the two study arms, and to contrast it with the results from the validated PROs. We will use a simple semi-structured interview and task. We will ask subjects about their thoughts and feelings about a simple physical activity, then ask them to perform it, if they are able. One of the possible tasks is the Timed Up and Go (TUG) test,[110,111] which is standardized and routinely used in clinical settings, including UW Health. TUG takes 1-3 minutes to complete and includes getting up from the chair, walking 3 meters, then coming back to sit down. The task performance can be scored based on the observer's rating of the patient's risk of falling (1-5 scale)[110] or be timed.[111] We will work with Patient Advisors to finalize the protocols for the interview questions and the activity task. The interview will be carried out by a trained researcher. The recordings

will be uploaded to a secure online storage managed by the UW Department of Family Medicine and Community Health's IT Department. Each video will be owned by the recorded participant. The two recordings of each participant will be "spliced" together for easier comparison and mailed on a DVD to that participant at the end of the study upon request. Authorized researchers will have a remote access to the recordings for viewing, editing and analysis. The analysis will be supervised by **David Woods**, an expert in this methodology, with the assistance from **Linda Oakley** who is experienced in qualitative methods and in clinical care for this patient group (see *Letters of Support*). The evaluation of these videos will focus on both qualitative and quantitative aspects. Participant's qualitative comments will be analyzed using the qualitative analysis methods and the *Transana*® software,[112-114] developed by Dr. Woods for qualitative analysis of video recordings. We will develop simple rating scales that will quantify the extent to which a participant displays pain, ease of physical activity and general well-being. **This brief interview will not add a substantial burden, is feasible in the context of already existing infrastructure and team expertise, is of high interest to patients, and is patient-initiated.** With participant permission, we will use exemplary videos to promote result dissemination.

b) In-depth exit interviews (30-45 min) with a **subset of up to 30 participants from each arm** will allow for in-depth exploration of the experience of those who reduced their daily opioid dose the most. They will be conducted by phone after the participant – in a Boston, Salt Lake or Madison site - exited the study by the qualitative methods specialists from the UW Survey Center. The in-depth interviews will **focus on expanding our understanding of experiences that facilitated, and were related to, opioid dose reduction** (e.g., Was dose reduction patient-driven? Did other outcomes of interest improve as well? Were MM vs. CBT skills useful and applicable to this process?). The **UW Survey Center**'s team will help develop the protocols, conduct the interviews, and assist with data transcription, analysis and result summary, as outlined in their *Letter of Support*. They will work with our research and stakeholder team to optimize and then continue fine-tuning the protocols in an iterative manner based on the information from initial interviews.

D.8. Statistical Considerations

D.8.a. Analysis Plan **(IR-3)** The primary analysis will be based on the intent-to-treat principle, including all randomized participants regardless of adherence to assigned treatment. Descriptive statistics will be calculated on demographic variables and baseline outcome measures. The primary analysis will be tested in the framework of linear mixed effects to examine the comparative effectiveness of treatment on the change in pain severity, function (Aim 1) and QoL scores and opioid dose (Aim 2) over 12-months between the two groups. Tests will be performed at a two-tailed significance level of 0.025 for each co-primary outcome (Aim 1) and a 0.05 significance level for the secondary outcomes (Aim 2). Included in each model will be dummy variable for treatment, an effect for each of time and site, time by treatment interaction, and a random intercept for subject. Contrasts will be used to test for an overall treatment effect at 6 and 12 months. Subsequent to primary analyses, for any outcome found to be statistically significant, the effect at each follow-up will be tested. If the primary superiority analysis does not find significant differences between the two groups for either of the two co-primary outcomes, a secondary non-inferiority analysis will be conducted. That non-inferiority analysis would employ linear mixed effects models to determine 95% confidence intervals for the difference between the mean scores of the co-primary outcomes of pain and function, with the purpose of assessing whether mindfulness-based therapy is non-inferior to cognitive behavioral therapy.[127, 128] These analyses will use acceptable difference delta values of 0.8 for the BPI pain scale and 8.0 for the ODI function scale, which are (1) consistent with best literature including IMMPACT recommendations, (2) less than the proposed superiority margins of 1.0 for BPI

and 10.0 for ODI, and (3) suggested to us by external “arms length” experts while both they and we were completely blinded to trial results.[129] For Aim 3, to determine prognostic factors of treatment effect, a linear mixed model analysis will be performed for Aim 1 and Aim 2 outcomes, adjusting for potential confounders, including an indicator for site and a random intercept for subject. The method of maximum likelihood will be used to estimate the model parameters in the linear mixed models. Given the diverse nature of our sample, we plan to evaluate the effects of the intervention on Aim 1 and Aim 2 outcomes for a limited number of subgroups **(HT-1,2,3,4)** based on race/ethnicity (African American, Hispanic, non-Hispanic White), gender, baseline negative affect (score >11 vs. score ≤11 on the HADS survey),[39,91] and baseline risk of opioid misuse (score ≥9 vs. score <9 on the COMM survey).[115] The proposed subgroup analyses are based on the assumptions for the prevalence of elevated scores of negative affect (score > 11 on the HADS) and opioid misuse behaviors (score ≥ 9 on the COMM) that indicate a person’s increased risk for anxiety, depression or opioid use disorders.[39,40,115] In the studies by Co-Investigators Jamison and Edwards and their colleagues, 42%-48% of patients with opioid-treated chronic pain scored ≥ 9 on the COMM, and 35% had a total HADS score > 11.[39,93] Therefore, we anticipate that approximately 45% of the sample will have elevated levels of opioid misuse behavior (score ≥ 9 on the COMM) and approximately 35% of the study participants will have an elevated level of negative affect (score > 11 on the HADS). These subgroup analyses will evaluate the internal consistency of intervention effect (subgroup x intervention interaction tests). The exploratory analyses, using linear mixed models, will evaluate the impact of participant adherence to the intervention protocol (session attendance; minutes/week of home practice) on treatment effects on Aim 1 and Aim 2 outcomes; covariates will include those of the primary analysis. All effectiveness analyses will explore the potential impact of the intervention delivery mode (in person versus virtual). Missing data (**MD-1,2,3,4,5**): Although we will monitor and enhance retention and protocol adherence (see D.6.d), and monitor data quality during collection, entry and maintenance, we anticipate some missing data. The maximum likelihood method will account for missing data on main outcomes; this approach is expected to be more robust to missing data under missingness at random (MAR) assumption than non-likelihood approaches, such as generalized estimating equations.[116] Missing data on covariates will be imputed using multiple imputation (i.e., chained equations) technique, which produces valid statistical estimates under MAR and accounts for the uncertainty arising from missing data.[117,118] If a participant drops out, we will document the reason and whether the dropout involves some or all types of participation. We will attempt to contact study subjects to collect data on at least the primary and secondary outcomes. All subjects will be accounted for in the reports. Sensitivity analysis (**MD-5; IR-5**) Using multiple imputation techniques, we will assess the degree to which results are sensitive to the MAR assumption. [119] To assess the normality assumption of the linear mixed models normal q-q plots of the residuals will be used. To examine the sensitivity to model assumptions, robust nonparametric method[120] will be used to evaluate treatment effects without distributional assumptions; if the results differ from those of the maximum likelihood approach both sets will be reported, else only the maximum likelihood results will be reported. Additional analyses: We will characterize each study group at each time point by reporting change in primary and secondary outcomes from baseline. According to the IMMPACT recommendations, we will also report on the entire distribution of treatment response by reporting the proportion of responders (or those who worsened their status) for all percentages of score change from 0% through 100%.[49] Specifically, we will present results by percentage of participants who a) improved their scores (including those who improved by 30% or more); b) worsened their scores (by percentage of worsening); and did

not change their scores. In addition, we will provide a detailed description of the change in daily opioid dose over time, by group status, and assess the magnitude and statistical significance of opioid dose change within and between groups over time. The opioid dose change reporting will include a proportion of patients with: a) any dose reduction; b) 25% dose reduction; and c) 25% dose increase ("dose escalation") from baseline to 12 months. We will establish thresholds for what constitutes a clinically important change in the main outcomes for the study participants; this approach will enable us to classify each trial participant as "improved," "stable," or "worse" and conduct a secondary responder analysis.[49]

Qualitative analysis methods will be applied to qualitative data. The **brief videos** will be analyzed both *qualitatively* (themes that were brought up by participants) with the *Transana* software developed by **David Woods**, and *quantitatively* to rate the perceived pain, function and general well-being. Data from the qualitative and quantitative assessment of these brief videos will be used for mixed-methods analysis of within and between group differences to better understand the impact of each study intervention on pain, function and QoL. For **in-depth qualitative interviews**, the UW Survey Center team (2-3 analysts) will inductively develop a thematic coding scheme based on the initial 4-5 interviews. This scheme will be shared with the investigative and stakeholder team to validate the themes; this is expected to be an iterative process, with ongoing feedback from all collaborators. The finalized coding scheme will be applied, using NVivo software,[121] to all transcripts. A summary report will be prepared by the UW Survey Center, with the input from research and stakeholder team members.

D.8.b. Sample Size Justification Using estimates from systematic reviews and meta-analyses, comparing MM and CBT to a wait-list controls,[11,20-22,32] and from two previous trials of MM in opioid-treated chronic pain,[30,31] as outlined in Gap Analysis (*Background*), we assume an effect size d=0.25 of MM vs CBT for pain severity and function, and a dropout rate of not more than 20% (seeC.2.b). We adjust for multiplicity of the co-primary outcomes by using a significance level of 0.025 for each Aim 1 outcome, for an overall significance level of at most 0.05. With 383 subjects randomized to each of the treatment arms (766 total), a two-sample test with a significance level 0.025 and at most a 20% dropout rate is powered to detect an effect size of 0.25 of MM versus CBT for primary outcomes with power of 0.80.

To assess power to detect treatment differences between the proposed subgroups, we used the sample size of N=383 per group and a significance level α=0.05 (i.e., not adjusting for multiplicity). Based on the existing literature and prior studies by Co-Investigators, we anticipate that approximately 35% of the study participants will show an elevated level of negative affect and approximately 45% of the sample will have an elevated level of opioid-related aberrant drug use behaviors (see D.8.a). For the power to assess Aim 3, we wish to be able to detect a difference of 0.5 (moderate effect size) between the subgroups, assuming no effect for the subgroup with less pathology (lower HADS or COMM scores) and an effect size of 0.5 for the intervention effects in the subgroup with elevated negative affect and/or opioid misuse scores. We chose the effect size of 0.5 for the proposed subgroup analyses per existing recommendations; this doubles the proposed main effect size of the study, assumed to be 0.25, for the subgroup hypothesized to benefit most from the intervention. [122] Based on the statistical simulations of size 10,000 (so that the margin of error is approximately 0.01) we conducted, we will have at least 80% power (84% for negative affect subgroup and 86% opioid misuse subgroup) to detect a significant (subgroup x treatment) interaction effect for each of these two main subgroups.

E. Engagement Plan (Criteria 2,4,5; PC-1,3,4; RQ-1,3,6)

The study team will include researchers and key stakeholders involved in life and/or care of patients with opioid-treated CLBP. A group of 10-12 patients and family members will form a Patient/Family Advisors (PFA) group (*Table 8*). The PFA

will meet in person up to 3 times/year; 2 patients and 2 family members will represent the PFA at the Study Advisory Committee (SAC) meetings. The SAC will include at least 10 stakeholder partners (*Table 8*). The SAC team members and the investigative team members (PI; 2-4 Madison, 1-3 Boston, and 1-3 Salt Lake researchers) will meet twice/year on average and up to 3 more times/year additionally throughout the project. The investigators will work closely with leaders of the health systems, meeting with them at least annually, to bring the leaders' input to the study team. The stakeholders have advised on this project since its inception (see D.2) and will continue to be **reciprocally** engaged as study team members (*Table 9*). To facilitate an effective and sustainable **partnership**, we will work with WINRS to develop a tailored training program for PFA members in Year 1; WINRS will continue to consult on effective meeting practices and other strategies to support meaningful patient stakeholder engagement across the project lifespan (*Table 8*), as outlined in the *Letter of Support* from **Betty Keiser and Gay Thomas**, co-directors of the WINRS. The WINRS' program of Community Advisors on Research Design and Strategies (CARDS®) will advise on the study materials and recruitment strategies (Table 9); the CARDS® and its services are detailed in *Appendix C*. The WINRS will also co-facilitate the PFA and SAC meetings jointly with the researcher team members to help **promote co-learning** in the spirit of **transparency, honesty, trust and equal-partnership** – values we will deliberately promote and are committed to.

Table 8. The study team will include both researchers and stakeholders.

Group	Members of The Stakeholder Group
**PFA**	**10-12 members:** 5-6 patients with opioid-treated CLBP and 5-6 family members/caregivers<br>Two patient-advisors have been identified*****
**SAC**	**Patient members (2):** 2 patients with opioid-treated CLBP representing the PFA<br>**Family members (2):** 2 family members/caregivers representing the PFA<br>**Physician members (2):** family physician **Russell Lemmon*** and general internist **Elizabeth Jacobs**<br>**Physical Therapist (1):** physical therapist **Evan Nelson**<br>**Psychologist member (1):** health psychologist **Chantelle Thomas*** (Access clinic for underserved populations)<br>**Patient advocate member (2): Penney Cowan*** (American Chronic Pain Association of chronic pain patients); **Christin Veasley** (Chronic Pain Research Alliance)<br>**Community advocate member (1): Cheryl Wittke*** (Safe Communities; local non-profit coalition)<br>**Primary Care Clinicians professional organization representatives (1-4):** Elizabeth Jacobs (Society of General Internal Medicine)
**WINRS**	**WINRS** will be engaged throughout the project to help train the patient stakeholders and enhance their engagement, and support researcher-stakeholder partnership; WINRS's group of community advisors (**CARDS**) will also advise (*Table 9*)

* provided *Letters of Support;* CARDS: Community Advisors on Research Design and Strategies®;
WINRS: Wisconsin Network for Research Support; PFA: Patient/Family Advisors; SAC: Study Advisory Committee

Table 9. Stakeholders Are Involved at Every Stage of The Proposed Project: 1) Planning, 2) Conduct and 3) Dissemination.

Stage of the Project	Investigator Responsibility	Stakeholder (PFA, SAC) input
**1) Planning the Study**		
Research topic and question selection	Clinical experience, review of evidence to identify gaps: MM needs to be assessed in opioid-treated CLBP	Clinicians, patients with opioid-treated CLBP and family members confirmed the relevance and importance of MM to be tested; health system leaders were in agreement (see D.2)
Study design	Two study design choices: MM vs CBT or MM vs CBT vs wait-list control	Patients advised against a wait-list control group: lack of active treatment would not be “fair” to the control group subjects
Outcomes selection; ways of data collection	Review of existing guidelines on the selection of outcomes; optimal ways for data collect ion	Patient, family members and clinicians endorsed the choice of outcomes and prioritized them; patients recommended to maximize virtual data collection (see D.6.c; D.7.a)
**2) Conducting the Study**		
Study materials and protocol	Research team will draft the study materials and protocol, including the recruitment strategy	PFA and CARDS will advise on these drafts to ensure that we limit subject burden, encourage recruitment, retention and adherence; on the outcome measure packet before it is finalized; on the “official” study’s name; in addition, health systems leaders will advise on recruitment protocols
Data and Safety Monitoring Committee	Research team will invite the Committee members to participate	We will invite 1-2 representatives of the PFA group to join the Committee to ensure patient-centeredness
Recruitment, retention, adherence, engagement of participants	Research team will monitor and encourage recruitment, retention, adherence and engagement (see C.2.c; D.6.d)	The plan for enhancing recruitment, retention, adherence, and engagement, and challenges in these areas will be discussed with PFA, CARDS, and SAC; the WINRS team will monitor and advise on stakeholder engagement throughout the study
Protocol for exit in-depth qualitative interviews	Research team and UW Survey Center will draft this protocol	PFA and SAC will review and advise to ensure the qualitative interview questions are appropriate
Quantitative data: collection, analysis, interpretation	Research team will be responsible for high-quality data collection, clean-up, entry, analysis and write-up of results	PFA and SAC will review data collection processes if there are problems with missing data; we will discuss the main analysis plan and then the results prior to finalizing the interpretation
Qualitative data: analysis, interpretation	Research team will be responsible for high-quality data collection, transcription, analysis and write-up	PFA and SAC will advise on the identification and interpretation of major themes before the coding of qualitative data is finalized; input on result interpretation
**3) Disseminating the Study Results**		
Dissemination	Research team will draft presentations, manuscripts, and other dissemination materials (e.g., will select video recordings to post online) and will make the intervention manuals available	PFA and SAC will advise on result interpretation; will review the result summaries to ensure the results are communicated in meaningful, usable ways; will be encouraged to co-present, co-author the dissemination materials; community and patient advocates, Cheryl Wittke and Penney Cowan, and the representatives of the primary care provider professional organization (members of the SAC) will help dissemination to end-users via their websites

CARDS: Community Advisors on Research Design and Strategies®; WINRS: Wisconsin Network for Research Support;
PFA: Patient/Family Advisors; SAC: Study Advisory Committee

F. Research Team and Environment

The study team has expertise in mixed-method clinical trials of MM and CBT interventions, recruitment and retention of patients with chronic conditions, including opioid-treated chronic pain, engagement of those from hard-to-reach

populations, and dissemination of results. A major strength of this team is that it includes both experienced researchers who have a track record of working well together and engaged advisors representing diverse stakeholder groups committed to improving health of people with opioid-treated CLBP (see *Biosketches and Letters of Support*). The broader environment and resources offered by the University of Wisconsin (UW), Harvard University and the University of Utah, including funds for the costs of intervention delivery (see *Institutional Letters of Support*), will enable high-quality patient-centered study. With high patient and clinician interest in MM as a means to reduce the impact of CLBP and opioids, and strong support from health systems, which will serve as a recruitment base, we are well-positioned to successfully carry out this study.

Aleksandra Zgierska MD PhD (Dual-PI) will lead the study and the Hershey site, and be responsible for the overall study conduct and completion: engaging and working with stakeholder partners; finalizing the study protocols; development and management of the databases; ensuring quality of data collection, analysis and write-up; safety monitoring; and reporting back to PCORI. She is a practicing family medicine and addiction medicine physician, a tenured Professor at the Pennsylvania State University, and has expertise in opioid therapy and clinical trials of MM for chronic conditions. She led two RCTs of MM interventions –similar to that proposed– for alcohol dependence and opioid-treated CLBP during her recently-completed NIH-funded K23 Career Development Award (K23AA017508). This latter study[30] served as a pilot for the proposed RCT. She has collaborated with numerous Investigators, many of them forming the current study team, on studies evaluating the impact of promising interventions for improving health outcomes. In addition to leading research projects, her expertise and leadership have been recognized. She was invited by the NIH and the Department of Defense to serve as a reviewer for scientific proposals on MM as a therapy for addictive disorders; elected Vice-President (2011) and President (2014) of the Wisconsin Society of Addiction Medicine; and invited to teach and present nationally and internationally on topics related to MM as a therapy for refractory clinical conditions. **Bruce Barrett MD PhD** is a practicing family physician and tenured Professor in the UW Department of Family Medicine and Community Health (DFMCH). He has led three NIH R01-funded mixed methods RCTs, two of which compared MM to both an active intervention as well as a non-interventional control condition. In addition, Dr. Barrett has led other patient-centered outcome studies evaluating effects of Complementary and Integrative Health (CIH) therapies. He directs the HRSA T32-funded Primary Care Research Fellowship, and Vice Chair for Research, leading the UW DFMCH research division. As a physician-investigator and Dual PI, he will provide guidance on study conduct, data analysis, result interpretation and write-up, and will provide medical support to study staff regarding participant health concerns and safety. The Dual PIs experience will be augmented by the extensive expertise of other team members. The Dual PIs experience will be augmented by the extensive expertise of other team members. **UW Faculty: Nalini Sehgal MD** is a pain medicine physician with clinical and research expertise in opioid-treated chronic pain. **Linda Oakley PhD PMH-NP** is a psychiatric nurse practitioner with expertise in engaging hard-to-reach populations and qualitative methods. **Marlon Mundt PhD** is a statistician with expertise in RCT and PCOR analytical methods. Consultant **Richard Davidson PhD** is a world-expert in MM and related research. Collaborators **Shilagh Mirgain** and **Janice Singles** are pain psychologists experienced in MM and CBT; they will deliver the study interventions in Madison. Collaborator **David Woods PhD**, expert in qualitative analysis of video materials, will assist with the analysis of the brief video interviews. The **UW Survey Center** specializes in qualitative methods and will conduct, analyze and summarize the content of in-depth interviews. The **Wisconsin Network for Research Support and its CARDS® group** will help ensure patient-centeredness and strong, productive patient, family and community stakeholder engagement. In addition, consultant **Elizabeth Jacobs MD MAPP**, an internist-physician and a faculty member at the Maine Medical Center

Research Institute is a nationally-recognized researcher with expertise in health disparities research and conduct of comparative effectiveness PCORI-funded multi-centered trials, and will advise on the stakeholder engagement, and study recruitment, retention and overall conduct. **Harvard Faculty: Robert Edwards PhD** and **Robert Jamison PhD** are clinical psychologists and experts in CBT application to chronic pain and in the assessment of pain, function, and opioid related outcomes. **Edward Michna MD** and **Edgar Ross MD** are pain medicine physicians with experience in pain treatment and outcomes research. The Boston team, led by Dr. Edwards, have collaborated on numerous NIH-funded clinical trials of behavioral interventions for opioid-treated chronic pain.[41,57] They will be responsible for finalizing the protocols related to CBT intervention and its delivery, the local recruitment and study conduct, and will assist in all study-related activities. **University of Utah Faculty: Eric Garland PhD** is a clinical social worker and clinical trial researcher with expertise in both MM and CBT therapies for chronic pain and opioid misuse. His NIH-funded RCT of the MORE (Mindfulness-Oriented Recovery Enhancement) intervention, developed by him, showed a reduction of pain, pain-related interference, opioid craving, and opioid misuse behaviors in opioid-treated chronic pain patients.[31,123,124] The main focus of his research is on developing and testing interventions to reduce opioid misuse and abuse among chronic pain patients (https://drericgarland.com/); this focus will perfectly complement, while not overlapping with, the goal of this study to reduce a reliance on opioids among adults with opioid-treated CLBP. **Yoshio Nakamura PhD** is a psychologist, expert in clinical applications of mind-body therapies for chronic conditions, with a methodological focus on the measurement and analysis of individual factors that predict treatment outcomes– germane to Aim 3 of this study. **Julie Fritz PT PhD** is a physical therapist and nationally-recognized expert in research on low back pain treatment who will contribute her skills in utilizing the electronic health record for participant recruitment and data extraction. They, along with the PI, will be responsible for finalizing protocols related to participant recruitment and opioid prescription data extraction protocols, the local recruitment and study conduct, and will assist in all study-related activities. The study team will also include at least 10 **SAC members** representing the vital stakeholder groups and integral partners throughout this project (see E).

Research staff have been an integral part of the team's success. The Madison staff have worked with the PI and Co-Investigators on similar studies and are familiar with all aspects of the proposed project. **Cindy Burzinski, MS** served as a coordinator for the PI's pilot RCT of MM for opioid-treated CLBP, has been involved in the development of this proposal and will serve as the Study Manager for the proposed study. The team will communicate and meet regularly. We will regularly meet for the Investigator and Stakeholder team meetings, and more frequently as a site study team at each site.

DISSEMINATION AND IMPLEMENTATION POTENTIAL

A. Describe the potential for disseminating and implementing the results in other settings (PC-4)

Our team is well-poised to broadly and effectively disseminate the results of the proposed research and facilitate implementation of MM intervention to promote improved health of adults with opioid-treated CLBP across the US. Results of this research could be readily disseminated. The PI's pilot study of adults with opioid-treated CLBP provides support for MM as effective, feasible and acceptable in this patient population (see B.2; B.3),[30,42] all essential aspects to successful implementation. Conclusive positive evidence in the proposed study on the effectiveness of MM, high public and clinical interest in this modality, and strong assembled coalition of partners, will help add MM to standard-of-care therapy, making it accessible to patients with opioid-treated CLBP.

Our team has experience with dissemination through scientific presentations, publications and patient-oriented methods. **Elizabeth Jacobs** has created an alliance of stakeholders to promote dissemination and implementation in her ongoing PCORI-funded study (CER-1310-07844). We will use this approach to ensure successful dissemination and translation of MM into clinical practice. We have engaged key-stakeholders to ensure the study findings are useful for patients. We are working with large health systems, the engagement of which will promote dissemination and implementation (see *Letters of Support*). In Madison, all five major local health systems have enthusiastically agreed to collaborate and expressed interest in implementing the MM intervention if effective. We will deliberately recruit patient-advisors from each health system to ensure that patient-advisor feedback is most relevant to patients and collaborating systems. In Boston, the Healthcare Partners System serves 1.65 million patients, providing a robust base for dissemination and implementation. In Salt Lake City, the 11 University of Utah Health Care primary care clinics serve 120,000 patients, and the broader health care system is comprised of multiple hospitals and over 80 affiliated clinics. We will draw on the experience of clinicians and their patient-subjects. The network of clinicians in the involved health systems can be a powerful source for supporting implementation of effective approaches. Importantly, the MM intervention can be implemented through an existing clinical infrastructure, in a similar way as other psychotherapies are currently delivered. Forms of MM programs have already been practically disseminated in clinical settings. For example, MM-based programs, including the one proposed here, are often patterned after MBSR,[16] which has been widely implemented in the US and abroad.[125] However, currently the MM modalities are not typically covered by health plans in spite of interest of patients and clinicians in these approaches (see B.1).[10,30] Results indicating MM's effectiveness could remove this barrier and encourage health insurance coverage, increasing accessibility to patients. Strong public interest in MM is endorsed by the key stakeholders (see *Letters of Support*) and by the willingness of the Madison, Boston, and Salt Lake City clinical departments to cover the intervention delivery costs during the study. Availability of an existing, detailed, step-by-step manual for intervention delivery will also facilitate implementation. This has been our experience with our prior study, which evaluated a similar MM intervention for alcohol relapse prevention. In that RCT (N=123),[54] the PI collaborated with all major local addiction treatment centers, developed the intervention manual, and made it available to the involved centers. Alcohol-dependent patients showed strong interest in MM, the subjects were satisfied with the intervention and viewed it as useful for relapse prevention. In the context of ineffective

therapies for addiction and the positive experience of the participants and the treatment centers, there was a rapid uptake of this MM modality. Currently, the MM-based approach is standard-of-care at the majority of the treatment centers in Madison, in spite of the limited pilot-level. A hands-on training for clinicians interested in delivering the MM intervention could be offered, based on the successful model of Mindfulness-Based Relapse Prevention workshop,[68] which provided training for the PI; this model will be utilized for training of the study therapists in intervention delivery, as we pilot-tested.[30,54]

The local and national patient and community advocacy, and primary care provider professional organizations will also aid dissemination. Safe Communities (www.safercommunity.net) is a non-profit coalition of over 350 private and public entities, including the leadership of all local health care systems, patients, first responders, law enforcement, area businesses, schools and youth serving agencies, senior centers and faith communities. Its mission is to improve community health. Their recent efforts have centered on reducing the impact of prescription opioid epidemic. **Cheryl Wittke**, Director of Safe Communities, has expertise in mobilization and hosting members of the community and its leaders around issues critical to the health of the communities in which they exist. The recent Health Care Task Force on Safe Opioid Prescribing brought together patients, clinicians, health systems and policy makers to brain-storm and recommend solutions for improving opioid prescribing practices. Our involvement with Ms. Wittke as a stakeholder-advisor will help us align our goals and methods with the needs of the community; in return, Safe Communities will help facilitate future dissemination of findings through their robust coalition of partners and website (see *Letter of Support*). The American Chronic Pain Association (www.theacpa.org) is an organization for persons with chronic pain. Its mission is to facilitate peer support and education for patients and their families so that they may live more fully in spite of their pain. The effects of opioids and ways to reduce patient's reliance on opioids are important topics for the Association. The Chronic Pain Research Alliance (www.chronicpainresearch.org) is the country's first and only research-led collaborative advocacy effort dedicated to changing the lives of those with chronic pain. Its mission is to advance timely diagnoses and effective evidence-based medical management for individuals affected by chronic pain. **Penney Cowan**, the Association's founder and director, and **Christin Veasley**, the Chronic Pain Research Alliance co-founder and director, will be involved in the study as patient advocate-stakeholders. They will attend the SAC meetings and are enthusiastic about the study, as they view MM as an important, underutilized treatment modality for opioid-treated chronic pain (see *Letter of Support* from Ms. Cowan). Upon Ms. Cowan's recommendation, we included a brief videorecording as one of the outcome measures. In addition to their utility for assessing change, selected video-recorded vignettes will also serve as a tool for result dissemination; they could be posted on the Association, Alliance and Safe Communities' websites to better convey the message about the effects of MM and the experience of study participants. We will also involve a representative or representatives from the professional organizations of primary care clinicians. Consultant Jacobs, as the president of the Association of Chiefs and Leaders in General Internal Medicine and a member of the Executive Committee of the Society of General Internal Medicine (SGIM), will serve as a liaison between the SGIM and the study team (see Letter of Support from SGIM). We will consider adding representatives of their organizations as appropriate.

B. Describe possible barriers to disseminating and implementing the results of this research in other settings.

In our team's experience, the greatest barrier to dissemination of information and implementation is communicating effectively with the target audiences and effectively translating findings into actual practice on the ground.

We expect to be successful at minimizing these barriers because:

(1) Our team is experienced in framing messages in ways that will be widely accepted by relevant audiences.

(2) We have experience in implementation of evidence-based interventions.

(3) MM-based therapies are successfully in practice across the US, suggesting that the proposed MM intervention could be practically implemented should evidence suggest effectiveness for patients with opioid-treated CLBP.

4) We will engage all key stakeholders in an ongoing effort throughout the study period so that dissemination and implementation will be an obvious next step to our target audience. In the proposed project, stakeholders will assist in developing strategies for subject recruitment and retention, materials to describe the study, finalize the methods, and then to disseminate findings to local and national agencies (see E).

REPLICATION AND REPRODUCIBILITY OF RESEARCH AND DATA SHARING

A. Describe the ability to reproduce potentially important findings from this research in other data sets and populations.

In order to support and promote efforts by other researchers to replicate study findings in other patient populations and datasets, we have developed the following reporting and replication plan (**IR-2; IR-6**):

Registration: We will register our trial on www.clinicaltrials.gov in order to ensure transparency of our research and comply with ethical standards of the field.

Study protocol and materials: We will provide PCORI a final protocol of the study when all study activities are complete that includes all changes made throughout the process. A version of the relevant protocol will also be included in our 6-month progress report (see Milestones). All versions of the protocol will be saved with track changes and will include reasons for changes throughout the study. We have developed a detailed study protocol for the PI's pilot study[30] and will expand and adapt it for the proposed project.

The protocol will include:
- Project background
- Study aims
- Selection of participants (including inclusion and exclusion criteria)
- Research design and methods (including screening, recruitment, description of study instruments and their intended measurable outcomes and administration). This will include a flow diagram of study procedures as well as the procedures for the intervention implementation.
- Schedule of events
- Consent/HIPAA processes
- Compensation scheme
- Statistical considerations
- Analysis plan (qualitative and quantitative, including outcomes, covariates)
- Risks and protection against risks for research subjects
- Potential benefits
- Records to be kept
- Data and safety monitoring plan

All data dictionaries, programming codes, qualitative codebooks, and other study materials developed will be provided to PCORI within 3 months of funding completion in order to promote replication of our research process. PCORI may share any and all of our materials with requested researchers after consultation with the PI, Dr. Zgierska.

CONSORT guidelines: We will track data and include descriptions of the study elements as suggested by CONSORT guidelines for the reporting of clinical trials.[65]

Data organization and management: Data will be organized following the **PCORnet Common Data Model** to standardize data labeling, entry and enable sharing.[126] To further promote data and information sharing, we will collect the "minimum data set" data, in addition to other outcome measures, as recommended by the NIH Task Force describing the best research practices for clinical trials on CLBP.[61]

We will apply a **rigorous, protocol-driven approach to data collection and management** to ensure high-quality data and reproducibility of the approach. Study-collected data will be stored "centrally" on a secure UW Department of Family Medicine and Community Health's server. These data will be accessible locally or remotely to authorized research team members in Madison, Boston, Salt Lake City, and Hershey who will be able to view and/or enter data. Data provided online by the study participants will be "deposited" directly into the UW-housed RedCap database, which will be developed and managed by **Larissa Zakletskaja**, a UW database specialist experienced with the proposed methods.[30,54-56] Data from the in-person assessment sessions (at baseline and exit) and data collected by a trained researcher virtually or using other methods will be entered by a research staff into the outcome database. Outcome data, labeled by the subject's study ID only, will be stored in an outcome database, separate from the identifiable data, used for subject tracking. The UW database analyst will ensure standardized extraction of EHR data across the health systems and will facilitate linking of de-identified EHR data to other outcome data. The videorecorded interviews and in-depth qualitative interviews will be stored on a secure UW server and managed by the UW Information Technology team.

B. State that a complete, cleaned, de-identified copy of the final data set used in conducting the final analyses will be made available within one year after the completion of the study. Propose a method by which investigators will make this data set available, if requested.

A final clean dataset will be available for data sharing within 12 months of the end of the project. All de-identified quantitative data will be exported from our database and available as csv file. Qualitative data will be made available including the coding taxonomy and coded themes. The coding taxonomy will include definitions and rich description in order to promote transparency of our coding structure. We will set up an online sharing tool for interested researchers in order to accommodate data sharing requests.

C. Propose a budget to cover costs of your data-sharing plan, if requested. These costs do not need to be included within the Detailed Budget.

No additional funds are requested at this point.

PROTECTION OF HUMAN SUBJECTS

The proposed Human Subjects Research meets the definition of a clinical trial. The study protocol will be in compliance with HIPAA and other federally mandated human subjects regulations and approved by the University of Wisconsin–Madison (UW) Human Subjects Committee of the Health Sciences Institutional Review Board (IRB), by the Brigham & Women's IRB within the Partners Human Research Committee (PHRC), by the University of Utah IRB, and by the Penn State College of Medicine IRB. All members of the research team will complete the protection of human subjects training required by the IRB prior to subject contact. The UW IRB approved the PI and other UW investigators' prior studies; the Brigham & Women's IRB approved prior trials of our Boston Investigators; and the University of Utah IRB approved prior trials of our Utah Investigators. Therefore, we do not anticipate problems with securing the IRB approval in a timely fashion. We will apply for the Certificate of Confidentiality if it is recommended by the IRB in either institution.

1. Human Subjects Involvement, Characteristics, and Design

Study design We propose a partially-blinded, two-arm multi-site mixed-method pragmatic RCT evaluating the comparative effectiveness of MM and CBT interventions on pain severity and function (Aim 1), and quality of life and opioid dose (Aim 2) in patients with opioid-treated CLBP over the 12-month follow-up period. We hypothesize that MM will have a more beneficial effect on these outcomes than CBT. We will also assess if certain baseline factors might influence treatment response, hypothesizing that those with worse negative affect and opioid misuse will respond better to MM than CBT in terms of the main outcomes. To test these hypotheses, 766 adults with opioid-treated CLBP will be randomly and equally assigned to either MM (N=383) or CBT (N=383) arms across the study sites led by Madison, WI (UW; PI: Barrett), Boston, MA (Harvard Medical School; PI: Edwards), and Salt Lake City, UT (University of Utah; PI: Garland). The proposed sample size was calculated based on existing data (see D.8). We will collect reports on pain severity, function, quality of life (QoL) and daily opioid dose with validated patient-reported outcome (PRO) measures at baseline, at 3, 6, 9, and 12 months post-entry. We will complement these survey data with qualitative data on pain, function and QoL obtained through brief video-recorded interviews and on opioid dose through EHR data at baseline at exit. We will apply in-depth qualitative interviews to a subgroup of participants (up to 30/arm) who completed the study to better understand factors related to opioid dose reduction. The proposed methods were successful in our prior studies (see D.6).[30,39-41,54-57,94,95] The proposed outcomes were endorsed by our patient-stakeholders.

Individual participant timeline Opioid-treated CLBP patients will be recruited from the collaborating health systems and from the community (seeC.2). Those interested and meeting the eligibility criteria will meet with the study coordinator . During this meeting, individuals consented and enrolled into the trial will be assigned a study ID number, complete baseline assessments, then be randomized, and scheduled for either MM or CBT intervention. The randomization sealed envelopes, prepared by the study statistician, will be distributed consecutively to participants by the study coordinator; the seal will be broken upon completion of baseline assessments. The study interventions will be manual-driven and consist of 8 weekly, two-hour group sessions, led by trained therapists (virtually or in person when available), and home practice for at least 180 min/week; participants will be asked to continue their home practice and track practice minutes throughout the entire study (see D.5). Each participant will be followed for 12 months and undergo 5 assessments. Two assessments (baseline and exit) will be conducted virtually or in person (when available); assessments at 3, 6, and 9 months will be completed using web-based reporting or by phone/mail or other virtual means per participant

preference (see D.6.b and D.6.c).[55,56] We anticipate the majority of subjects will use the web-based reporting.[55,56] Data on Aim 1 and Aim 2 outcomes, home MM/CBT practice and adverse effects will be collected at each assessment. Data on other outcomes will be collected at baseline, 6 and 12 months. We will complete in-depth qualitative interviews with a subgroup of up to 60 (30/arm) participants who completed the study and reduced their opioid dose; these interviews will be conducted by phone by a UW Survey Center qualitative specialist. These methods were feasible in our prior studies.[30,39-41,54-57,94,95] We will implement strategies to promote retention and adherence, and reimburse each participant for time/effort (see D.6.d). Each subject will be assigned a study representative to serve as the primary contact. We will have study-designated phone numbers for ease of contact. Subjects will receive reminder messages prior to scheduled meetings. Study personnel will inquire about subject experience, and solicit comments, questions, and potential problems during each contact. We will provide light snacks during the 2-hour intervention sessions and offer transportation if needed to study activities to further improve adherence and retention; we estimate that at least ~3% of study visits will require transportation assistance, usually a cab ride.[30,41,68]

Study population We estimate that approximately 19,000 patients may be eligible for study participation across the collaborating sites (see C.2), providing a robust recruitment base. Based on our prior trials, we estimate 3,830 individuals will need to be screened to result in 766 enrolled people (see C.2). **Eligibility screening** will be based on self-report, conducted virtually, and take 10-15 minutes to complete. **Inclusion criteria**: English-fluent adults ≥21 years; CLBP as the main source of chronic pain of at-least moderate severity (score ≥3 points) and at-least moderate disability (Oswestry Disability Index score ≥21 points), treated with ≥15 mg/day of morphine-equivalent dose for ≥3 months. **Exclusion criteria**: prior formal MM or CBT training; inability to safely or reliably participate; current pregnancy, pre-existing borderline personality, delusional or bipolar (manic) disorders. Competent adults, meeting eligibility criteria, will be able to participate regardless of gender, race, ethnicity, religion or socioeconomic status. Prisoners, pregnant women and mentally impaired persons will not be included. Children, per eligibility criteria, are not eligible.

2. Sources of Materials The data obtained from human subjects for this research will consist of survey and qualitative data, and EHR-based data on prescribed opioids (see D.7). All data will be obtained specifically for this project. None of the study's procedures or measures is designed for clinical diagnosis or treatment. **Survey data** will include information on demographics, pain severity, function, quality of life, opioid medication dose, opioid misuse behaviors, psychological health, pain coping, mindfulness, neuropathic pain, protocol adherence, health care utilization, productivity, subject treatment preferences and experiences, non-protocol treatments, and adverse events. **Qualitative data** will be collected through brief (3-5 minute long) videorecorded interviews with all participants (baseline and exit) and through in-depth interviews with a subgroup of participants (up to 30/arm). The goal of brief videorecordings is to provide qualitative information on pain, function and well-being and to better capture participant experience. The videos will owned by each filmed participant who, at the end of the study, can receive his/her videos on a DVD. With participant permission, we will use selected videos for dissemination of results (e.g., via websites of our stakeholder-partners). The in-depth interviews, conducted by phone with a subgroup of participants, aim to deepen our understanding of factors and experiences related to opioid dose reduction. **EHR based data** on prescribed opioids ("past 3 months") will augment and corroborate participant's self-report on opioid use. UW Health data analyst, jointly with the PI, will develop protocols for data extraction, using published criteria and feedback from the collaborating health systems; all the systems use the same EHR software. Two health systems in Madison (UW Health and Access), the health system in Boston, and the health system in Salt Lake City have worked with the members of our team in the past on extracting such data. SSM

Health, Group Health Cooperative and UnityPoint Health-Meriter will start working with us on this process early in the project; we budgeted funds for data analysts in these three systems so that it is feasible for them to initiate data extraction protocols. In case this is not feasible, we may, with subject written permission, request data on prescribed opioids through medical record archives.

Data management and protection At enrollment, subjects will be assigned a unique ID number, which will be used to label all data collection instruments, qualitative recordings, and EHR data, and to link results to a particular subject in the study database. Identifying information, other than subject ID code, will not be present on any documents or in outcome databases. A master list with subject identifying information will be securely stored electronically and on a paper version which will be stored in a locked filing cabinet in the locked PI's or research coordinator's office, separately from outcome data. Only the PI and designated study personnel will have access to identifying information. **REDCap electronic database**, developed and managed by the UW database specialist **Larissa Zakletskaia** will provide data management, functionality, and support for data capture. REDCap uses MySQL databases via a secure web interface with data checks during data entry to ensure data quality, and includes a complete suite of features to support HIPAA compliance, including a full audit trail, user-based privileges, and integration with the institutional LDAP server. The MySQL databases and the web server are both housed on secure servers operated by the UW Department of Family Medicine and Community Health (DFMCH). Access to study data in REDCap is restricted to IRB approved members of the study research team by username and password.

3. Potential Risks The proposed interventions are not anticipated to lead to serious side effects or adverse events, as based on the existing literature[11,21,22] and our prior studies.[30,54-56] Behavioral treatments may be associated with minor side effects such as mild and usually short-lived psychological or physical distress associated with difficult emotions or thoughts or staying in one position (e.g., during meditation) for a longer period of time. The risk of loss of confidentiality is always of concern. Possible risks from loss of confidentially might include legal, insurance, and employment issues. Precautions to protect subject confidentiality will be implemented (see item 4 below). If certain questions or assessment tasks cause discomfort (e.g., videorecordings or sensitive questions about opioid medication use or mental health), participants will be informed they can choose not to perform these tasks or answer these questions, and encouraged to discuss any potential concerns with the research team members.

4. Adequacy of Protection Against Risks

4.a. Recruitment and Informed Consent Subjects will be recruited using several mechanisms, detailed in D.6.a. Briefly, prospective participants will be identified through the EHR database search for appropriate patients; letters, signed by the patient's regular provider or representative (e.g., medical director) and inviting participation will be mailed. Patients who are not interested will be able to send back enclosed 'opt-out' cards. Those who do not return these cards will be contacted by the study coordinator. Clinicians will be able to refer their patients to the study by providing patients with study flyers or by forwarding patient name and phone number to the study coordinator after the patient's written permission is obtained. The clinicians, clinic staff or research staff will not receive any incentives for subject recruitment. Subjects will be also able to self-refer using contact information in the flyer or pamphlet displayed at the clinics or in the community or information from the study website. We will consider other strategies (e.g., newspaper ads; study cards displayed inside the city buses) if needed. These methods were pilot-tested.[30,54-56] All clinician-investigators will help facilitate recruitment. Study coordinator will contact interested adults and conduct eligibility screen by phone. During this phone conversation, the coordinator will read an informational statement about the study and about the risks and

rights as a participant, in particular the right to cease participation at any time without any repercussion or loss of benefits. The coordinator will then seek to obtain verbal consent to proceed with eligibility screening (see D.4). Those ineligible or uninterested will be offered information about local courses. Interested eligible persons will be invited to a virtual or in-person (when available) meeting with the study coordinator to go over the details of study participation, review and complete informed consent and HIPAA procedures. Subjects will be informed that their participation is voluntary, of their right to withdraw at any time for any reason, and will be encouraged to ask questions at any time.

4.b Protections Against Risk The investigators have taken multiple steps, tested in prior trials, to protect participants against potential risks. Although no significant side effects or adverse events are anticipated, participants will be asked and encouraged to report any potential adverse events at each contact / assessment. Study personnel will directly inquire about it during each contact, for example using the following question: “Do you think that you have had any side effects or problems because of your study participation?” [If 'Yes'] “Please tell me more about it.” Data collection will also ask about side effects or adverse events during each assessment. Information on potential side effects or adverse events will be made available to the site PI who will decide if the subject requires further assessment and/or management. Standardized UW reporting forms will be used for monitoring and reporting of adverse events. All study personnel will be trained, per study protocol, in safety precautions. The therapists delivering the interventions will be mental health professionals, thus well-equipped to address any potential psychological discomfort in relation to the interventions. Pain flares are common in this population; participants will be encouraged to implement the study-taught techniques for pain coping and consult their regular physician, if needed, for additional pain care. In case of worrisome symptoms observed during the intervention sessions or assessment meetings, the therapist or a research staff will contact the designated study clinician for consultation. All in-person study meetings will take place at the medical or research facilities of collaborating institutions, with easy access to a telephone, and virtual meetings in a private location. All clinician-investigators are experienced in triaging mental health problems, including the risk of suicidality; and all physician-investigators are experienced in the management of CLBP and opioid therapy. A designated on-call clinician will be readily available via cell phone or pager. Participants will remain on-site until cleared by the clinician. Depending on clinical assessment, participants will be cleared to go home (by themselves, with family members/friends or via study-provided cab) or referred to the appropriate Emergency Department (ED). Participants who are sent home will be encouraged to contact their regular providers for evaluation, if needed, and receive a follow-up phone call the following day. In case of emergency, the therapist or research staff will call 911, and the participant’s emergency contact person will be notified (permission for this procedure will be obtained upon enrollment and signed in the HIPAA form). Study staff will have emergency contact information available for the subjects participating in a given meeting. All efforts will be made to protect subject confidentiality. All study activities will be conducted in private settings. During the intervention sessions, participants will use their first names or nicknames, and be asked to keep the information shared by others confidential. All of the data related to study participation will be kept confidential. Data will be coded with a unique code for each subject and stripped of identifiers prior to being viewed by Investigators. Electronic outcome data will be managed using the secure REDCap database. Experienced UW EHR data analyst will help link the EHR based data on prescribed opioids to other outcome data in a way that does not compromise subject confidentiality, using a study ID number for all data linkages. A master list with identifiers will be kept under lock and key by the PI or research coordinator at the UW DFMCH Research Division offices. Identifying information will not be recorded on any outcome measures/data. All patient identifying data will be destroyed after the trial is completed, per IRB guidance. We will obtain a Certificate of Confidentiality from the National Institutes of Health to additionally protect confidentiality.

5. Potential Benefits and Importance of the Knowledge to Be Gained The most salient benefit of the proposed research may be to the community and society-at-large if MM proves an effective therapy for opioid-treated CLBP that has high local, national, and global burden and for which existing strategies are unsatisfactory. Existing data suggest the safety and effectiveness of MM for improving health and reducing opioid use adults with opioid-treated CLBP. In contrast, existing pharmacotherapies (including opioid therapy) have numerous side effects. MM and CBT are used in clinical settings as adjunctive therapies for chronic pain, mental health problems and addictive disorders, but evidence on their efficacy, especially for MM, is very limited in adults with opioid-treated CLBP. If positive effects are found in the proposed study, they would suggest the proposed MM intervention could be added to the repertoire of treatment modalities for opioid-treated CLBP and contribute to the scientific knowledge base needed for informing decisions of clinicians and patients about the choice of therapy for each patient. This could help improve treatment outcomes in this population and, ultimately, result in the partial alleviation of the individual and societal burden associated with CLBP and opioid-related adverse effects. On an individual level, subjects may experience added benefit should a given study intervention have a positive effect on their CLBP-related outcomes. Overall, the potential benefits of this research far outweigh possible risks associated with study participation to both the individual participant and society.

6. Data and Safety Monitoring Plan Prior to the subject enrollment, a detailed data/safety monitoring plan will be submitted to the IRB and the PCORI for approval. Although we do not anticipate serious adverse events (see item 3), should any occur, the PI will report it to the Research Subjects Advocate and the IRB and the Project Officer at the PCORI within 48 hours, using the subject ID number. Annual reports of adverse events will be submitted to the PCORI. The data and safety monitoring will be provided through the regular study team meetings and by the Data and Safety Monitoring Committee (DSMC).

Study Team Meetings The meetings of local investigators and research staff will be led by the site PI; the site's on-average weekly research staff meetings will be led by the site manager. The Investigator Meetings, led by the PI or study manager, will be held on average every 1-2 months and include the PI, co-investigators from each site, the study manager and site managers; other team members will be able to join in as well. During all meetings, the team will discuss study progress, emerging issues, recruitment, enrollment, safety, retention, and other aspects of the study with the goal to monitor participant safety and flow, and the project timeline. Representatives from each site will meet in person up to 2 times/year on average with the members of the other team. All meetings will follow written agenda; after each meeting, meeting minutes will be summarized in writing and sent to the site PI for approval and to all team members.

Data and Safety Monitoring Committee The PI's prior RCTs evaluating MM intervention for opioid-treated CLBP and for alcohol dependence were both considered to be of "minimal risk" by the UW IRB.[30,54-56] Therefore, an independent Data and Safety Monitoring Board and an interim data analysis are not expected to be required. However, to maximize participant safety, and the validity and integrity of the data, we will form a DSMC, which we successfully implemented in our prior similar trials.[30,54-56] This committee will include 4 individuals who are not involved in the trial: two clinicians, a patient advocate (member of our study Patient/Family Advisors group), and a statistician; one of the DSMC members will chair the committee. During the course of the study, the DSMC will meet twice a year for data and safety monitoring, and to approve continuation of the study protocol. Study manager, jointly with the PI, and in coordination with the other site PI and site managers, will prepare a written report for the DSMC members to review during each

meeting; meeting minutes, with updated recommendations by the DSMC, will be sent back to the DSMC chair for approval, and communicated to investigators and managers of all sites.

7. *ClinicalTrials.gov* Requirements We will register our clinical trial in ClinicalTrails.gov prior to subject enrollment, and will comply with all ClinicalTrails.gov requirements. The research team is experienced with ClinicalTrails.gov registration and requirements; the PI's ongoing RCTs are registered and in compliance with these requirements.[30,54]

REFERENCES CITED

1. Relieving Pain in America: A Blueprint for Transforming Prevention, Care, Education, and Research. Institute of Medicine (IOM) of the National Academies; Jun 2011. Accessed Jun 28, 2016 at: http://www.iom.edu/Reports/2011/Relieving-Pain-in-America-A-Blueprint-for-Transforming-Prevention-Care-Education-Research/Report-Brief.aspx.

2. US Department of Health and Human Services. Healthy People 2020, 2020 Topics and Objectives: Arthritis, Osteoporosis, and Chronic Back Conditions. Updated 2016. Accessed Jan 20, 2016 at: http://www.healthypeople.gov/2020/topicsobjectives2020/overview.aspx?topicid=3.

3. Park PW, Dryer RD, Hegeman-Dingle R, et al. Cost Burden of Chronic Pain Patients in a Large Integrated Delivery System in the United States. *Pain Pract.* Oct 7 2015:doi: 10.1111/papr.12357. [Epub ahead of print].

4. Chou R, Deyo R, Devine B, et al. The Effectiveness and Risks of Long-Term Opioid Treatment of Chronic Pain. Evidence Report/Technology Assessment No. 218. (Prepared by the Pacific Northwest Evidence-based Practice Center under Contract No. 290-2012-00014-I.) AHRQ Publication No. 14-E005-EF. Rockville, MD: Agency for Healthcare Research and Quality; Sep 2014. Accessed Feb 10, 2016 at: http://www.effectivehealthcare.ahrq.gov/ehc/products/557/1971/chronic-pain-opioid-treatment-report-141205.pdf.

5. Gomes T, Mamdani MM, Dhalla IA, Paterson JM, Juurlink DN. Opioid dose and drug-related mortality in patients with nonmalignant pain. *Arch Intern Med.* Apr 11 2011;171(7):686-691.

6. Chou R, Fanciullo GJ, Fine PG, et al. Clinical guidelines for the use of chronic opioid therapy in chronic noncancer pain. *J Pain.* Feb 2009;10(2):113-130.

7. Dillie KS, Fleming MF, Mundt MP, French MT. Quality of life associated with daily opioid therapy in a primary care chronic pain sample. *J Am Board Fam Med.* Mar-Apr 2008;21(2):108-117.

8. The Interagency Pain Research Coordinating Committee, National Institutes of Health (NIH). National Pain Strategy: A Comprehensive Population Health Level Strategy for Pain. Apr 2015. Accessed Jan 29, 2016 at: http://iprcc.nih.gov/National_Pain_Strategy/NPS_Main.htm.

9. Patient-Centered Outcomes Research Institute (PCORI). Clinical Strategies for Managing and Reducing Long-Term Opioid Use for Chronic Pain - Cycle 3 2015 PFA. October 2015. Accessed Jan 29, 2016 at: http://www.pcori.org/funding-opportunities/announcement/clinical-strategies-managing-and-reducing-long-term-opioid-use.

10. National Center for Complementary and Alternative Medicine. Third Strategic Plan 2011-2015: Exploring the Science of Complementary and Alternative Medicine. Feb 2011. Accessed Jan 20, 2016 at: http://nccam.nih.gov/sites/nccam.nih.gov/files/NCCAM_SP_508.pdf.

11. Goyal M, Singh S, Sibinga EMS, et al. Meditation Programs for Psychological Stress and Well-Being. Comparative Effectiveness Review No. 124. (Prepared by Johns Hopkins University Evidence-based Practice Center under Contract No. 290-2007-10061–I.) AHRQ Publication No. 13(14)-EHC116-EF. Rockville, MD: Agency for Healthcare Research and Quality; Jan 2014. Accessed Apr 12, 2016 at: http://www.effectivehealthcare.ahrq.gov/ehc/products/375/1830/Meditation-report-140110.pdf.

12. Patil SG. Effectiveness of mindfulness meditation (Vipassana) in the management of chronic low back pain. *Indian J Anaesth.* Apr 2009;53(2):158-163.

13. Cramer H, Haller H, Lauche R, Dobos G. Mindfulness-based stress reduction for low back pain. A systematic review. *BMC Complement Altern Med*. 2012;12:162. doi: 10.1186/1472-6882-12-162.

14. Miller JJ, Fletcher K, Kabat-Zinn J. Three-year follow-up and clinical implications of a mindfulness meditation-based stress reduction intervention in the treatment of anxiety disorders. *Gen Hosp Psychiatry.* 1995;17(3):192-200.

15. Kabat-Zinn J, Lipworth L, Burney R, Sellers W. Four-year follow-up of a meditation-based program for the self-regulation of chronic pain: Treatment outcomes and compliance. *Clin J Pain.* 1987;2:159-173.

16. Kabat-Zinn J. *Full Catastrophe Living: Using the Wisdom of Your Body and Mind to Face Stress, Pain, and Illness*. New York: Delta; 1990.

17. Garland EL, Howard MO. Mindfulness-oriented recovery enhancement reduces pain attentional bias in chronic pain patients. *Psychother Psychosom.* 2013;82(5):311-318.

18. Kutz I, Borysenko JZ, Benson H. Meditation and psychotherapy: a rationale for the integration of dynamic psychotherapy, the relaxation response, and mindfulness meditation. *Am J Psychiatry.* 1985;142(1):1-8.

19. McCracken LM, Vowles KE. Acceptance and commitment therapy and mindfulness for chronic pain: model, process, and progress. *Am Psychol.* Feb-Mar 2014;69(2):178-187.

20. van Middelkoop M, Rubinstein SM, Kuijpers T, et al. A systematic review on the effectiveness of physical and rehabilitation interventions for chronic non-specific low back pain. *Eur Spine J.* Jan 2011;20(1):19-39.

21. Henschke N, Ostelo RW, van Tulder MW, et al. Behavioural treatment for chronic low-back pain. *Cochrane Database Syst Rev.* 2010;(7):CD002014. doi: 10.1002/14651858.CD002014.pub3.

22. Williams AC, Eccleston C, Morley S. Psychological therapies for the management of chronic pain (excluding headache) in adults. *Cochrane Database Syst Rev*. 2012;11:CD007407. doi: 10.1002/14651858.CD007407.pub3.

23. Otis J. *Managing Chronic Pain: A Cognitive-Behavioral Therapy Approach Therapist Guide*. New York: Oxford University Press; 2007.

24. Otis J. *Managing Chronic Pain: A Cognitive-Behavioral Therapy Approach Workbook*. New York: Oxford University Press; 2007.

25. Jamison RN. Mastering Chronic Pain: A Professional's Guide to Behavioral Treatment. Sarasota, FL: Professional Resource Press; 1996.

26. National Center for Complementary and Integrative Health, U.S. Department of Health and Human Services, National Institutes of Health (NIH). Get the Facts: Meditation: What You Need To Know. NCCIH Pub No: D308, Created: Dec 2007; Updated: Nov 2014. Accessed Jan 28, 2016 at: https://nccih.nih.gov/sites/nccam.nih.gov/files/Get_The_Facts_Meditation_12-18-2014.pdf.

27. Patient-Centered Outcomes Research Institute (PCORI). Prioritizing Comparative Effectiveness Research Questions for the Long-Term Use of Opioids for Chronic Pain: A Stakeholder Workshop on Multimodal Treatment Options, Risk Mitigation Strategies, and Opioid Dependency Meeting Summary. June 2015. Accessed Jan 20, 2016 at: http://www.pcori.org/sites/default/files/PCORI-Prioritizing-CER-Questions-Stakeholder-Workshop-Opioids-Pharmacologic-Meeting-Summary-Group-2-060915.pdf.

28. Chiesa A, Serretti A. Mindfulness-based interventions for chronic pain: a systematic review of the evidence. *J Altern Complement Med.* Jan 2011;17(1):83-93.

29. Institute of Medicine of the National Academies Committee on Comparative Effectiveness Research Prioritization Board on Health Care Services. Initial National Priorities for Comparative Effectiveness Research. June 30, 2009. Accessed Jan 20, 2016 at: http://www.iom.edu/Reports/2009/ComparativeEffectivenessResearchPriorities.aspx.

30. Zgierska A, Burzinski CA, Cox J, et al. Mindfulness Meditation and Cognitive Behavioral Therapy Intervention Reduces Pain Severity and Sensitivity in Opioid-Treated Chronic Low Back Pain: Pilot Findings from a Randomized Controlled Trial. Clinicaltrials.gov # NCT01775995. Accepted, Pain Medicine 2015.

31. Garland EL, Manusov EG, Froeliger B, Kelly A, Williams JM, Howard MO. Mindfulness-Oriented Recovery Enhancement for Chronic Pain and Prescription Opioid Misuse: Results From an Early-Stage Randomized Controlled Trial. *J Consult Clin Psychol.* 2014;82(3):448-459.

32. Windmill J, Fisher E, Eccleston C, et al. Interventions for the reduction of prescribed opioid use in chronic non-cancer pain. *Cochrane Database Syst Rev.* 2013;9:CD010323. doi: 10.1002/14651858.CD010323.pub2.

33. Cherkin DC, Sherman KJ, Balderson BH, et al. Effect of Mindfulness-Based Stress Reduction vs Cognitive Behavioral Therapy or Usual Care on Back Pain and Functional Limitations in Adults With Chronic Low Back Pain: A Randomized Clinical Trial. *JAMA.* Mar 22-29 2016;315(12):1240-1249.

34. Davis MC, Zautra AJ, Wolf LD, Tennen H, Yeung EW. Mindfulness and cognitive-behavioral interventions for chronic pain: differential effects on daily pain reactivity and stress reactivity. *J Consult Clin Psychol.* Feb 2015;83(1):24-35.

35. Zautra AJ, Davis MC, Reich JW, et al. Comparison of cognitive behavioral and mindfulness meditation interventions on adaptation to rheumatoid arthritis for patients with and without history of recurrent depression. *J Consult Clin Psychol.* Jun 2008;76(3):408-421.

36. Teper R, Segal ZV, Inzlicht M. Inside the Mindful Mind: How Mindfulness Enhances Emotion Regulation Through Improvements in Executive Control. *Curr Dir Psychol Sci.* Dec 2013;22(6):449-454.

37. Sheppes G, Scheibe S, Suri G, Gross JJ. Emotion-regulation choice. *Psychological science.* Nov 2011;22(11):1391-1396.

38. Raio CM, Orederu TA, Palazzolo L, Shurick AA, Phelps EA. Cognitive emotion regulation fails the stress test. *Proc Natl Acad Sci U S A.* Sep 10 2013;110(37):15139-15144.

39. Jamison RN, Edwards RR, Liu X, et al. Relationship of negative affect and outcome of an opioid therapy trial among low back pain patients. *Pain Pract.* Mar 2013;13(3):173-181.

40. Martel MO, Dolman AJ, Edwards RR, Jamison RN, Wasan AD. The Association Between Negative Affect and Prescription Opioid Misuse in Patients With Chronic Pain: The Mediating Role of Opioid Craving. *Journal of Pain.* Jan 2014;15(1):90-100.

41. Wasan AD, Michna E, Edwards RR, et al. Psychiatric Comorbidity Is Associated Prospectively with Diminished Opioid Analgesia and Increased Opioid Misuse in Patients with Chronic Low Back Pain. *Anesthesiology.* Oct 2015;123(4):861-872.

42. Zgierska A, Burzinski CA, Cox J, et al. Feasibility, acceptability and safety of mindfulness meditation and cognitive behavioral therapy intervention for opioid-treated chronic low back pain. Clinicaltrials.gov # NCT01775995. Accepted, Journal of Alternative and Complementary Medicine, 2016.

43. Matthias MS, Parpart AL, Nyland KA, et al. The patient-provider relationship in chronic pain care: providers' perspectives. *Pain Med.* Nov 2010;11(11):1688-1697.

44. Zgierska A, Miller M, Rabago D. Patient satisfaction, prescription drug abuse, and potential unintended consequences. *JAMA.* Apr 2012;307(13):1377-1378.

45. Zgierska A, Rabago D, Miller MM. Impact of patient satisfaction ratings on physicians and clinical care. *Patient Prefer Adherence.* 2014;8:437-446.

46. Jamison RN, Sheehan KA, Scanlan E, Matthews M, Ross EL. Beliefs and attitudes about opioid prescribing and chronic pain management: survey of primary care providers. *J Opioid Manag.* Nov-Dec 2014;10(6):375-382.

47. Brooks EA, Unruh A, Lynch ME. Exploring the lived experience of adults using prescription opioids to manage chronic noncancer pain. *Pain Res Manag.* Jan-Feb 2015;20(1):15-22.

48. Ostelo RW, Deyo RA, Stratford P, et al. Interpreting change scores for pain and functional status in low back pain: towards international consensus regarding minimal important change. *Spine (Phila Pa 1976).* Jan 1 2008;33(1):90-94.

49. Dworkin RH, Turk DC, Wyrwich KW, et al. Interpreting the clinical importance of treatment outcomes in chronic pain clinical trials: IMMPACT recommendations. *J Pain.* Feb 2008;9(2):105-121.

50. Hagg O, Fritzell P, Nordwall A. The clinical importance of changes in outcome scores after treatment for chronic low back pain. *Eur Spine J.* Feb 2003;12(1):12-20.

51. Zgierska A, Wallace ML, Burzinski CA, Cox J, Backonja M. Pharmacological and toxicological profile of opioid-treated, chronic low back pain patients entering a mindfulness intervention randomized controlled trial. *J Opioid Manag.* Sep-Oct 2014;10(5):323-335.

52. Holzer J, Kass N. Community Engagement Strategies in the Original and Renewal Applications for CTSA Grant Funding. *Clin Transl Sci.* Feb 2014;7(1):38-43.

53. Zgierska A, Tuan WJ. Opioid-treated chronic low back pain in adult outpatients. UW Health Epic-Clarity Relational Database Management System, outpatient data Jan-Dec 2015.

54. Zgierska A. UW Madison. Mindfulness Meditation for Alcohol Relapse Prevention. NIH NIAAA, 2009-2014. K23 Career Development Award; $943,043. K23 AA017508. Accessed Nov 10, 2015 at: http://www.clinicaltrials.gov/ct2/show/NCT01056484?term=zgierska&rank=1.

55. Barrett B, Hayney MS, Muller D, et al. Meditation or Exercise for Preventing Acute Respiratory Infection: A Randomized Controlled Trial. *Ann Fam Med.* Jul 2012;10(4):337-346.

56. Barrett. B. UW Madison. University of Wisconsin Meditation & Exercise Cold Study (MEPARI-2). NIH NCCAM, 2012-2016. R01 Award; $751,529. AT006970. Available at: http://www.clinicaltrials.gov/ct2/show/NCT01654289?term=meditation+exercise+acute+respiratory&rank=2: UW Madison.

57. Jamison RN, Ross EL, Michna E, Chen LQ, Holcomb C, Wasan AD. Substance misuse treatment for high-risk chronic pain patients on opioid therapy: a randomized trial. *Pain.* Sep 2010;150(3):390-400.

58. Patient-Centered Outcomes Research Institute (PCORI). Research Methodology. Posted Sep 2013; Updated Apr 2014. Accessed Jan 29, 2016 at: http://www.pcori.org/research-results/research-methodology.

59. Thorpe KE, Zwarenstein M, Oxman AD, et al. A pragmatic-explanatory continuum indicator summary (PRECIS): a tool to help trial designers. *J Clin Epidemiol.* May 2009;62(5):464-475.

60. Chapman JR, Norvell DC, Hermsmeyer JT, et al. Evaluating common outcomes for measuring treatment success for chronic low back pain. *Spine (Phila Pa 1976).* Oct 1 2011;36(21 Suppl):S54-68.

61. Deyo RA, Dworkin SF, Amtmann D, et al. Report of the NIH Task Force on Research Standards for Chronic Low Back Pain. *International journal of therapeutic massage & bodywork.* Sep 2015;8(3):16-33.

62. Dworkin RH, Turk DC, Farrar JT, et al. Core outcome measures for chronic pain clinical trials: IMMPACT recommendations. *Pain.* Jan 2005;113(1-2):9-19.

63. Patient-Centered Outcomes Research Institute (PCORI). PCORI Application Guidelines: The National Patient-Centered Clinical Research Network (PCORnet): Initiative on Health Plan/System Data Partnerships (A Stepwise Approach to Collaboration). Published Oct 2015; Updated Nov 2015. Accessed Jan 29, 2016 at: http://www.pcori.org/sites/default/files/PCORI-PFA-2015-Health-Plans-Application-Guidelines.pdf.

64. Pannucci CJ, Wilkins EG. Identifying and Avoiding Bias in Research. *Plast Reconstr Surg.* Aug 2010;126(2):619-625.

65. Schulz KF, Altman DG, Moher D. CONSORT 2010 Statement: Updated guidelines for reporting parallel group randomised trials. *J Clin Epidemiol.* Aug 2010;63(8):834-840.

66. Fairbank JC, Pynsent PB. The Oswestry Disability Index. *Spine.* Nov 15 2000;25(22):2940-2953.

67. Dunn KM, Saunders KW, Rutter CM, et al. Opioid prescriptions for chronic pain and overdose: a cohort study. *Ann Intern Med.* Jan 2010;152(2):85-92.

68. Bowen S, Chawla N, Marlatt A. *Mindfulness-Based Relapse Prevention for Addictive Behaviors: A Clinician's Guide.* New York: Guilford Press; 2010.

69. Segal ZV, Williams JM, Teasdale JD. Mindfulness-Based Cognitive Therapy for Depression. Second Edition. New York: Guilford Press; 2013.

70. Zgierska A, Rabago D, Zuelsdorff M, Coe C, Miller M, Fleming M. Mindfulness meditation for alcohol relapse prevention: a feasibility pilot study. *J Addict Med.* Sep 2008;2(3):165-173.

71. Gatchel RJ, Rollings KH. Evidence-informed management of chronic low back pain with cognitive behavioral therapy. *Spine J.* Jan-Feb 2008;8(1):40-44.

72. S S. Mindfulness-Based Stress Reduction (MBSR): Standards of practice. The Center for Mindfulness in Medicine, Health Care and Society. University of Massachusettes Medical School. 2001. Revised February 2014. Accessed

on Feb 7, 2016 at: https://www.umassmed.edu/contentassets/24cd221488584125835e2eddce7dbb89/mbsr_standards_of_practice_2014.pdf.
73. Kabat-Zinn J. An outpatient program in behavioral medicine for chronic pain patients based on the practice of mindfulness meditation: theoretical considerations and preliminary results. *Gen Hosp Psychiatry.* 1982;4(1):33-47.
74. Kabat-Zinn J, Lipworth L, Burney R. The clinical use of mindfulness meditation for the self-regulation of chronic pain. *J Behav Med.* 1985;8(2):163-190.
75. Kabat-Zinn J, Chapman-Waldrop A. Compliance with an outpatient stress reduction program: rates and predictors of program completion. *J Behav Med.* 1988;11(4):333-352.
76. Ellis A. Rational Psychotherapy and Individual Psychology. *J Indiv Psychol.* 1957;13(1):38-44.
77. Bellg AJ, Borrelli B, Resnick B, et al. Enhancing treatment fidelity in health behavior change studies: best practices and recommendations from the NIH Behavior Change Consortium. *Health Psychol.* Sep 2004;23(5):443-451.
78. Borrelli B, Sepinwall D, Ernst D, et al. A new tool to assess treatment fidelity and evaluation of treatment fidelity across 10 years of health behavior research. *J Consult Clin Psychol.* Oct 2005;73(5):852-860.
79. Chawla N, Collin S, Bowen S, et al. The mindfulness-based relapse prevention adherence and competence scale: development, interrater reliability, and validity. *Psychother Res.* Jul 2010;20(4):388-397.
80. Deyo RA, Smith DH, Johnson ES, et al. Opioids for back pain patients: primary care prescribing patterns and use of services. *J Am Board Fam Med.* Nov-Dec 2011;24(6):717-727.
81. Keller S, Bann CM, Dodd SL, Schein J, Mendoza TR, Cleeland CS. Validity of the brief pain inventory for use in documenting the outcomes of patients with noncancer pain. *Clin J Pain.* Sep-Oct 2004;20(5):309-318.
82. Von Korff M, Jensen MP, Karoly P. Assessing global pain severity by self-report in clinical and health services research. *Spine (Phila Pa 1976).* Dec 15 2000;25(24):3140-3151.
83. Tan G, Jensen MP, Thornby JI, Shanti BF. Validation of the Brief Pain Inventory for chronic nonmalignant pain. *J Pain.* Mar 2004;5(2):133-137.
84. Fairbank JC, Couper J, Davies JB, O'Brien JP. The Oswestry low back pain disability questionnaire. *Physiotherapy.* Aug 1980;66(8):271-273.
85. Savre I, Fairbank J. ODI Oswestry Disability Index Information Booklet. 1st edn. France: Mapi Research Trust; 2011.
86. Luo X, George ML, Kakouras I, et al. Reliability, validity, and responsiveness of the short form 12-item survey (SF-12) in patients with back pain. *Spine (Phila Pa 1976).* Aug 1 2003;28(15):1739-1745.
87. Ware J, Jr., Kosinski M, Keller SD. A 12-Item Short-Form Health Survey: construction of scales and preliminary tests of reliability and validity. *Med Care.* Mar 1996;34(3):220-233.
88. Sobell LC, Brown J, Leo GI, Sobell MB. The reliability of the Alcohol Timeline Followback when administered by telephone and by computer. *Drug Alcohol Depend.* 1996;42(1):49-54.
89. Fals-Stewart W, O'Farrell TJ, Freitas TT, McFarlin SK, Rutigliano P. The timeline followback reports of psychoactive substance use by drug-abusing patients: psychometric properties. *J Consult Clin Psychol.* 2000;68(1):134-144.
90. Sobell LC, Sobell MB. Timeline Followback: a technique for assessing self-reported alcohol consumption. In: Litten RZ, Allen J, eds. Measuring Alcohol Consumption: Psychosocial and Biological Methods. New Jersey: Humana Press, 1992.
91. Zigmond AS, Snaith RP. The hospital anxiety and depression scale. *Acta Psychiatr Scand.* Jun 1983;67(6):361-370.
92. Bjelland I, Dahl AA, Haug TT, Neckelmann D. The validity of the Hospital Anxiety and Depression Scale. An updated literature review. *J Psychosom Res.* Feb 2002;52(2):69-77.

93. Butler SF, Budman SH, Fanciullo GJ, Jamison RN. Cross validation of the current opioid misuse measure to monitor chronic pain patients on opioid therapy. *Clin J Pain.* Nov-Dec 2010;26(9):770-776.

94. Jamison RN, Martel MO, Edwards RR, Qian J, Sheehan KA, Ross EL. Validation of a brief Opioid Compliance Checklist for patients with chronic pain. *J Pain.* Nov 2014;15(11):1092-1101.

95. Jamison RN, Martel MO, Huang C, Jurcik D, Edwards RE. Efficacy of the Opioid Compliance Checklist to Monitor Chronic Pain Patients Receiving Opioid Therapy in Primary Care. Accepted, 2016, J Pain.

96. de Boer MJ, Steinhagen HE, Versteegen GJ, Struys MM, Sanderman R. Mindfulness, acceptance and catastrophizing in chronic pain. *PLoS One.* 2014;9(1):e87445.

97. Vowles KE, McCracken LM, Eccleston C. Processes of change in treatment for chronic pain: the contributions of pain, acceptance, and catastrophizing. *Eur J Pain.* Oct 2007;11(7):779-787.

98. Osman A, Barrios FX, Kopper BA, Hauptmann W, Jones J, O'Neill E. Factor structure, reliability, and validity of the pain catastrophizing scale. *Journal of Behavioral Medicine.* Dec 1997;20(6):589-605.

99. McCracken LM, Vowles KE, Eccleston C. Acceptance of chronic pain: component analysis and a revised assessment method. *Pain.* Jan 2004;107(1-2):159-166.

100. Brown KW, Ryan RM. The benefits of being present: mindfulness and its role in psychological well-being. *J Pers Soc Psychol.* 2003;84(4):822-848.

101. Zgierska A, Brown R, Zuelsdorff M, Brown D, Zhang Z, Fleming M. Sleep and daytime sleepiness problems among chronic non-cancerous pain patients receiving long-term opioid therapy: a cross-sectional study. *J Opioid Manage.* Nov-Dec 2007;3(6):317-327.

102. Cohen SP, Mao J. Neuropathic pain: mechanisms and their clinical implications. *BMJ.* 2014;348:f7656.

103. Freynhagen R, Baron R, Gockel U, Tolle TR. painDETECT: a new screening questionnaire to identify neuropathic components in patients with back pain. *Curr Med Res Opin.* Oct 2006;22(10):1911-1920.

104. Guy W. ECDEU assessment manual for psychopharmacology (DHEW Publication No. ADM 76-338). Washington, DC, U.S. Government Printing Office, 1976.

105. Gossop M. The development of a Short Opiate Withdrawal Scale (SOWS). *Addict Behav.* 1990;15(5):487-490.

106. Vernon MK, Reinders S, Mannix S, Gullo K, Gorodetzky CW, Clinch T. Psychometric evaluation of the 10-item Short Opiate Withdrawal Scale-Gossop (SOWS-Gossop) in patients undergoing opioid detoxification. *Addict Behav.* Sep 2016;60:109-116.

107. Rich M, Lamola S, Gordon J, Chalfen R. Video intervention/prevention assessment: a patient-centered methodology for understanding the adolescent illness experience. *J Adolesc Health.* Sep 2000;27(3):155-165.

108. Henry SG, Fetters MD. Video Elicitation Interviews: A Qualitative Research Method for Investigating Physician-Patient Interactions. *Annals of Family Medicine.* Mar-Apr 2012;10(2):118-125.

109. Bate P, Robert G. Experience-based design: from redesigning the system around the patient to co-designing services with the patient. *Qual Saf Health Care.* Oct 2006;15(5):307-310.

110. Mathias S, Nayak USL, Isaacs B. Balance in Elderly Patients - the Get-up and Go Test. *Arch Phys Med Rehab.* Jun 1986;67(6):387-389.

111. Podsiadlo D, Richardson S. The Timed up and Go - a Test of Basic Functional Mobility for Frail Elderly Persons. *Journal of the American Geriatrics Society.* Feb 1991;39(2):142-148.

112. Halverson E, Bass M, Woods D. The Process of Creation: A Novel Methodology for Analyzing Multimodal Data. The Qualitative Report, 2012, 17, Article 21, 1-27. Available at: http://www.nova.edu/ssss/QR/QR17/halverson.pdf.

113. Woods D, Dempster P. Tales from the Bleeding Edge: The Qualitative Analysis of Complex Video Data Using Transana. Forum Qualitative Sozialforschung / Forum: Qualitative Social Research, 2011, 12(1): Art 17. Available at: http://www.qualitative-research.net/index.php/fqs/article/view/1516.

114. Woods D, Fassnacht C. Transana v3.00 [Computer software]. The Board of Regents of the University of Wisconsin System, Madison, Wisconsin, 2015. Accessed on 2-7-16 at: www.transana.org.

115. Butler SF, Budman SH, Fernandez KC, et al. Development and validation of the Current Opioid Misuse Measure. *Pain.* Jul 2007;130(1-2):144-156.

116. Beunckens C, Molenberghs G, Kenward MG. Direct likelihood analysis versus simple forms of imputation for missing data in randomized clinical trials. *Clin Trials.* 2005;2(5):379-386.

117. White IR, Royston P, Wood AM. Multiple imputation using chained equations: Issues and guidance for practice. *Stat Med.* Feb 20 2011;30(4):377-399.

118. Schafer JL. Analysis of incomplete multivariate data. London; New York: Chapman & Hall; 1997.

119. Carpenter JR, Kenward MG, White IR. Sensitivity analysis after multiple imputation under missing at random: a weighting approach. *Stat Methods Med Res.* Jun 2007;16(3):259-275.

120. Kloke JD, Mckean JW, Rashid MM. Rank-Based Estimation and Associated Inferences for Linear Models With Cluster Correlated Errors. *Journal of the American Statistical Association.* Mar 2009;104(485):384-390.

121. NVivo Qualitative Data Analysis Software. QSR International Pty Ltd. Version 10, 2012.

122. Leon AC, Heo M. Sample Sizes Required to Detect Interactions between Two Binary Fixed-Effects in a Mixed-Effects Linear Regression Model. *Computational statistics & data analysis.* Jan 15 2009;53(3):603-608.

123. Garland EL, Roberts-Lewis A, Tronnier CD, Graves R, Kelley K. Mindfulness-Oriented Recovery Enhancement versus CBT for co-occurring substance dependence, traumatic stress, and psychiatric disorders: Proximal outcomes from a pragmatic randomized trial. *Behav Res Ther.* Feb 2016;77:7-16.

124. Garland EL, Froeliger B, Howard MO. Effects of Mindfulness-Oriented Recovery Enhancement on reward responsiveness and opioid cue-reactivity. *Psychopharmacology (Berl).* Aug 2014;231(16):3229-3238.

125. Day MA, Jensen MP, Ehde DM, Thorn BE. Toward a Theoretical Model for Mindfulness-Based Pain Management. *Journal of Pain.* Jul 2014;15(7):691-703.

126. Patient-Centered Outcomes Research Institute (PCORI). The National Patient-Centered Clinical Research Network (PCORnet). Common Data Model (CDM) Specification, Version 3.0. Accessed Feb 3, 2016 at: http://www.pcornet.org/wp-content/uploads/2014/07/2015-07-29-PCORnet-Common-Data-Model-v3dot0-RELEASE.pdf.

127. FDA. Non-inferiority clinical trials to establish effectiveness: guidance for industry. US: Department of Health and Human Services 2016.

128. Emanuel EJ. Committee for Proprietary Medicinal Products. Points to consider on switching between superiority and non-inferiority. Br J Clin Pharmacol 2001;52(3):223.

129. Dworkin RH, Turk DC, Wyrwich KW, et al. Interpreting the clinical importance of treatment outcomes in chronic pain clinical trials: IMMPACT recommendations. The Journal of Pain 2008;9(2):105-21.

CONSORTIUM CONTRACTUAL ARRANGEMENTS

Provide the information requested below. Do not exceed 10 pages. Detailed instructions are included in the Application Guidelines for this PFA.

This application has **three consortium partners, Pain Management Center of Brigham and Women's Hospital (BWH), Harvard Medical School in Boston, Massachusetts and the College of Social Work at University of Utah in Salt Lake City, UT,** and Pennsylvania State University College of Medicine, critical to the completion of the proposed work. Together with the University of Wisconsin, these organizations will participate collaboratively and complete the research procedures and processes at each site. This multi-site study would not be possible without these partnerships. Boston and Salt Lake City Investigators have been instrumental to the development and revision of this proposal, and, along with the resources offered by their Institutions, are crucial to the success of the proposed study. We have included a signed consortium intent.

We will work directly with Drs. Robert Edwards (Boston's site PI), Robert Jamison, Edward Michna and Edgar Ross, all faculty at the Harvard Medical School and Pain Management Center of Brigham and Women's Hospital (BWH); the BWH's Pain Management Center is directed by Dr. Ross. We will also work directly with Drs. Eric Garland (Salt Lake City's site PI), Yoshio Nakamura and Julie Fritz, all faculty at the University of Utah. Dr. Aleksandra Zgierska is the Dual PI for this proposal and the Hershey site PI and, jointly with Dr. Robert Lennon, faculty at Penn State. The Investigators are nationally-acknowledged experts in chronic pain disorders and their treatment, and in application of cognitive behavioral therapy (CBT) and Mindfulness Meditation (MM) to pain management. As such, they have and will continue providing an invaluable input on and assistance with the study design, conduct, data analysis, and result interpretation and dissemination. Drs. Edwards and Jamison, pain psychologists and researchers, will also be responsible for finalizing the manuals for CBT intervention, for training of the study therapists in its delivery, and for monitoring and enhancing the fidelity of CBT intervention delivery throughout the study. Dr. Garland, clinical social worker and researcher of MM for opioid-treated chronic pain and opioid misuse reduction, will be responsible for finalizing the manuals with the PI and Co-I Nakamura for the MM intervention, for training of the study therapists in MM intervention delivery, and for monitoring and enhancing the fidelity of MM intervention delivery throughout the study.

Addition of the Boston and Salt Lake City sites also makes the proposed comparative effectiveness study feasible, as these sites are vital to the success of efficient participant recruitment, enrollment and retention. The Boston site will be able to draw patients from two hospitals of the Partners Healthcare System: BWH and Massachusetts General Hospital, and their affiliated clinics; the Salt Lake site will draw patients from 11 University of Utah Health Care primary care clinics across the greater Salt Lake City metropolitan area and the clinics associated with the University of Utah Hospital. The Boston metropolitan area includes 4.5 million people and is an urban region with good representation of racial minorities, and the Salt Lake City metropolitan area includes 1.2 million people; therefore, enabling access to a large and very diverse participant pool that will contribute to efficient recruitment and generalizability of the study results.

Boston, Salt Lake City and Hershey site Investigators are equal team members and stakeholders in the proposed research. Their organizations will be providing the study interventions and cover the costs related to intervention delivery at their sites, Boston and Salt Lake City Investigators will be responsible for recruiting participants and

overseeing their team of research staff and data collection in their respective communities. Our Madison, Boston, and Salt Lake City teams will ensure an effective and sustainable collaboration and communication throughout the project. Representatives of the Boston and Salt Lake City investigative teams will fly to Madison up to twice per year throughout the project to participate in the study team meetings, which will include both research and stakeholder members, and will participate in the scheduled and as-needed tele-meetings of the study team. The study PI and/or the Study Manager Cindy Burzinski will attend the Boston and Salt Lake City site team meetings at least annually.

If the MM intervention is shown to be successful, our collaboration with the Hershey, Boston and Salt Lake City site Investigators and psychologists (trained in the delivery of this intervention during the study), and the presence of strong Institutional support for MM as a therapy with great potential for improving outcomes in opioid-treated CLBP, expands our options for effective future dissemination and implementation of this program in other communities throughout the US.

APPENDIX

Appendix A: Outline of the Eight-Week MM Intervention

Session	Session Concepts	Exercises / Techniques
**1. Automatic Pilot and Pain**	● Define mindfulness meditation<br>● Automatic pilot and how it impacts responses; The pain cycle<br>● Discriminate between nociception, pain, and suffering<br>● Present-moment awareness: acceptance and non-judgment	● "Raisin or Chocolate" mindfulness exercise<br>● Breath meditation<br>● Body Scan meditation<br>● SABER*Mini-Meditation
**2. Triggers and Automatic Reactions**	● Autopilot: its impact on our experiences and responses<br>● Mindfulness and pain: triggers and automatic reactions to pain<br>● Present moment awareness ('experience things as they are')	● Breath meditation<br>● Body scan<br>● SABER*Mini-Meditation
**3. Are Thoughts Facts…? Mindful Reappraisal**	● Explain automatic thoughts; relationship between thoughts and emotions and pain, automatic reactions to pain, and pain coping<br>● Learn your own automatic thought patterns associated with pain that may lead to worse coping and outcomes<br>● Mindful reappraisal - Helpful vs. unhelpful cognitions: effects of catastrophizing on pain, mood, and function	● 'Body and breath' meditation<br>● "Walking down the street"<br>● Mindful reappraisal<br>● 'Pain chain: the role of mindfulness' exercise<br>● Start 'coping cards'<br>● SABER* Mini-Meditation
**4. Mindfulness in Daily Life – Mindful Savoring**	● Breath as an anchor to the present-moment experiences<br>● Brief mindfulness practices in daily life: moment-by-moment awareness of bodily sensations, emotions and cognitions<br>● Mindful savoring – enhancing positive emotions<br>● SABER* as a way to 'pause' before reacting to stressors or pain	● Mindful savoring<br>● 'Mindfulness of breath and body' meditation<br>● Mindful walking/movement<br>● SABER* Mini-Meditation
**5. Mindfulness and Opioid Use**	● Awareness of the effects of opioids on mind and body<br>● Mindfulness of the perceived need for opioids ("urges")<br>● Contemplation of negative consequences of opioid use and positive consequences of opioid dose reduction	● 'Mindfulness of breath and body' meditation<br>● "Chocolate" mindfulness exercise with focus on mindfulness of urges and contemplation of consequences<br>● SABER* Mini-Meditation
**6. Balancing Acceptance and Skillful, Mindful Action (Change)**	● Acceptance versus non-acceptance of pain and other unwanted experiences, and their impact on outcomes<br>● Cultivating acceptance<br>● Relationship between acceptance and working toward change	● Mindful movement<br>● Sitting meditation<br>● 'Pain Wave Surfing'** exercise<br>● SABER:* Acceptance of pain
**7. Self-Care and Life Balance**	● Life balance: nourishing and depleting activities, importance of self-care; mindfulness for improving sleep and anger management<br>● Vulnerability to the automatic 'pain chain' reactions: early warning signs of vulnerability; ways to reduce vulnerability<br>● Coping behaviors: ignoring/denying, avoidance, resistance versus mindfulness<br>● Mindfulness practice as a means of maintaining lifestyle balance	● Breath meditation<br>● 'Where the pain chain begins' exercise<br>● 'Life balance' exercise<br>● Loving-kindness meditation<br>● Mindful movement<br>● Complete coping cards
**8. Balanced Living: Building Support Networks, Continuing to Live Mindfully**	● Building a support network: barriers to asking for help; importance of support networks for continued practice and healthy pain coping<br>● Participant self-reflections on the taught/learned skills<br>● Mindfulness practice as a way of maintaining balance in life	● Body Scan meditation<br>● Barriers to asking for help<br>● Helpful and challenging aspects of MM practice<br>● Next steps in study activities

	● Participant group reflections on reasons for and ways of a successful continuation of mindfulness practice in daily life	● Concluding meditation

Adapted from the curricula of the existing programs: Mindfulness-Based Stress Reduction (MBSR),[16] Mindfulness-Based Cognitive Therapy for Depression (MBCT),[69] Mindfulness-Based Relapse Prevention (MBRP),[68] and Mindfulness-Oriented Recovery Enhancement (MORE).[31]

* **SABER Mini-Meditation** (adapted from the MBRP program[68]):

STOP right here and right now; this pause can allow you to step out of your autopilot.

ACKNOWLEDGE what is happening right now, in this moment, what's really going on in your mind and body with your thoughts, sensation and emotions.

BREATHE: focus your attention on the breath.

EXPAND your awareness to include a sense of the body as a whole.

RESPOND: now, try to make a mindful choice what to do next (if anything)….

** **Pain Wave Surfing exercise** (adapted from the MBRP program[68]) encourages to "observe and accept." One visualizes pain as an ocean wave that begins small and gradually builds to a large cresting wave. Using the awareness of one's breath as a surfboard, the goal is to "surf the wave" by allowing it to first rise, and then fall without being "wiped out" by giving into it. As with a wave, the pain grows in intensity until it reaches its peak, then subsides on its own. Successfully "surfing the wave" weakens pain-related conditioning and enhances coping skills.[120] The process of incorporating meditation to accept and tolerate pain is compatible with developing a repertoire of new acceptance-based coping skills.

Appendix A: Outline of the Eight-Week CBT Intervention

Session	Session Concepts	Exercises / Techniques
**1. Education on Chronic Pain**	● Impact of chronic pain<br>● The pain cycle<br>● General goals for treatment<br>● Set overall behavioral goals for treatment	● Goal-setting exercise<br>● Group discussions<br>● 'Things that Affect My Pain' worksheet
**2. Theories of Pain and Relaxation**	● Theories of pain<br>● Relaxation methods: diaphragmatic breathing, visual imagery<br>● Non-medication management of pain flares	● Diaphragmatic breathing practice<br>● Visual Imagery practice<br>● Pain flare management exercise
**3. Stress Management in Daily Life**	● Definition of stress; sources of stress; "fight-or-flight" response<br>● Relationship between stress and pain<br>● Progressive muscle relaxation<br>● Examine ways to decrease stress in every-day life	● Diaphragmatic breathing practice<br>● Stress-Pain connection worksheet<br>● Progressive muscle relaxation
**4. Time-Based Pacing**	● Introduce time-based pacing<br>● Discuss pacing techniques, teach steps to appropriate pacing<br>● Unhealthy coping strategies<br>● Attention diversion: concepts, techniques (focus on physical surroundings; backward counting; focus on auditory stimuli)	● Time-based pacing practice<br>● 'ABC model'* exercise<br>● Time-based pacing exercise<br>● Attention diversion practices
**5. Social Support**	● The benefits of social support<br>● Supportive coping versus solicitousness<br>● Enlisting the help of others in managing pain<br>● Enhancing social skills<br>● Methods for improving assertiveness<br>● Engaging family and friends in positive/pleasant activities	● Barriers to asking for help<br>● Assertiveness versus aggressiveness worksheet<br>● Visual imagery practice<br>● Facilitating supportive coping exercise
**6. Automatic Thoughts and Pain**	● Explain automatic thoughts<br>● Relationship between thoughts and emotions and pain<br>● The ABC model*<br>● Irrational negative thoughts: cognitive errors, catastrophizing,<br>● Cognitive restructuring: developing more rational alternative thoughts (four categories: preparing, beginning, during and after)	● Progressive muscle relaxation<br>● 'Automatic Thoughts' exercise<br>● Pain catastrophizing exercise<br>● 'Restructuring Thoughts' exercise<br>● Start 'coping cards'<br>● Time-based pacing practice
**7. Self-Care and Life Balance**	● Life balance: nourishing vs. depleting activities, role of self-care<br>● Different coping behaviors<br>● Anger: define anger; relationship between anger and pain; role of different response styles in anger management<br>● Sleep Hygiene: explain the importance; ways to improve sleep	● Diaphragmatic Breathing exercise<br>● 'Life balance' exercise<br>● Visual Imagery practice<br>● 'Sleep Pattern' exercise<br>● Complete coping cards
**8. Relapse Prevention and Flare-Up Planning**	● Discuss relapse prevention and flare-up planning<br>● Stages of flare-up management<br>● Review concepts taught and skills learned<br>● How to build a support network: barriers to asking for help; role of support networks for continued practice and healthy pain coping	● Progressive muscle relaxation<br>● Feedback about helpful and challenging aspects of treatment<br>● Next steps in study activities<br>● Diaphragmatic breathing

[#] Adapted from "Managing Chronic Pain: A Cognitive-Behavioral Therapy Approach" by John D. Otis (Boston University School of Medicine; Veteran Affairs Boston Healthcare System);[23,24] and from "Mastering Chronic Pain: A Professional's Guide to Behavioral Treatment"[25] by **Co-Investigator Robert Jamison** (Harvard Medical School).

*** The ABC Model,** adapted from cognitive therapy techniques[76] is a tool to help structure one's understanding of negative thinking about pain. In this model, ***A***= the "activating event" (e.g., worsened pain); ***B***= the "belief system" (i.e., the unhealthy

pattern of thoughts, attitudes and beliefs about pain); and ***C***= the "consequences" of the event (e.g., negative emotions or avoidance behaviors arising from the maladaptive belief system about pain).

Appendix B: Core Outcome Measures

AIM 1- PAIN SEVERITY: BRIEF PAIN INVENTORY

Mark your responses to the questions below:

1. Please rate your pain by circling the one number that best describes your pain at its **WORST** in the last week.

0	1	2	3	4	5	6	7	8	9	10
No Pain										Pain as bad as you can imagine

2. Please rate your pain by circling the one number that best describes your pain at its **LEAST** in the last week.

0	1	2	3	4	5	6	7	8	9	10
No Pain										Pain as bad as you can imagine

3. Please rate your pain by circling the one number that best describes your pain on the **AVERAGE**.

0	1	2	3	4	5	6	7	8	9	10
No Pain										Pain as bad as you can imagine

4. Please rate your pain by circling the one number that tells how much pain you have **RIGHT NOW**.

0	1	2	3	4	5	6	7	8	9	10
No Pain										Pain as bad as you can imagine

AIM 1- PHYSICAL FUNCTION: OSWESTRY DISABILITY INDEX

This questionnaire is designed to give us information as to how your back trouble affects your ability to manage in everyday life. Please answer every section. Mark **ONE** box only in each section that most closely describes you **TODAY**.

SECTION 1 – PAIN INTENSITY

- ❑ I have no pain at the moment.
- ❑ The pain is very mild at the moment.
- ❑ The pain is moderate at the moment.
- ❑ The pain is fairly severe at the moment.
- ❑ The pain is very severe at the moment.
- ❑ The pain is the worst imaginable at the moment.

SECTION 2 - PERSONAL CARE (WASHING, DRESSING, ETC.)

- ❑ I can look after myself normally without causing additional pain.
- ❑ I can look after myself normally but it is very painful.
- ❑ It is painful to look after myself and I am slow and careful.
- ❑ I need some help but manage most of my personal care.
- ❑ I need help every day in most aspects of my personal care.
- ❑ I do not get dressed, I wash with difficulty and stay in bed.

SECTION 3 - LIFTING

- ❑ I can lift heavy weights without additional pain.
- ❑ I can lift heavy weights but it gives me additional pain.
- ❑ Pain prevents me from lifting heavy weights off the floor but I can manage if they are conveniently positioned, e.g. on a table.
- ❑ Pain prevents me from lifting heavy weights but I can manage light to medium weights if they are conveniently positioned.

- ❑ I can only lift very light weights.
- ❑ I cannot lift or carry anything at all.

SECTION 4 - WALKING

- ❑ Pain does not prevent me from walking any distance.
- ❑ Pain prevents me from walking more than one mile.
- ❑ Pain prevents me from walking more than a quarter of a mile.
- ❑ Pain prevents me from walking more than 100 yards.
- ❑ I can only walk using a cane or crutches.
- ❑ I am in bed most of the time and have to crawl to the toilet.

SECTION 5 - SITTING

- ❑ I can sit in any chair as long as I like.
- ❑ I can sit in my favorite chair as long as I like.
- ❑ Pain prevents me from sitting for more than 1 hour.
- ❑ Pain prevents me from sitting for more than half an hour.
- ❑ Pain prevents me from sitting for more than 10 minutes.
- ❑ Pain prevents me from sitting at all.

SECTION 6 - STANDING

- ❑ I can stand as long as I want without additional pain.
- ❑ I can stand as long as I want but it gives me additional pain.
- ❑ Pain prevents me from standing for more than 1 hour.
- ❑ Pain prevents me from standing for more than half an hour.
- ❑ Pain prevents me from standing for more than 10 minutes.
- ❑ Pain prevents me from standing at all.

SECTION 7 - SLEEPING

- ❑ My sleep is never interrupted by pain.
- ❑ My sleep is occasionally interrupted by pain.
- ❑ Because of pain I have less than 6 hours sleep.
- ❑ Because of pain I have less than 4 hours sleep.
- ❑ Because of pain I have less than 2 hours sleep.
- ❑ Pain prevents me from sleeping at all.

SECTION 8 - SEX LIFE (IF APPLICABLE)

- ❑ My sex life is normal and causes no additional pain.
- ❑ My sex life is normal but causes some additional pain.
- ❑ My sex life is nearly normal but is very painful.
- ❑ My sex life is severely restricted by pain.
- ❑ My sex life is nearly non existent because of pain.
- ❑ Pain prevents me from having any sex life at all.

SECTION 9 - SOCIAL LIFE

- ❑ My social life is normal and causes me no additional pain.
- ❑ My social life is normal but increases the degree of pain.
- ❑ Pain has no significant effect on my social life apart from limiting my more energetic interests, e.g. sport, etc.
- ❑ Pain has restricted my social life and I do not go out as often.
- ❑ Pain has restricted my social life to home.
- ❑ I have no social life because of pain.

SECTION 10 – TRAVELING

- ❑ I can travel anywhere without pain.
- ❑ I can travel anywhere but it gives me additional pain.
- ❑ Pain is bad but I am able to manage trips over two hours.
- ❑ Pain restricts me to trips of less than one hour.

- ❑ Pain restricts me to short necessary trips of under 30 minutes.
- ❑ Pain prevents me from traveling except to receive treatment.

AIM 2 - QUALITY OF LIFE: SF-12® SHORT FORM

This survey asks for your views about your health. This information will help you keep track of how you feel and how well you are able to do your usual activities. Answer every question by selecting the answer as indicated. If you are unsure about how to answer a question, please give the best answer you can.

1. In general, would you say your health is	Excellent (1)	Very good (2)	Good (3)	Fair (4)	Poor (5)

2 The following questions are about activities you might do during a **typical day**. Does your health now limit you in these activities?			
a. Moderate activities, such as moving a table, pushing a vacuum cleaner, bowling, or playing golf	Yes, Limited a Lot (1)	Yes, Limited a Little (2)	No, Not limited at All (3)
b. Climbing several flights of stairs	Yes, Limited a Lot (1)	Yes, Limited a Little (2)	No, Not limited at All (3)

3. During the past 4 weeks, have you had any of the following problems with your work or other regular daily activities as a result of your physical health?		
a. Accomplished less than you would like	Yes (1)	No (2)
b. Were limited in the kind of work or other activities	Yes (1)	No (2)

4. During the past 4 weeks**,** have you had any of the following problems with your work or other regular daily activities as a result of any emotional problems (such as feeling depressed or anxious)?		
a. Accomplished less than you would like	Yes (1)	No (2)
b. Did work or other activities less carefully than usual	Yes (1)	No (2)

5. During the **past 4 weeks,** how much did pain interfere with your normal work (including both work outside the home and housework)?	Not at all (1)	Slightly (2)	Moderately (3)	Quite a bit (4)	Extremely (5)

6. These questions are about how you feel and how things have been with you during the past 4 weeks. For each question, please give the one answer that comes closest to the way you have been feeling. How much of the time during the past 4 weeks…						
a. Have you felt calm and peaceful?	All of the Time (1)	Most of the Time (2)	A Good Bit of the Time (3)	Some of the Time (4)	A little of the Time (5)	None of the Time (6)
b. Did you have a lot of energy?	All of the Time (1)	Most of the Time (2)	A Good Bit of the Time (3)	Some of the Time (4)	A little of the Time (5)	None of the Time (6)
c. Have you felt downhearted and blue?	All of the Time (1)	Most of the Time (2)	A Good Bit of the Time (3)	Some of the Time (4)	A little of the Time (5)	None of the Time (6)

7. During the past 4 weeks, how much of the time has your physical health or emotional problems interfered with your social activities (like visiting with friends, relatives, etc.)?	All of the time (1)	Most of the time (2)	Some of the time (3)	A little of the time (4)	None of the time (5)

AIM 2 – OPIOID DOSE (PAST 2 WEEKS): TIMELINE FOLLOWBACK METHOD

LIST OF MEDICATIONS *(past 2 weeks)*

Opioid (narcotic) medication name	Strength per one "unit" (tablet, capsule, film, etc.)	Is this medication supposed to be taken "on schedule" every day or only when needed? (mark the appropriate box)
For example: morphine sulfate ER	*For example:* 15 mg/tablet	❑ *scheduled (to be taken every day in the same way)*<br>❑ *as-needed (to be taken ONLY when needed)*
		❑ *scheduled (to be taken every day in the same way)*<br>❑ *as-needed (to be taken ONLY when needed)*
		❑ *scheduled (to be taken every day in the same way)*<br>❑ *as-needed (to be taken ONLY when needed)*

TIMELINE FOLLOWBACK SURVEY (one week example; in the final form, the TLFB appears in as "calendar")

Choose the medication from the list above; how many of the medication "units" have you taken on each of the following days:

Day of the week (start with yesterday)	Scheduled opioid (narcotic) medications: the ones that are supposed to be taken every day	As-needed opioid (narcotic) medications: the ones that are supposed to be taken ONLY if needed
Sunday (yesterday's date)		
Saturday (2 days ago)		
Friday (3 days ago)		
Thursday (4 days ago)		
Wednesday (5 days ago)		
Tuesday (6 days ago)		
Monday (7 days ago)		

AIM 3 – OPIOID MISUSE: OPIOID COMPLIANCE CHECKLIST

Over the past month have you:

1. Taken your opioid medication other than the way they were prescribed? Yes No
2. Used more than one pharmacy to fill your opioid prescriptions? Yes No
3. Received opioid prescriptions from more than one provider? Yes No
4. Lost or misplaced your opioid medication? Yes No
5. Run out of your pain medication early? Yes No
6. Missed any scheduled medical appointments? Yes No
7. Borrowed opioid medication from others? Yes No
8. Used any illegal substances? Yes No

If you answered yes to any of the above questions please explain below.

AIM 3 – OPIOID MISUSE: CURENT OPIOID MISUSE MEASURE

Please answer each question as honestly as possible. Keep in mind that we are only asking about the **past 30 days**. There are no right or wrong answers. If you are unsure about how to answer the question, please give the best answer you can

**Please answer the questions using the following scale:**	Never (0)	Seldom (1)	Sometimes (2)	Often (3)	Very Often (4)
1. In the past 30 days, how often have you had trouble with thinking clearly or had memory problems?					
2. In the past 30 days, how often do people complain that you are not completing necessary tasks? (i.e., doing things that need to be done, such as going to class, work or appointments)					
3. In the past 30 days, how often have you had to go to someone other than your prescribing physician to get sufficient pain relief from medications? (i.e., another doctor, the Emergency Room, friends, street sources)					
4. In the past 30 days, how often have you taken your medications differently from how they are prescribed?					
5. In the past 30 days, how often have you seriously thought about hurting yourself?					
6. In the past 30 days, how much of your time was spent thinking about opioid medications (having enough, taking them, dosing schedule, etc.)?					
7. In the past 30 days, how often have you been in an argument?					
8. In the past 30 days, how often have you had trouble controlling your anger (e.g., road rage, screaming, etc.)?					
9. In the past 30 days, how often have you needed to take pain medications belonging to someone else?					
10. In the past 30 days, how often have you been worried about how you're handling your medications?					
11. In the past 30 days, how often have others been worried about how you're handling your medications?					
12. In the past 30 days, how often have you had to make an emergency phone call or show up at the clinic without an appointment?					
13. In the past 30 days, how often have you gotten angry with people?					
14. In the past 30 days, how often have you had to take more of your medication than prescribed?					
15. In the past 30 days, how often have your borrowed pain medication from someone else?					
16. In the past 30 days, how often have you used your pain medicine for symptoms other than for pain (e.g., to help you sleep, improve your mood, or relieve stress)?					
17. In the past 30 days, how often have you had to visit the Emergency Room?					

AIM 3 – NEGATIVE AFFECT: HOSPITAL ANXIETY AND DEPRESSION SCALE

Tick the box beside the reply that is closest to how you have been feeling in the past week. Don’t take too long over your replies: your immediate is best.

	**I feel tense or 'wound up':**		**I feel as if I am slowed down:**
	Most of the time		Nearly all the time
	A lot of the time		Very often
	From time to time, occasionally		Sometimes
	Not at all		Not at all
	**I still enjoy the things I used to enjoy:**		**I get a sort of frightened feeling like 'butterflies' in the stomach:**
	Definitely as much		Not at all
	Not quite so much		Occasionally
	Only a little		Quite Often
	Hardly at all		Very Often
	**I get a sort of frightened feeling as if something awful is about to happen:**		**I have lost interest in my appearance:**
	Very definitely and quite badly		Definitely
	Yes, but not too badly		I don't take as much care as I should
	A little, but it doesn't worry me		I may not take quite as much care
	Not at all		I take just as much care as ever
	**I can laugh and see the funny side of things:**		**I feel restless as I have to be on the move:**
	As much as I always could		Very much indeed
	Not quite so much now		Quite a lot
	Definitely not so much now		Not very much
	Not at all		Not at all
	**Worrying thoughts go through my mind:**		**I look forward with enjoyment to things:**
	A great deal of the time		As much as I ever did
	A lot of the time		Rather less than I used to
	From time to time, but not too often		Definitely less than I used to
	Only occasionally		Hardly at all
	**I feel cheerful:**		**I get sudden feelings of panic:**
	Not at all		Very often indeed
	Not often		Quite often
	Sometimes		Not very often
	Most of the time		Not at all
	**I can sit at ease and feel relaxed:**		**I can enjoy a good book or radio or TV program:**
	Definitely		Often
	Usually		Sometimes
	Not Often		Not often
	Not at all		Very seldom

Please check you have answered all the questions

Community Advisors on Research Design and Strategies (CARDS)®

The Community Advisors on Research Design and Strategies (CARDS)® is one of the services offered by the Wisconsin Network for Research Support (WINRS), an innovative patient and community engagement center, based at the University of Wisconsin-Madison School of Nursing. WINRS was established in 2010 with funding from a 3-year NIH program grant, *Building Sustainable Community-Linked Infrastructure to Support Health Sciences Research*. The primary goal of WINRS is to address a persistent problem for researchers, health educators, and health systems administrators – how to effectively engage participants, especially people from under-represented communities or "hard to reach" populations, in research, patient education, and quality improvement initiatives. The CARDS® program is the result of a close collaboration between WINRS and two community centers in Madison, WI. Members of the CARDS® groups were recruited by community center staff from the people who use center services, such as parenting programs, the food pantry, and senior or women's groups. The CARDS® have all completed a training developed by WINRS to prepare them to offer researchers effective feedback on a wide range of materials, including survey, interview and focus group questions; websites; recruitment materials and plans; and smartphone apps. Since 2010, the CARDS® have met with over 100 researchers from many disciplines, including Nursing, Surgery, Pharmacy, Family Practice, and Internal Medicine.

WINRS staff plan and facilitate 90-minute meetings each month between researchers and the CARDS®. WINRS staff take care of all meeting logistics, including attendance, stipends, transportation, room rental, and childcare. Before each CARDS® meeting, WINRS consults with the research team to prepare an agenda that will assure maximum value from meeting with the Community Advisors. During the meeting, researchers present their material to the group and receive immediate, detailed feedback.

The CARDS® comments generally focus on:

- How to make study materials more inviting, easy to read, and easy to understand
- How to revise strategies, plans, or wording to avoid provoking unintended offense or fear and to avoid pitfalls that might discourage people from participating in the research project

The WINRS staff facilitate the meeting and take notes. Following the meeting, WINRS staff prepare a written summary of the CARDS® comments and revise the research materials based on the CARDS® feedback. WINRS shares the summary reports with the research team, who can use this input in whatever ways are most beneficial to their research project.

Currently, there are 16 people serving as trained Community Advisors with the CARDS® program:

- 10 women, 6 men
- 11 African-Americans, 5 Whites
- Age range from 20s to 70s

The CARDS® program is the product of an NIH grant (Building Sustainable Community-Linked Infrastructure to Support Health Sciences Research through American Recovery and Reinvestment Act) -- award number RC4NR012372 from the National Institute of Nursing Research and is supported in part by the CTSA program through NCATS, grant 9U54TR000021.
