# Supplementary material for: Mindfulness vs Cognitive Behavioral Therapy for Chronic Low Back Pain Treated With Opioids: A Randomized Clinical Trial
Source: JAMA Netw Open. 2025 Apr 7;8(4):e253204. doi: 10.1001/jamanetworkopen.2025.3204 (PMC11976494; doi:10.1001/jamanetworkopen.2025.3204)
Supplement: Supplement 2. — Original Statistical Analysis Plan, Modification, and Final Statistical Analysis Plan Summary [file jamanetwopen-e253204-s002.pdf]

Original Statistical Analysis Plan (SAP):

Primary Analysis Plan

The primary analysis will be based on the intent-to-treat principle, including all randomized participants regardless of adherence to assigned treatment. Descriptive statistics will be calculated on demographic variables and baseline outcome measures. The primary analysis will be tested in the framework of linear mixed effects to examine the comparative effectiveness of treatment on the change in pain severity, function (Aim 1) and QoL scores and opioid dose (Aim 2) over 12-months between the two groups. Tests will be performed at a two-tailed significance level of 0.025 for each co-primary outcome (Aim 1) and a 0.05 significance level for the secondary outcomes (Aim 2). Included in each model will be dummy variable for treatment, an effect for each of time and site, time by treatment interaction, and a random intercept for subject. Contrasts will be used to test for an overall treatment effect at 6 and 12 months. Subsequent to primary analyses, for any outcome found to be statistically significant, the effect at each follow-up will be tested. The method of maximum likelihood will be used to estimate the model parameters in the linear mixed models.

Missing data: Although we will monitor and enhance retention and protocol adherence, and monitor data quality during collection, entry and maintenance, we anticipate some missing data. The maximum likelihood method will account for missing data on main outcomes; this approach is expected to be more robust to missing data under missingness at random (MAR) assumption than non-likelihood approaches, such as generalized estimating equations.[1] Missing data on covariates will be imputed using multiple imputation (i.e., chained equations) technique, which produces valid statistical estimates under MAR and accounts for the uncertainty arising from missing data.[2,3] If a participant drops out, we will document the reason and whether the dropout involves some or all types of participation. We will attempt to contact study subjects to collect data on at least the primary and secondary outcomes. All subjects will be accounted for in the reports.

Sensitivity analysis: Using multiple imputation techniques, we will assess the degree to which results are sensitive to the MAR assumption.[4] To assess the normality assumption of the linear

mixed models normal q-q plots of the residuals will be used. To examine the sensitivity to model assumptions, robust nonparametric method[5] will be used to evaluate treatment effects without distributional assumptions; if the results differ from those of the maximum likelihood approach both sets will be reported, else only the maximum likelihood results will be reported.

Modifications to the original SAP:

The following non-inferiority analysis plan was approved and added to the SAP in 2023:

If the primary superiority analysis does not find significant differences between the two groups for either of the two co-primary outcomes, a secondary non-inferiority analysis will be conducted. That non-inferiority analysis would employ linear mixed effects models to determine 95% confidence intervals for the difference between the mean scores of the co-primary outcomes of pain and function, with the purpose of assessing whether mindfulness-based therapy is non-inferior to cognitive behavioral therapy.[6,7] These analyses will use acceptable difference delta values of 0.8 for the BPI pain scale and 8.0 for the ODI function scale, which are (1) consistent with best literature including IMMPACT recommendations, (2) less than the proposed superiority margins of 1.0 for BPI and 10.0 for ODI, and (3) suggested to us by external "arms length" experts while both they and we were completely blinded to trial results.[8]

References:

1. Beunckens C, Molenberghs G, Kenward MG. Direct likelihood analysis versus simple forms of imputation for missing data in randomized clinical trials. Clin Trials, 2005, 2(5): 379-86. doi: 10.1191/1740774505cn119oa

2. White IR, Royston P, Wood AM. Multiple imputation using chained equations: Issues and guidance for practice. Stat Med, 2011, 30(4): 377-399.

3. Schafer JL. Analysis of incomplete multivariate data, 1st Edition. New York: Chapman and Hall, 1997, https://doi.org/10.1201/9780367803025

4. Carpenter JR, Kenward MG, White IR. Sensitivity analysis after multiple imputation under missing at random: a weighting approach. Stat Methods Med Res, 2007, 16(3): 259-275. doi: 10.1177/0962280206075303

5. Kloke JD, Mckean JW, Rashid MM. Rank-Based Estimation and Associated Inferences for Linear Models With Cluster Correlated Errors. J Am Stat Assoc, 2009, 104(485): 384-390. doi: 10.1198/jasa.2009.0116

6. US Food and Drug Administration. Non-inferiority clinical trials to establish effectiveness: Guidance for industry. U.S. Department of Health and Human Services, Food and Drug Administration, Center for Drug Evaluation and Research, and Center for Biologics Evaluation and Research, November 2016. Accessed on Oct 5, 2024 at: https://www.fda.gov/media/78504/download

7. Emanuel EJ. Committee for Proprietary Medicinal Products. Points to consider on switching between superiority and non-inferiority. Br J Clin Pharmacol, 2001, 52(3): 223-228. doi: 10.1046/j.0306-5251.2001.01397-3.x

8. Dworkin RH, Turk DC, Wyrwich KW, et al. Interpreting the clinical importance of treatment outcomes in chronic pain clinical trials: IMMPACT recommendations. J Pain, 2008, 9(2): 105-121. doi: 10.1016/j.jpain.2007.09.005

Final SAP summary

Descriptive statistics were applied to characterize each study group at each time point, and to characterize the participants who completed the study (i.e., provided main outcome data at 12 months) and those “lost-to-follow-up” (i.e., did not complete their 12-month assessment). Results are presented as means (standard deviation) or numbers (percentage) unless otherwise indicated. We used p values computed under the false discovery rate (FDR) when completing numerous comparisons to reduce the number of potential Type 1 errors.

Comparison of baseline data between the MBT and CBT groups assessed the randomization success, and for the differences between participants who completed the study versus those who were lost-to-follow-up.

The ***primary effectiveness analysis*** was based on the intent-to-treat principle. We used a linear mixed effects modelling framework to examine the comparative effectiveness of each treatment on the change score in pain severity and function (Aim 1), with contrasts at 6 and 12 months to test for primary outcome treatment effect. Similar models assessed secondary outcomes of QoL scores and daily opioid dose (Aim 2). Hypothesis-testing was based on a two-tailed significance level of $p<0.025$ for each co-primary outcome (Aim 1) and a $p<0.05$ significance level for the secondary outcomes (Aim 2). Per our *a priori* statistical analysis plan,[1] each model included a dummy variable for treatment, and effect variables for time and site, time x treatment interaction, and a random intercept for participant. Restricted maximum likelihood estimation was used to assess model parameters in the linear mixed effects models.

In case the primary superiority analysis did not detect significant differences between the two groups for either of the two co-primary outcomes (average pain severity – one item; ODI-based functional limitations) at 6 and 12 months, a secondary pre-determined non-inferiority analysis would be applied. Non-inferiority analysis would be based on 95% CIs of the pain and function co-primary outcomes' change scores (baseline to 6 months, baseline to 12 months) derived from the linear mixed effects model analysis used for superiority testing. When determining non-inferiority, the margin or degree of acceptable difference (delta) at 6 and 12 months would be 0.8 for the pain severity score and 8.0 for the ODI function score.[2-4]

With the application of a linear mixed effects model analysis, we did not deem it necessary to perform multiple imputation.[5,6] We imputed missing values within an individual survey in order to calculate each survey's composite score if at least 75% of responses within a given survey were provided (i.e., we imputed not more than 25% of missing responses per survey).[7] However, if composite score calculations were pre-defined by the scale developers to, for example, take the mean of available data, means were assessed without imputation. The restricted maximum likelihood estimates from the linear mixed effects model yielded valid

estimates in the presence of missing at random (MAR) data.[8] To assess the normality assumption of the linear mixed effects models, normal q-q plots of the residuals were used. For non-normally distributed variables, transformation or nonparametric methods[9] were considered for evaluation of treatment effects without distributional assumptions. As a sensitivity analysis, we also applied non-parametric methods for outcomes that appeared to be normally distributed. The analyses were completed using the R statistical analysis software (Version 4.3.1, Core Team 2023).

References:

1. Zgierska AE, Burzinski CA, Garland EL, et al. Mindfulness-based therapy compared to cognitive behavioral therapy for opioid-treated chronic low back pain: Protocol for a pragmatic randomized controlled trial. Contemp Clin Trials, 2021, 110: 106548. doi: 10.1016/j.cct.2021.106548

2. Committee for Proprietary Medicinal Products. Points to consider on switching between superiority and non-inferiority. Br J Clin Pharmacol, 2001, 52(3): 223-228. doi: 10.1046/j.0306-5251.2001.01397-3.x

3. Dworkin RH, Turk DC, Wyrwich KW, et al. Interpreting the clinical importance of treatment outcomes in chronic pain clinical trials: IMMPACT recommendations. J Pain, 2008, 9(2): 105-121. doi: 10.1016/j.jpain.2007.09.005

4. US Food and Drug Administration. Non-inferiority clinical trials to establish effectiveness: Guidance for industry. U.S. Department of Health and Human Services, Food and Drug Administration, Center for Drug Evaluation and Research, and Center for Biologics Evaluation and Research, November 2016. Accessed on Oct 5, 2024 at: https://www.fda.gov/media/78504/download

5. Schafer JL. Analysis of incomplete multivariate data, 1st Edition. New York: Chapman and Hall, 1997, https://doi.org/10.1201/9780367803025

6. White IR, Royston P, Wood AM. Multiple imputation using chained equations: Issues and guidance for practice. Stat Med, 2011, 30(4): 377-399. doi: 10.1002/sim.4067

7. Jadhav A, Pramod D, Ramanathan K. Comparison of Performance of Data Imputation Methods for Numeric Dataset, Appl Artif Intell, 2019, 33 (10): 913-933. doi: 10.1080/08839514.2019.1637138

8. Carpenter JR, Kenward MG, White IR. Sensitivity analysis after multiple imputation under missing at random: a weighting approach. Stat Methods Med Res, 2007, 16(3): 259-275. doi: 10.1177/0962280206075303

9. Kloke JD, McKean JW, Rashid MM. Rank-Based Estimation and Associated Inferences for Linear Models With Cluster Correlated Errors. J Am Stat Assoc, 2009, 104(485): 384-390. doi: 10.1198/jasa.2009.0116
