# Supplementary material for: Mindfulness vs Cognitive Behavioral Therapy for Chronic Low Back Pain Treated With Opioids: A Randomized Clinical Trial
Source: JAMA Netw Open. 2025 Apr 7;8(4):e253204. doi: 10.1001/jamanetworkopen.2025.3204 (PMC11976494; doi:10.1001/jamanetworkopen.2025.3204)
Supplement: Supplement 3. — eTable 1. Additional Baseline Characteristics of the Study Participants: Total Sample and by Group Status eTable 2. Change in Main Outcomes (Raw Values and Difference Scores) Over Time by Group Status eTable 3. Linear Mixed Effects Model (Primary Outcome Analysis): Change in Main Outcomes From Baseline in the MBT vs the CBT Group eTable 4. Primary Outcomes Over Time (Estimated Marginal Means and SEs): Variables for Figure 2 eFigure. Between-Group Differences in Primary Outcomes Over Time: Noninferiority Analysis [file jamanetwopen-e253204-s003.pdf]

Supplementary Online Content

Zgierska AE, Edwards RR, Barrett B, et al. Mindfulness vs cognitive behavioral therapy for chronic low back pain treated with opioids: a randomized clinical trial. *JAMA Netw Open.* 2025;8(4):e253204. doi:10.1001/jamanetworkopen.2025.3204

eTable 1. Additional Baseline Characteristics of the Study Participants: Total Sample and by Group Status
eTable 2. Change in Main Outcomes (Raw Values and Difference Scores) Over Time by Group Status
eTable 3. Linear Mixed Effects Model (Primary Outcome Analysis): Change in Main Outcomes From Baseline in the MBT vs the CBT Group
eTable 4. Primary Outcomes Over Time (Estimated Marginal Means and SEs): Variables for Figure 2
eFigure. Between-Group Differences in Primary Outcomes Over Time: Noninferiority Analysis

This supplementary material has been provided by the authors to give readers additional information about their work.

© 2025 Zgierska AE et al. *JAMA Network Open*.

eTable 1. Additional Baseline Characteristics of the Study Participants: Total Sample and by Group Status

Variable	Total (N=770)	MBT (n=385)	CBT (n=385)
**Demographic characteristics**			
**Education**, No. (%)			
No high school diploma	26 (3.38)	15 (3.90)	11 (2.86)
High school graduate or GED	108 (14.03)	51 (13.25)	57 (14.81)
Some college, no degree	184 (23.90)	97 (25.19)	87 (22.60)
Occupational/technical/vocational program	62 (8.05)	32 (8.31)	30 (7.79)
Associate degree: academic program	101 (13.12)	46 (11.95)	55 (14.29)
Bachelor’s degree	166 (21.56)	90 (23.38)	76 (19.74)
Master’s degree	81 (10.52)	35 (9.09)	46 (11.95)
Professional degree	13 (1.69)	4 (1.04)	9 (2.34)
Doctoral degree	15 (1.95)	8 (2.08)	7 (1.82)
**Current relationship status**, No. (%)			
Single, not in a relation	289 (37.53)	145 (37.66)	144 (37.40)
In a relation, not living with partner	54 (7.01)	30 (7.79)	24 (6.23)
In a relationship, living with partner	64 (8.31)	29 (7.53)	35 (9.09)
Married	345 (44.81)	171 (44.42)	174 (45.19)
**Past relationship status**, No. (%)			
Divorced/Separated/Widowed	391 (50.78)	198 (51.43)	193 (50.13)
Neither	351 (45.58)	170 (44.16)	181 (47.01)
**Gross individual income**, No. (%)			
Under $16,000	244 (31.69)	131 (34.03)	113 (29.35)
$16,000-$24,999	134 (17.40)	78 (20.26)	56 (14.55)

© 2025 Zgierska AE et al. *JAMA Network Open*.

$25,000-$34,999	81 (10.52)	28 (7.27)	53 (13.77)
$35,000-$49,999	73 (9.48)	37 (9.61)	36 (9.35)
$50,000-$74,999	63 (8.18)	29 (7.53)	34 (8.83)
$75,000-$99,999	45 (5.84)	20 (5.19)	25 (6.49)
$100,000 and above	48 (6.23)	24 (6.23)	24 (6.23)
**Chronic back pain-related characteristics**			
**CLBP duration**, No. (%)			
1-3 months	1 (0.13)	0 (0.00)	1 (0.26)
3-6 months	5 (0.65)	1 (0.26)	4 (1.04)
6 months-1 year	15 (1.95)	10 (2.60)	5 (1.30)
1-5 years	124 (16.10)	67 (17.40)	57 (14.81)
>5 years	620 (80.52)	306 (79.48)	314 (81.56)
**Back pain spreading down the leg(s)**, past 2 weeks			
Yes	557 (72.34)	283 (73.51%)	274 (71.17%)
No	168 (21.82)	76 (19.74%)	92 (23.90%)
**Off work or unemployed for ≥1 month due to low back pain**			
Agree	416 (54.03)	206 (53.51%)	210 (54.55%)
Disagree	99 (12.86)	59 (15.32%)	40 (10.39%)
**Receiving/applied for disability or workers' compensation due to low back pain**			
Agree	381 (49.48)	200 (51.95%)	181 (47.01%)
Disagree	164 (21.30)	85 (22.08%)	79 (20.52%)
**I feel my back pain is terrible and never going to get any better**			
Agree	389 (50.52)	189 (49.09%)	200 (51.95%)
Disagree	354 (45.97)	183 (47.53%)	171 (44.42%)
**It's not really safe for a person with my back problem to be physically active**			

© 2025 Zgierska AE et al. *JAMA Network Open*.

Agree	260 (33.77)	126 (32.73%)	134 (34.81%)
Disagree	491 (63.77)	247 (64.16%)	244 (63.38%)
**Prior treatments for back pain**			
Percent relief from opioid medications, past 7 days, mean (SD)	56.57 (20.54)	57.55 (20.08)	55.59 (20.96)
History of a low back operation, Yes, No. (%)	327 (42.47)	163 (42.34)	164 (42.60)
Injections (e.g., epidural steroid, facet), Yes, No. (%)	614 (79.74)	304 (78.96)	310 (80.52)
Exercise therapy (physical therapy), Yes, No. (%)	694 (90.13)	348 (90.39)	346 (89.87)
Psychological counseling, Yes, No. (%)	276 (35.84)	130 (33.77)	146 (37.92)
**Cigarette smoking**, No. (%)			
Current	144 (18.70)	67 (17.40)	77 (20.00)
Past (quit)	293 (38.05)	148 (38.44)	145 (37.66)
Never	312 (40.52)	161 (41.82)	151 (39.22)
**Healthcare utilization and productivity-related characteristics (past 6 months)**			
Hospitalization, Yes, No. (%)	146 (19.84)	68 (18.38)	78 (21.31)
Emergency department visit, Yes, No. (%)	248 (33.74)	123 (33.24)	125 (34.25)
Clinic visit, Yes, No. (%)	632 (86.93)	323 (88.01)	309 (85.83)
Mental health care visit, Yes, No. (%)	267 (36.13)	128 (34.32)	139 (37.98)
Addiction care visit, Yes, No. (%)	35 (4.72)	18 (4.88)	17 (4.57)
Whole day of chores (school/house) missed, Yes, No. (%)	458 (69.50)	225 (70.31)	233 (68.73)
Currently working, Yes, No. (%)	138 (18.18)	66 (17.46)	72 (18.90)
**Clinical characteristics**			
**Pain severity** (BPI, 0-10 score), mean (SD)			
**Composite pain severity**, averaged across 4 pain items	6.08 (1.47)	6.01 (1.41)	6.15 (1.53)
**Functional limitations, mean (SD)**			
**Pain interference** (BPI, 0-10 score)	6.48 (1.84)	6.56 (1.79)	6.40 (1.88)

© 2025 Zgierska AE et al. *JAMA Network Open*.

**Daily opioid dose** (TLFB), past 14 days, morphine MG-equivalents (MME)/day			
**MME dose categories**, No. (%)			
< 50 mg/day	415 (53.90)	200 (51.95)	215 (55.84)
50-89 mg/day	117 (15.19)	62 (16.10)	55 (14.29)
90-199 mg/day	142 (18.44)	72 (18.70)	70 (18.18)
≥ 200 mg/day	96 (12.47)	51 (13.25)	45 (11.69)

The frequencies/percentages in a given variable category may not total the sample size per group or 100% responses as some participants' responses were unavailable (e.g., missing or marked as declined, unknown or unspecified).

Acronyms: BPI: Brief Pain Inventory; CBT: Cognitive Behavioral Therapy; CLBP: Chronic Low Back Pain; MBT: Mindfulness-Based Therapy; MME: Morphine-MG Equivalents; No.: number; SD: standard deviation; TLFB: Timeline Followback

© 2025 Zgierska AE et al. *JAMA Network Open*.

eTable 2. Change in Main Outcomes (Raw Values and Difference Scores) Over Time by Group Status

Variable	MBT		CBT	
	Change compared to baseline	*P* value[a] (compared to baseline)	Change compared to baseline	*P* value[a] (compared to baseline)
**Pain severity**				
**Average pain (BPI, one item)**				
**3 months:** score, mean (SD)	5.64 (1.79)		5.78 (1.78)	
Δ score, mean (95% CI)	-0.24 (-0.43, -0.05)	0.01	-0.36 (-0.54, -0.18)	<0.001
**6 months:** score, mean (SD)	5.55 (1.65)		5.54 (1.86)	
Δ score, mean (95% CI)	-0.35 (-0.54, -0.17)	<0.001	-0.57 (-0.76, -0.38)	<0.001
**9 months:** score, mean (SD)	5.50 (1.83)		5.64 (1.76)	
Δ score, mean (95% CI)	-0.40 (-0.59, -0.21)	<0.001	-0.48 (-0.68, -0.29)	<0.001
**12 months:** score, mean (SD)	5.44 (1.85)		5.50 (1.98)	
Δ score, mean (95% CI)	-0.45 (-0.64, -0.26)	<0.001	-0.59 (-0.78, -0.40)	<0.001
**Composite pain (BPI, four items)**				
**3 months:** score, mean (SD)	5.64 (1.71)		5.78 (1.70)	
Δ score, mean (95% CI)	-0.26 (-0.42, -0.10)	0.002	-0.36 (-0.52, -0.19)	<0.001
**6 months:** score, mean (SD)	5.60 (1.67)		5.61 (1.79)	
Δ score, mean (95% CI)	-0.32 (-0.48, -0.16)	<0.001	-0.52 (-0.70, -0.35)	<0.001
**9 months:** score, mean (SD)	5.57 (1.77)		5.64 (1.76)	
Δ score, mean (95% CI)	-0.36 (-0.52, -0.20)	<0.001	-0.49 (-0.67, -0.32)	<0.001
**12 months:** score, mean (SD)	5.48 (1.78)		5.50 (1.86)	
Δ score, mean (95% CI)	-0.42 (-0.58, -0.26)	<0.001	-0.60 (-0.78, -0.43)	<0.001

© 2025 Zgierska AE et al. *JAMA Network Open*.

**Functional limitations**				
**Functional limitations (ODI)**				
**3 months:** score, mean (SD)	45.25 (15.36)		45.20 (15.45)	
Δ score, mean (95% CI)	-1.41 (-2.66, -0.16)	0.03	-1.74 (-3.06, -0.42)	0.01
**6 months:** score, mean (SD)	44.64 (16.05)		45.12 (16.44)	
Δ score, mean (95% CI)	-2.15 (-3.41, -0.89)	0.001	-2.24 (-3.62, -0.86)	0.002
**9 months:** score, mean (SD)	45.60 (15.65)		44.70 (16.48)	
Δ score, mean (95% CI)	-1.41 (-2.68, -0.14)	0.03	-1.71 (-3.09, -0.34)	0.02
**12 months:** score, mean (SD)	43.41 (15.32)		43.17 (15.43)	
Δ score, mean (95% CI)	-3.19 (-4.45, -1.93)	<0.001	-3.49 (-4.86, -2.12)	<0.001
**Pain interference (BPI)**				
**3 months:** score, mean (SD)	5.72 (2.08)		5.73 (2.13)	
Δ score, mean (95% CI)	-0.79 (-1.02, -0.57)	<0.001	-0.61 (-0.84, -0.38)	<0.001
**6 months:** score, mean (SD)	5.75 (2.26)		5.64 (2.33)	
Δ score, mean (95% CI)	-0.75 (-0.98, -0.53)	<0.001	-0.72 (-0.96, -0.47)	<0.001
**9 months:** score, mean (SD)	5.58 (2.28)		5.49 (2.21)	
Δ score, mean (95% CI)	-0.91 (-1.14, -0.69)	<0.001	-0.83 (-1.07, -0.59)	<0.001
**12 months:** score, mean (SD)	5.76 (2.11)		5.39 (2.22)	
Δ score, mean (95% CI)	-0.76 (-0.98, -0.53)	<0.001	-0.90 (-1.14, -0.66)	<0.001
**Health-related quality of life**				
**Mental health component (SF-12)**				
**3 months:** score, mean (SD)	42.89 (11.00)		43.65 (12.40)	
Δ score, mean (95% CI)	1.10 (-0.06, 2.26)	0.06	-0.22 (-1.47, 1.03)	0.72
**6 months:** score, mean (SD)	43.21 (11.42)		43.45 (12.14)	
Δ score, mean (95% CI)	1.39 (0.22, 2.55)	0.02	-0.41 (-1.71, 0.90)	0.54
**9 months:** score, mean (SD)	42.57 (11.20)		44.51 (11.94)	

© 2025 Zgierska AE et al. *JAMA Network Open*.

Δ score, mean (95% CI)	0.85 (-0.32, 2.03)	0.15	0.57 (-0.73, 1.87)	0.38
**12 months:** score, mean (SD)	43.48 (11.48)		45.91 (11.04)	
Δ score, mean (95% CI)	1.91 (0.74, 3.08)	0.001	2.07 (0.78, 3.36)	0.002
**Physical health component (SF-12)**				
**3 months:** score, mean (SD)	29.81 (8.50)		30.34 (8.91)	
Δ score, mean (95% CI)	0 .97 (0.10, 1.84)	0.03	2.05 (1.15, 2.94)	<0.001
**6 months:** score, mean (SD)	30.66 (8.72)		29.94 (9.35)	
Δ score, mean (95% CI)	1.87 (1.00, 2.75)	<0.001	2.04 (1.11, 2.98)	<0.001
**9 months:** score, mean (SD)	30.87 (8.52)		30.47 (9.09)	
Δ score, mean (95% CI)	2.20 (1.32, 3.08)	<0.001	2.17 (1.24, 3.10)	<0.001
**12 months:** score, mean (SD)	30.54 (8.83)		30.00 (9.21)	
Δ score, mean (95% CI)	1.69 (0.82, 2.57)	<0.001	1.66 (0.73, 2.58)	<0.001
**Daily opioid dose**				
**Daily dose (TLFB), log MME/day**				
**3 months:** MME/day, mean (SD)	157.81 (602.32)		192.22 (1,562.31)	
log MME/day, mean (SD)	*5.38 (2.12)*		*5.05 (2.32)*	
Δ log MME/day, mean (95% CI)	-0.33 (-0.49, -0.18)	<0.001	-0.52 (-0.70, -0.35)	<0.001
**6 months:** MME/day, mean (SD)	118.79 (520.99)		191.38 (1,603.24)	
log MME/day, mean (SD)	*5.11 (2.14)*		*4.92 (2.47)*	
Δ log MME/day, mean (95% CI)	-0.54 (-0.69, -0.38)	<0.001	-0.67 (-0.84, -0.49)	<0.001
**9 months:** MME/day, mean (SD)	130.40 (555.63)		198.33 (1,645.71)	
log MME/day, mean (SD)	*5.09 (2.27)*		*4.99 (2.37)*	
Δ log MME/day, mean (95% CI)	-0.58 (-0.74, -0.42)	<0.001	-0.62 (-0.80, -0.44)	<0.001
**12 months:** MME/day, mean (SD)	144.22 (556.19)		192.31 (1,643.92)	
log MME/day, mean (SD)	*4.95 (2.50)*		*4.84 (2.49)*	
Δ log MME/day, mean (95% CI)	-0.79 (-0.95, -0.63)	<0.001	-0.81 (-0.99, -0.64)	<0.001

© 2025 Zgierska AE et al. *JAMA Network Open*.

**≥90 MME/day (TLFB)**				
**3 months:** No. (%)	70 (26.02)		63 (23.86)	
Δ odds ratio, mean (95% CI)	0.39 (0.15, 1.01)	0.05	0.25 (0.09, 0.64)	0.004
**6 months:** No. (%)	64 (23.79)		57 (22.80)	
Δ odds ratio, mean (95% CI)	0.26 (0.10, 0.69)	0.007	0.14 (0.05, 0.39)	<0.001
**9 months:** No. (%)	66 (25.29)		54 (22.78)	
Δ odds ratio, mean (95% CI)	0.36 (0.14, 0.95)	0.03	0.18 (0.07, 0.49)	0.001
**12 months:** No. (%)	65 (25.69)		50 (21.10)	
Δ odds ratio, mean (95% CI)	0.24 (0.09, 0.65)	0.005	0.11 (0.04, 0.32)	<0.001

Acronyms: BPI: Brief Pain Inventory; CBT: Cognitive Behavioral Therapy; CI: confidence interval; MBT: Mindfulness-Based Therapy; MME: Morphine-MG Equivalents; No.: number; ODI: Oswestry Disability Index; SF-12: Medical Outcomes Study Short Form; TLFB: Timeline Followback

© 2025 Zgierska AE et al. *JAMA Network Open*.

eTable 3. Linear Mixed Effects Model (Primary Outcome Analysis): Change in Main Outcomes From Baseline in the MBT vs the CBT Group[a]

**Randomization factor x time factor**<br><br>**(CBT = reference group)**	**3 months**	**6 months**	**9 months**	**12 months**
**Pain severity**				
**Average pain** (BPI, single item)				
estimate (95% CI)	0.12 (-0.15, 0.38)	0.21 (-0.05, 0.48)	0.08 (-0.19, 0.35)	0.13 (-0.13, 0.40)
P value	0.38	0.12	0.54	0.33
**Composite pain** (BPI, four items)				
estimate (95% CI)	0.09 (-0.14, 0.32)	0.20 (-0.04, 0.44)	0.13 (-0.11, 0.37)	0.18 (-0.06, 0.41)
P value	0.43	0.10	0.29	0.14
**Functional limitations**				
**Functional limitations** (ODI)				
estimate (95% CI)	0.31 (-1.50, 2.13)	0.07 (-1.80, 1.93)	0.28 (-1.59, 2.15)	0.27 (-1.59, 2.12)
P value	0.74	0.94	0.77	0.78
**Pain interference** (BPI)				
estimate (95% CI)	-0.19 (-0.51, 0.14)	-0.04 (-0.37, 0.29)	-0.09 (-0.42, 0.24)	0.14 (-0.19, 0.47)
P value	0.26	0.81	0.61	0.39
**Health-related quality of life**				
**Mental health component** (SF-12)				
estimate (95% CI)	1.33 (-0.37, 3.03)	1.79 (0.05, 3.54)	0.29 (-1.46, 2.04)	-0.15 (-1.89, 1.59)
P value	0.13	0.04	0.74	0.86
**Physical health component** (SF-12)				
estimate (95% CI)	-1.07 (-2.31, 0.17)	-0.16 (-1.44, 1.12)	0.04 (-1.24, 1.32)	0.04 (-1.23, 1.32)
P value	0.09	0.81	0.95	0.95

© 2025 Zgierska AE et al. *JAMA Network Open*.

Daily opioid dose (past 14 days)				
**MME/day** (TLFB), log-transformed				
estimate (95% CI)	0.19 (-0.05, 0.42)	0.13 (-0.11, 0.36)	0.04 (-0.20, 0.28)	0.02 (-0.22, 0.26)
P value	0.12	0.29	0.76	0.88
**MME ≥ 90 mg/day** (TLFB)				
Odds Ratio (95% CI)	0.44 (0.13, 1.57)	0.58 (0.16, 2.17)	0.80 (0.22, 2.99)	0.76 (0.19, 2.99)
P value	0.21	0.42	0.74	0.70

Acronyms: BPI: Brief Pain Inventory; CBT: Cognitive Behavioral Therapy; CI: confidence interval; MBT: Mindfulness-Based Therapy

[a] The model included a dummy variable for treatment, an effect for each of time and site, time by treatment interaction, and a random intercept for subject.

© 2025 Zgierska AE et al. *JAMA Network Open*.

eTable 4. Primary Outcomes Over Time (Estimated Marginal Means and SEs): Variables for Figure 2

**Follow-up Assessment**		**Baseline**	**3 months**	**6 months**	**9 months**	**12 months**
**Variables for Figure 2A: Average pain**						
estimated marginal mean (standard error)	MBT	6.00 (0.09)	5.75 (0.10)	5.64 (0.10)	5.60 (0.10)	5.54 (0.10)
	CBT	6.12 (0.09)	5.83 (0.10)	5.62 (0.11)	5.71 (0.11)	5.60 (0.10)
**Variables for Figure 2B: Functional limitations**						
estimated marginal mean (standard error)	MBT	47.34 (0.77)	45.95 (0.84)	45.22 (0.85)	45.97 (0.85)	44.17 (0.85)
	CBT	47.07 (0.77)	45.37 (0.86)	44.89 (0.87)	45.42 (0.87)	43.64 (0.86)

© 2025 Zgierska AE et al. *JAMA Network Open*.

eFigure. Between-Group Differences in Primary Outcomes Over Time: Noninferiority Analysis

A. Average pain scores: The non-inferiority analysis did not find statistically significant between-group differences at any follow-up timepoint.

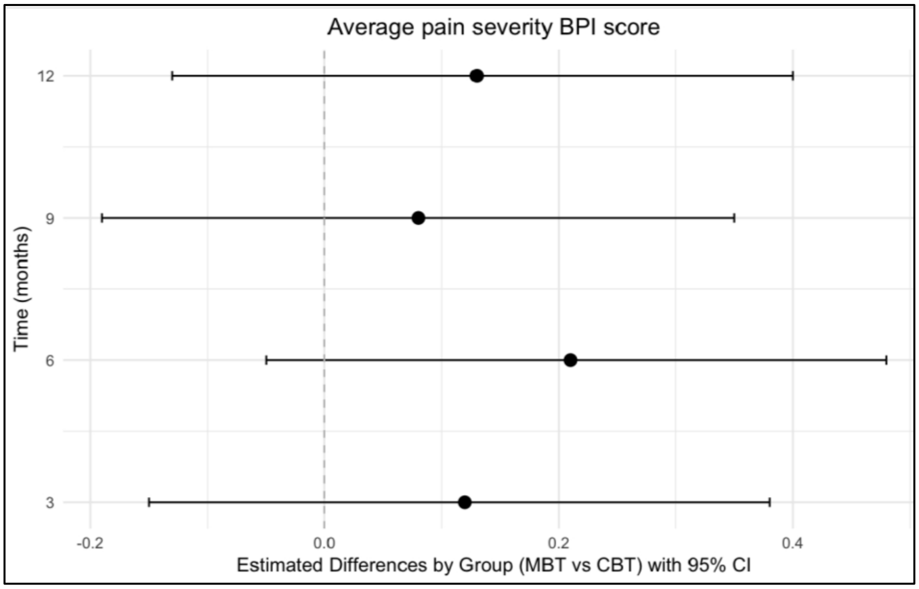

BPI: Brief Pain Inventory; CBT: Cognitive Behavioral Therapy group; MBT: Mindfulness-Based Therapy group

© 2025 Zgierska AE et al. *JAMA Network Open*.

B. Functional limitation scores: The non-inferiority analysis did not find statistically significant between-group differences at any follow-up timepoint.

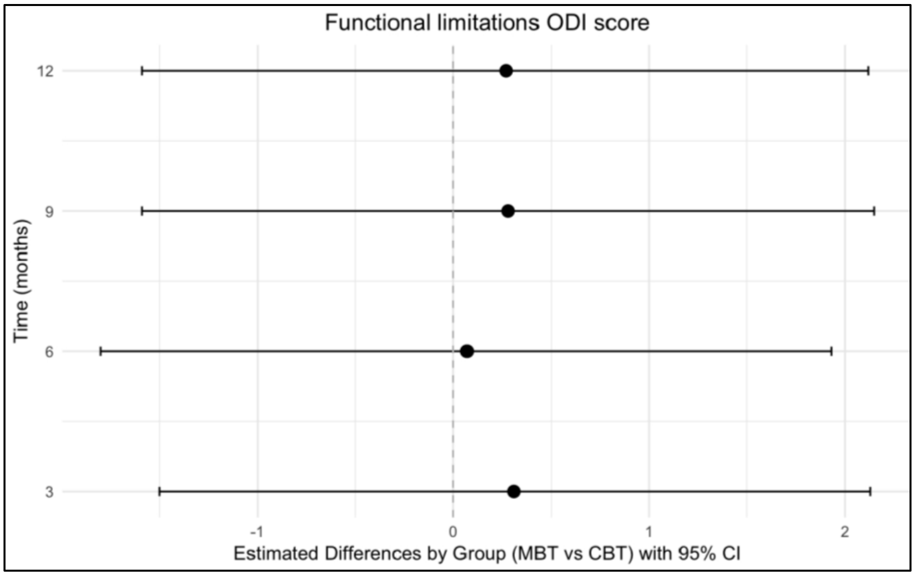

CBT: Cognitive Behavioral Therapy group; MBT: Mindfulness-Based Therapy group; ODI: Oswestry Disability Index

© 2025 Zgierska AE et al. *JAMA Network Open*.
