# Supplementary material for: Mindfulness vs Cognitive Behavioral Therapy for Chronic Low Back Pain Treated With Opioids: A Randomized Clinical Trial
Source: JAMA Netw Open. 2025 Apr 7;8(4):e253204. doi: 10.1001/jamanetworkopen.2025.3204 (PMC11976494; doi:10.1001/jamanetworkopen.2025.3204)
Supplement: Supplement 4. — Data Sharing Statement [file jamanetwopen-e253204-s004.pdf]

Data Sharing Statement

Zgierska. Mindfulness vs Cognitive Behavioral Therapy for Chronic Low Back Pain Treated With Opioids. *JAMA Netw Open*. Published April 07, 2025.
doi:10.1001/jamanetworkopen.2025.3204

Data
Additional Information: ClinicalTrials.gov ID: NCT03115359
Data available: Yes
Data types: Deidentified participant data
How to access data: PCORI's registry (when becomes available)
When available: With publication

Supporting Documents
Document types: Other (please specify)
Additional Information: Protocol and statistical analysis plan attached as a supplemental material.
How to access documents: PCORI's registry (when becomes available), author
When available: With publication

Additional Information
Who can access the data: Public, per PCORI's guidance
Types of analyses: Any
Mechanisms of data availability: Per PCORI's registry guidance
